# Correlated evolution of social organization and lifespan in mammals

**DOI:** 10.1038/s41467-023-35869-7

**Published:** 2023-01-31

**Authors:** Pingfen Zhu, Weiqiang Liu, Xiaoxiao Zhang, Meng Li, Gaoming Liu, Yang Yu, Zihao Li, Xuanjing Li, Juan Du, Xiao Wang, Cyril C. Grueter, Ming Li, Xuming Zhou

**Affiliations:** 1 CAS Key Laboratory of Animal Ecology and Conservation Biology, Institute of Zoology, Beijing, 100101 China; 2 University of Chinese Academy of Sciences, Beijing, 100049 China; 3 Division of Life Sciences and Medicine, University of Science and Technology of China, Hefei, 230026 China; 4 School of Human Sciences, The University of Western Australia, Perth, WA 6009 Australia; 5 Centre for Evolutionary Biology, School of Biological Sciences, The University of Western Australia, Perth, WA 6009 Australia; 6 International Center of Biodiversity and Primate Conservation, Dali University, Dali, Yunnan 671003 China; 7 Center for Excellence in Animal Evolution and Genetics, Chinese Academy of Sciences, Kunming, 650223 China

**Keywords:** Gene expression, Social evolution, Transcriptomics, Social behaviour, Ageing

## Abstract

Discerning the relationship between sociality and longevity would permit a deeper understanding of how animal life history evolved. Here, we perform a phylogenetic comparative analysis of ~1000 mammalian species on three states of social organization (solitary, pair-living, and group-living) and longevity. We show that group-living species generally live longer than solitary species, and that the transition rate from a short-lived state to a long-lived state is higher in group-living than non-group-living species, altogether supporting the correlated evolution of social organization and longevity. The comparative brain transcriptomes of 94 mammalian species identify 31 genes, hormones and immunity-related pathways broadly involved in the association between social organization and longevity. Further selection features reveal twenty overlapping pathways under selection for both social organization and longevity. These results underscore a molecular basis for the influence of the social organization on longevity.

## Abstract (web summary)

To elucidate the relationship between sociality and longevity, the authors perform phylogenetic and transcriptomic comparative analysis of mammals. They find that group-living species lived longer than solitary species and identify 31 genes, hormones, and immunity-related pathways involved in this connection.

## Introduction

Extant mammals exhibit a wide diversity of grouping arrangements or social organizations, including solitary living, pair-living, and various forms of group-living^1^, e.g., multilevel society (e.g., *Rhinopithecus* spp.)^2^ and eusociality (e.g., *Heterocephalus glaber*)^3^. Mammals also show an extreme 100-fold variation in maximum lifespan (or longevity), ranging from ~2 years in shrews (e.g., *Sorex* spp.) to more than 200 years in bowhead whales (*Balaena mysticetus*)^4^. The evolutionary relationships between sociality and longevity in mammals are complex^5^ yet important for understanding evolutionary strategies, i.e., life history diversity across organisms. In mammals, most of the evidence for links between sociality and longevity comes from single species. For example, affiliative social bonds, which are pervasive among group-living species, can extend a species’ lifespan by decreasing mortality and enhancing health and survival outcomes. In humans, strong social relationships can reduce the risk of physiological dysregulation^6^. With respect to other mammals, female chacma baboons (*Papio ursinus*) with strong and stable social bonds live longer than those with weak connections^7,8^; similar results have been reported in rhesus macaques (*Macaca mulatta*)^9^. Conversely, a negative correlation between affiliative relationships and longevity has been reported in female yellow-bellied marmots (*Marmota flaviventer*)^10^. Even though a small number of cross-species studies have tested the association between sociality and aging or longevity, they primarily focused on eusocial species^11–13^ and cooperatively breeding species^14,15^. Therefore, it remains unclear whether associations between longevity and other types of social organization are a common feature across the mammalian phylogeny.

In addition, the molecular mechanisms underlying the evolutionary association between social organization and longevity are not fully understood. Previous studies have suggested some possible processes, e.g., stress reduction, parasite infections, and pace of life (fast-slow continuum)^16^. According to the stress-buffering hypothesis, strong social bonds or social support can reduce adverse environmental stimuli or stress and enhance health and longevity in humans^17^. Social organization can also influence the spread of parasites in the population. For example, group-living species are vulnerable to infectious diseases because of the high social contact rates and close social interactions among individuals, but social species may have evolved a strong immune defense to minimize disease risk and protect themselves against pathogens^18^. One more possible link between social organization and longevity is the pace of life, which reflects an organism’s strategic allocation of resources between survival and reproduction. Species with a fast life history are characterized by rapid development, high reproductive rates, and short lifespan, whereas species with a slow life history are characterized by slow development, low reproductive rates, and a long lifespan^19^. Given that sociality and fitness are positively associated in some mammals^20–22^ and social bonds require major time investments before they yield survival benefits, social bonds are expected to have evolved in species with a slow life history and a longer lifespan^23^. In summary, we are only beginning to understand the evolution and the molecular mechanisms underlying the diversity of social organizations^24–28^ and longevity^29,30^.

In this work, we compare models with different evolutionary conjectures between social organization (i.e., solitary, pair-living, and group-living)^31^ and longevity across ~1000 mammals using a Bayesian framework. Moreover, we conduct a comparative brain transcriptomic analysis of 94 mammals to detect candidate genes and pathways associated with social organization and longevity, after controlling for body mass, ecological factors, life history traits, and phylogenetic relationships. We show that group-living species lived longer than solitary species and identify 31 genes, hormones, and immunity-related pathways involved in the correlated evolution of social organization and longevity.

## Results

### Evolutionary pathways for social organization and longevity

**Fig. 1:**
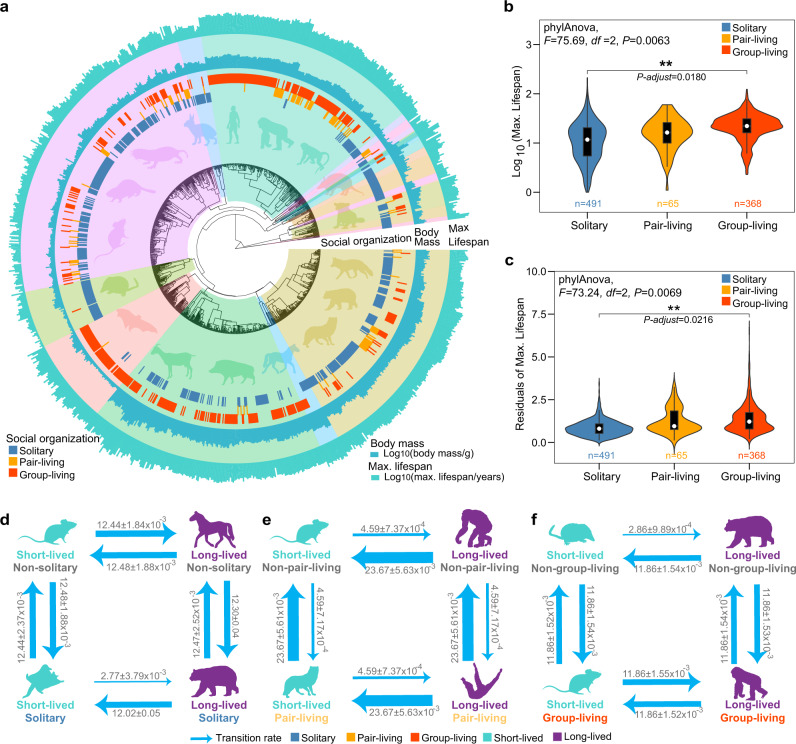
Evolutionary analyses of social organization and longevity in 974 mammalian species. **a** Phylogenetic distribution of social organization, adult body mass, and longevity (*n* = 974). The inner circle represents a species’ social organization: solitary (blue), pair-living (orange), and group-living (red). The middle layer indicates the absolute adult body mass (g) and the outer layer indicates the longevity (years); both variables were log_10_ transformed. Colors of shadings distinguish different mammals’ orders. **b** Difference in absolute longevity and **c** relative longevity (residuals of longevity, which was adjusted for body mass) across the three social organization states (solitary: *n* = 491, pair-living: *n* = 65, group-living: *n* = 368). We accounted for the effects of phylogenetic non-independence among species using a Phylogenetic ANOVA. Two-sided and Hommel method adjust *P* values are reported. The white dot represents the median in two violin plots, and the black box represents interquartile ranges (IQRs), i.e., the 25th and 75th percentiles. The whiskers extend up to the largest value within 1.5-fold IQR. Species numbers (*n*) are indicated in each social organization, respectively. Correlated evolution analysis for absolute short-lived (cyan) or long-lived state (purple): **d** non-solitary or solitary (blue); **e** non-pair-living or pair-living (orange); **f** non-group-living or group-living (red). **d** and **f** demonstrate correlated evolution. The number of species used in the analyses was *n* = 974. Arrows depict the likelihood of a transition between states, and their thickness corresponds to the magnitude of the various rates. Numbers indicate the transition rate across ten independent runs, and data are presented as mean ± SD. Silhouette images of animals are from PhyloPic database [http://phylopic.org/]. Source data are provided as a Source data file.

To assess the evolutionary transitions among social states and evolutionary pathways for longevity, we collected data on the social organization for as many extant mammalian species as possible through a comprehensive literature survey. We assigned 974 species into three types of social organization: solitary (*n* = 497), pair-living (*n* = 115), and group-living (*n* = 412). Fifty species had more than one state (Fig. 1a, details of classification in “Methods”). Data on body mass and longevity (defined by the maximum lifespan of a given species) for these species were also collected (Supplementary Table 1 and Supplementary Data 1). We first used phylogenetic comparative methods to calculate the phylogenetic signal of social organization and longevity. The maximum likelihood estimates of Pagel’s λ for the three social states was 0.94 when taking into account social polymorphism for a given species (phylogenetic signal test: *n* = 974, log_λ_ = −788.03, log_0_ = −1344.67, *P* < 0.001) and was 0.94 when using the uni-state species subset (*n* = 924, log_λ_ = −535.38, log_0_ = −938.78, *P* < 0.001). Pagel’s λ for longevity was 0.97 (*n* = 974, log_λ_ = 319.18, log_0_ = 1475.26, *P* < 0.001), illustrating that closely-related taxa generally have similar social organizations and longevity.

To determine the evolutionary pathways connecting the three states of social organization within mammals, we tested four models that allowed the transition rates between any of two states to vary differently. These four alternative models were: (a) the equal rates model (ER), in which all transition rates were the same; (b) the increasing complexity model (IC), which allowed transitions between solitary and pair-living, pair-living and group-living, but not solitary and group-living; (c) the all-rates-different model (ARD) or parameter-rich model^24^, in which all transition rates were different; and (d) the reversible-jump Markov chain Monte Carlo-derived model (RJ-MCMC), which is derived from the data using the reversible-jump procedure in Bayes Traits and has the highest posterior support^32^ (Supplementary Fig. 1a–d, Supplementary Table 2). Model comparisons showed that the ARD model was the best-supported model, which was significant against the RJ-MCMC model (Log BF = 9.24), the ER model (Log BF = 33.36), and the IC model (Log BF = 71.70) (Table 1). The ARD model showed that the transition rates varied across the three states of social organization (Supplementary Fig. 2a). For example, the transition rate from pair-living to solitary was 14 times higher than from solitary to pair-living (q_pair-living-solitary_ = 4.00 ± 1.55 × 10^−3^; q_solitary-pair-living_ = 0.29 ± 1.50 × 10^−4^), suggesting that the pair-living state was relatively unstable.

**Table 1:** Comparison of evolutionary models for social organization and longevity

Trait	Model	Rank	Parameters	Mean likelihood	Log BF
Social organization	ARD	1	6	−555.27	–
	RJ-MCMC	2	5	−559.89	9.24
	ER	3	1	−571.95	33.36
	IC	4	4	−591.12	71.70
Absolute lifespan	ARD	1	2	−346.26	–
	RJ-MCMC	1	2	−346.26	–
	ER	2	1	−361.43	30.34
Relative lifespan	ARD	1	2	−329.61	–
	RJ-MCMC	1	2	−329.61	–
	ER	2	1	−339.16	19.10

*n* = 974. Log BF = 2 × (log marginal likelihood complex model – log marginal likelihood simple model). The simple model is favored when Log BF < 2, while there is positive evidence to support the complex model when Log BF > 2 (strong evidence, 5–10; very strong evidence, >10)^32^.

*ARD* all-rates-different model, *RJ-MCMC* reversible-jump Markov chain Monte Carlo-derived model, *ER* equal rates model, *IC* increasing complexity model.

We then reconstructed the evolutionary pathway of longevity by constructing three alternative models (i.e., ER model, ARD model, and RJ-MCMC model) (Supplementary Fig. 1e–g). Comparisons of these three models showed that the RJ-MCMC and ARD models were better supported, on the basis of Bayes factors (BF), than the ER model (Table 1 and Supplementary Table 3). The average transition rate from a long-lived state to a non-long-lived state (q_absolute_ = 2.09 ± 3.85 × 10^−4^; q_relative_ = 1.71 ± 6.23 × 10^−4^) was about four times greater than that from a non-long-lived to a long-lived state (q_absolute_ = 0.54 ± 1.42 × 10^−4^; q_relative_ = 0.53 ± 1.13 × 10^−4^) (Supplementary Fig. 2b, c).

### Correlated evolution of social organization and longevity

We conducted phylogenetic ANOVA analyses to estimate differences in longevity among the three states of social organization while controlling for phylogenetic non-independence among species. Longevity was significantly different between the solitary state and the group-living state, with group-living species showing higher longevity than solitary species (phyloAVOVA: *n*_multi-states_ = 974, *t* = 12.40, *P-adjust* = 0.04; *n*_uni-state_ = 924, *t* = 12.28, *P-adjust* = 0.02, Fig. 1b). Since longevity is correlated with adult body mass (Spearman’s rank test: *r* = 0.71, *P* < 2.20 × 10^−16^), we also measured relative longevity which was calculated using the body mass adjusted residuals with the equation from the AnAge database (“Methods”). Similar results were obtained for relative longevity (phyloAVOVA: *n*_multi-states_ = 974, *t* = 12.01, *P-adjust* = 4.80 × 10^−2^; *n*_uni-state_ = 924, *t* = 11.94, *P-adjust* = 0.02, Fig. 1c). In addition, we conducted MCMCglmm models to control for phylogeny, body mass and factors related to external mortality: activity (diurnal, nocturnal or others), lifestyle (terrestrial, aerial, arboreal, semi-arboreal, freshwater, marine, or terrestrial-marine), and fossoriality (non-fossorial or subterranean). The results consistently showed that pair-living or/and group-living species lived longer than solitary species when using multi-states of the social organization dataset (MCMCglmm: *n*_multi-states_ = 947, pair-living vs. solitary, post mean = 0.10, pMCMC = 1.11 × 10^−3^; group-living vs. solitary, post mean = 0.06, pMCMC < 6.00 × 10^−4^; pair-living and group-living, post mean = 0.06, pMCMC = 0.03) and uni-state of the social organization dataset (*n*_uni-state_ = 897, pair-living vs. solitary, post mean = 0.10, pMCMC < 6.00 × 10^−4^; group-living vs. solitary, post mean = 0.06, pMCMC = 1.11 × 10^−3^). The results of activity, lifestyle and fossoriality are shown in Supplementary Table 4.

To evaluate whether changes in longevity depended on social organization, we compared the independent and dependent RJ-MCMC models of three combinations of variables: non-solitary/solitary and absolute short-lived/long-lived (>26 years), non-pair-living/pair-living and absolute short-lived/long-lived as well as non-group-living/group-living and absolute short-lived/long-lived. The results favored the dependent model for both solitary (Log BF = 3.18, Table 2 and Supplementary Table 5) and group-living (Log BF = 9.58, Table 2 and Supplementary Table 6), suggesting the existence of correlated evolution between social organization and longevity across the mammalian phylogeny. We also considered the effect of taxonomic sampling and the different classifications of long-lived species on the correlated evolution analyses. Random taxon sampling (i.e., randomly selecting 50 to 95% of the total number of species at a 5% interval) and repeated comparisons of independent and dependent RJ-MCMC models provided further support for the correlated evolution of solitary living and longevity (>26 years, 51% of model comparisons), as well as group-living and longevity (>26 years, 63% of model comparisons, Supplementary Fig. 3a). Consistent with these findings, random taxon sampling and model comparisons with two different cut-offs for long-lived species (>17 or >35 years), suggested the correlation between social organization and longevity. There was a correlation between solitary living and longevity when using the 17-year cut-off (79% of model comparisons) and 35-year cut-off (52% of model comparisons); and also a correlation between group living and longevity when using the 35-year cut-off (98% of model comparisons, Supplementary Fig. 3b, c). In addition, when taking the uncertainty of phylogenetic relationships into account and using a different phylogenetic tree^33^, the correlated evolution between solitary and longevity was supported by the analyses with the 26-year and the 17-year cut-off; the correlated evolution between group-living and longevity was supported by the analyses with the 17-year and 35-year cut off (*n*_multi-states_ = 969; Supplementary Table 7).

**Table 2:** Likelihoods of dependent and independent models estimated for the correlated evolution of social organization and longevity

Social states	Longevity states	Mean likelihood of model		Log BF	Correlated evolution
(no/yes)	(no/yes)	Dependent	Independent		
Solitary	Absolute long-lived	−795.02	−796.61	3.18	Yes
Pair living	Absolute long-lived	−643.81	−637.58	−12.46	No
Group living	Absolute long-lived	−775.78	−780.57	9.58	Yes
Solitary	Relative long-lived	−768.18	−777.02	17.68	Yes
Pair living	Relative long-lived	−627.74	−622.30	−10.88	No
Group living	Relative long-lived	−756.35	−760.49	8.28	Yes

*n* = 974. Absolute long-lived species: longevity >26 years. Relative long-lived species: the residual of longevity >1.38. The residual of longevity for each species was calculated using the body mass adjusted residuals using the equation from the AnAge.

We then attempted to determine whether transitions to a long-lived state were more likely in group-living than solitary species. Model estimation revealed that the transition rate from a short-lived state to a long-lived state was higher for non-solitary than solitary species (q_non-solitary_ = 12.44 ± 1.84 × 10^−3^; q_solitary_ = 2.77 ± 3.79 × 10^−3^), and higher for group-living than non-group-living species (q_groupliving_ = 11.86 ± 1.55 × 10^−3^; q_non-group-living_ = 2.86 ± 9.89 × 10^−4^; Fig. 1d–f). This result is consistent with the prediction that group-living species are more likely to be long-lived. We then tested if transitions to a group-living state were different for long-lived and short-lived species; we found that the transition rate from a solitary to a non-solitary state was the same in long-lived species and short-lived species (q_long-lived_ = 12.47 ± 2.52 × 10^−3^; q_short-lived_ = 12.44 ± 2.37 × 10^−3^; Fig. 1d). The transition rate from a non-group-living state to a group-living state was also the same in long-lived species and short-lived species (q_long-lived_ = 11.86 ± 1.53 × 10^−3^; q_short-lived_ = 11.86 ± 1.54 × 10^−3^; Fig. 1f), suggesting that longer longevity does not promote the formation of group-living. In addition, the correlated evolution of social organization and longevity was also supported when body mass was taken into account (Table 2; residuals of longevity > 1.38, solitary: Log BF = 17.68; group-living: Log BF = 8.28, Supplementary Fig. 4a–c). The random taxon sampling of different classifications of relative long-lived species further supported the correlated evolution between solitary living and longevity (residuals of longevity >1.38, 92% of model comparisons; residuals of longevity >1.83, 98% of model comparisons; Supplementary Fig. 5a–c), and the correlated evolution between group living and longevity (residuals of longevity >1.38, 55% of model comparisons; residuals of longevity >1.83, 95% of model comparisons; Supplementary Fig. 5a–c). Similarly, when a different phylogenetic tree and different cut-offs of the residuals of longevity were used, the correlated evolution between social organization and relative longevity was also supported (*n*_multi-states_ = 969; Supplementary Table 7).

In addition, to investigate whether longevity favors any social organization transformation, we compared the independent and dependent RJ-MCMC models using species with a uni-state of social organization. The results supported that social organization transformation favors longer life during solitary transit to the group-living state rather than from solitary transit to pair-living, or from pair-living transit to the group-living state (Supplementary Table 8). The transition rate from short-lived to long-lived species was higher in group-living than solitary species (absolute longevity: q_solitary_ = 2.98 ± 6.97 × 10^−3^; q_group-living_ = 10.45 ± 5.29 × 10^−3^; relative longevity: q_solitary_ = 3.37 ± 8.70 × 10^−3^; q_group-living_ = 10.30 ± 9.51 × 10^−3^, Supplementary Fig. 6a–f).

### Gene expression of social organization and longevity

**Fig. 2:**
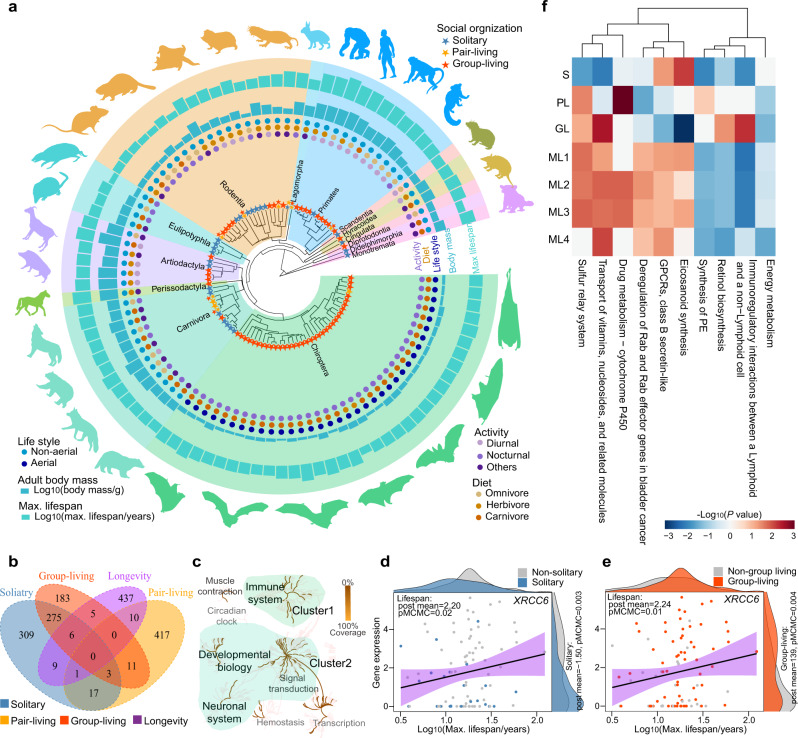
Genes and pathways whose expression was correlated with social organization and longevity in 94 mammalian species. **a** Species (*n* = 94) with RNA-seq and six life history traits (social organization, activity, diet, lifestyle, adult body mass, and longevity) used in the MCMCglmm analyses. Colorful shadings display different mammals’ orders. Silhouette images of animals are from PhyloPic database [http://phylopic.org/]. **b** Venn diagram showing the number of significant overlapping genes across solitary, pair-living, group-living, and longevity. **c** Clusters according to the function of 31 genes that were significantly associated with social organization and longevity. Each node represents a pathway from the Reactome database^34, 35^; pathways in which significant genes were involved are colored brown. Light green shading represents two clusters of gene function. **d**, **e** Example of a significant gene (*XRCC6*) that was downregulated in solitary species, upregulated in group-living species and also positively correlated with lifespan; the regression lines were generated from the linear regression model. Purple range display 95% confidence interval around the smooth line. Coefficients (post mean) and *P*-values (pMCMC) from the MCMCglmm analyses are also shown. The number of species used in the MCMCglmm was *n* = 94. **f** A heat map showing pathways that were significantly associated with social organization and longevity. S: solitary; PL: pair-living; GL: group-living; ML1–ML4: longevity in model 1 to model 4 (see “Methods”). Color code for social organization and longevity: blue = solitary; orange = pair-living; red = group-living; purple = lifespan. Source data are provided as a Source data file.

To identify genes that could underpin the correlated evolution of social organization and longevity, we generated brain transcriptomics of 94 mammals belonging to 14 orders, 39 families, and 67 genera (Fig. 2a, Supplementary Data 2, “Methods”). Specifically, 57% of species and 62% of samples (166 samples of 54 species) were newly collected in this study. The sampled species were assigned to three states of social organization (solitary, *n* = 26; pair-living, *n* = 11; group-living, *n* = 65); eight species had more than one state. The sampled species also covered a longevity range from 3.2 years in Chinese mole shrew (*Anourosorex squamipes*) to 122.5 years in *Homo sapiens* (Fig. 2a, Supplementary Data 3, and Supplementary Table 9). Using the human coding sequences as a reference, we employed a reciprocal-blast approach to identify the orthologous gene set. The orthologous genes that were shared by >70% of the total number of species (i.e., 66 of 94 species) were selected for subsequent analyses (“Methods”). Finally, gene expression for 13,402 orthologous genes was measured across all brain samples. We then used MCMCglmm models to identify genes whose expression significantly correlated with any of the social organization states; these models also controlled for phylogenetic relationships and other confounding factors, including adult body mass, activity (nocturnal, diurnal, and other), diet (carnivore, herbivore, and omnivore), and lifestyle (non-aerial and aerial). Hundreds of genes were significantly associated with solitary living (up: 366 genes, down: 254 genes), pair-living (up: 393 genes, down: 66 genes), and group-living (up: 162 genes, down: 321 genes) (Supplementary Fig. 7a–f, Supplementary Data 4). There were three overlapping genes among the three states of social organization: *ATP1A2*, *ALDH1L2*, and *WDFY1*. We also detected genes that were shared by two states (solitary-pair-living: 21 genes; solitary-group-living: 284 genes; pair-living-group-living: 14 genes, Supplementary Fig. 7f). We detected 262 genes whose expression was significantly correlated with longevity; this was supported by all four different models in the MCMCglmm analyses (“Methods”, Supplementary Fig. 8, Supplementary Data 5; see Supplementary Data 6 for the results of each model).

In total, we found 31 genes whose expression levels were significantly associated with both social organizations and longevity (Fig. 2b, Supplementary Data 7). The pathway topology analysis of these genes using the Reactome platform^34,35^ revealed two strong clusters. The first cluster included immune-related genes (Fig. 2c). Nine genes (i.e., *UBL7*, *TNNT3*, *XRCC6*, *ATP2A2*, *NPHS1*, *KALRN*, *C1QC*, *MCL1*, and *ZFP36*) that are involved in the innate immune response^36^. Gene *C1QC* (MCMCglmm: solitary, post mean = 1.16, pMCMC = 0.04; longevity, post mean = −2.33 ± 0.05, pMCMC = 0.03 ± 7.17 × 10^−3^) participates in encoding a complex heterotrimer C1q that plays a vital recognition role in the complement pathway. C1q has diverse biological functions, including complement activation, innate immune defense, cellular regulation, reproduction, development and neurodegenerative disorders^37,38^. The well-known immune gene *ZFP36* (MCMCglmm: solitary, post mean = 1.07, pMCMC = 0.02; longevity, post mean = 2.21 ± 0.07, pMCMC = 0.02 ± 5.05 × 10^−3^) modulates anti-viral immunity by controlling T cell activation^39^ and protects against inflammatory diseases through regulating inflammatory cytokines, such as TNF-α^40,41^. *ZFP36* also plays a role in neuroprotection and inhibits neuronal apoptosis^42^. Another gene of interest was *XRCC6* (MCMCglmm: solitary, post mean = −1.50, pMCMC = 3.33 × 10^−3^, Fig. 2d; group-living, post mean = 1.39, pMCMC = 4.44 × 10^−3^, Fig. 2e; longevity, post mean = 2.27 ± 0.05, pMCMC = 0.01 ± 4.37 × 10^−3^), which encodes subunit p70 of the p70/p80 autoantigen. A recent study has shown that a splicing variant in *XRCC6* may cause autism, a disorder that causes significant social, communication, and behavioral challenges^43^. Knockout of *XRCC6* decreases lifespan in mice^44^ and the high expression of *XRCC6* leads to a longer average lifespan in humans^45^. Thus, this gene likely plays a role in both longevity and social organization.

The second cluster of genes whose expression was correlated with longevity and social organization consisted of genes involved in the regulation of hormones, neural systems, and signal transduction (Fig. 2c), e.g., *MTM1*, *SLC29A2*, *ATP2A2, KALRN, RHOBTB2*, *SLC6A19*, and *MCL1*. Some of these genes are suggested to play a role in social behavior. For instance, the *KALRN* gene (MCMCglmm: group-living, post mean = −1.16, pMCMC = 0.02; longevity, post mean = −2.40 ± 0.11, pMCMC = 0.02 ± 6.93 × 10^−3^) produces several alternatively spliced forms of kalirin, which is essential for synaptic connections, spine development, cognition, learning, fear conditioning and social behavior^46^. Knockout of this gene in mice caused working memory deficits, locomotor hyperactivity and reduced social behavior^47,48^. Gene *SLC29A2* (MCMCglmm: pair-living, post mean = −1.65, pMCMC = 0.02; longevity, post mean = 2.30 ± 0.05, pMCMC = 0.04 ± 3.18 × 10^−3^) is linked to the development of depression. Knockout of the *ATP2A2* (MCMCglmm: pair-living, post mean = −1.60, pMCMC = 0.02; longevity, post mean = 2.42 ± 0.21, pMCMC = 0.03 ± 0.02) impaired fear memory and changed behaviors in novel environments^49^. Nonetheless, the contribution of these genes to longevity is currently unknown and worthy of further exploration.

To gain an overall view of gene expression related to social organization and longevity, we employed a modified summary statistic approach (i.e., the polysel method, “Methods”). This approach identifies pathways that show accumulated correlation rather than outlier genes^50,51^. The sum of the posterior means (generated from MCMCglmm models) of the genes in each pathway was calculated as the SUMSTAT score and compared to a null distribution of random gene sets. We found 56, 56, and 45 pathways showing significant correlations with solitary, pair-living, and group-living species compared with non-solitary, non-pair-living, and non-group-living species, respectively (Supplementary Fig. 9, Supplementary Data 8). We also identified 14 longevity-associated pathways that occurred in four models (Supplementary Fig. 10, Supplementary Data 9; see Supplementary Fig. 11 and Supplementary Data 10 for the results of each model). A total of 10 pathways showed accumulated correlations with both social organization and longevity (Fig. 2f, Supplementary Data 11). Among them, the hormones-related pathway “G-protein-coupled receptors (GPCRs), class B secretin-like” was positively associated with both solitary living and longevity, but negatively associated with group living (polysel: solitary, score = 6.18, *P* = 4.71 × 10^−2^; group-living, score = −6.04, *P* = 3.96 × 10^−2^; longevity, score = 7.15, *P* = 3.83 × 10^−2^). The secretin-like family of GPCRs include receptors for polypeptide hormones, such as secretin, parathyroid hormone and vasoactive intestinal peptide, which play vital roles in physiological homeostasis, nervous diseases, the stress response and longevity^52–54^. Acting as a catalyst in steroid hormone synthesis^55^, the cytochrome P450-related pathway has been enriched (“drug metabolism - cytochrome P450”, polysel: pair-living, score = 18.52, *P* = 1.40 × 10^−4^; longevity, score = 16.00 ± 0.04, *P* = 0.02 ± 1.06 × 10^−3^). The mutation of cytochrome P450 has been shown to increase longevity in *Caenorhabditis elegans*^56,57^. In addition, cytochromes P450 regulate inflammation and infection^58^ and the generation of eicosanoids (the “eicosanoid synthesis” pathway, polysel: solitary, score = 7.94, *P* = 7.71 × 10^−3^; group-living, score = −9.07, *P* = 7.77 × 10^−4^; longevity, score = 8.11, *P* = 4.68 × 10^−2^). Eicosanoids have a broad range of functions, including reproduction, physiological homeostasis, and cell growth regulation; in particular, they play a role in regulating immune response and inflammatory processes in various diseases^59–62^. Another immunity-related pathway “immunoregulatory interactions between a lymphoid and a non-lymphoid cell” was negatively correlated with longevity (polysel: longevity, score = −24.20 ± 0.28, *P* = 7.78 × 10^−3^ ± 1.24 × 10^−3^). Interestingly, this pathway is downregulated in solitary species, but upregulated in group-living species (solitary, score = −11.19, *P* = 0.01; group-living, score = 11.99, *P* = 5.52 × 10^−3^), and may be an immune response to elevated pathogen transmission among hosts and infectious disease risks in a gregarious setting. Taken together, both the function annotation of overlapping genes and the gene set enrichment analysis of all genes identified the hormones and immunity processes underlying the association between social organization and longevity.

### Selection features of social organization and longevity

**Fig. 3:**
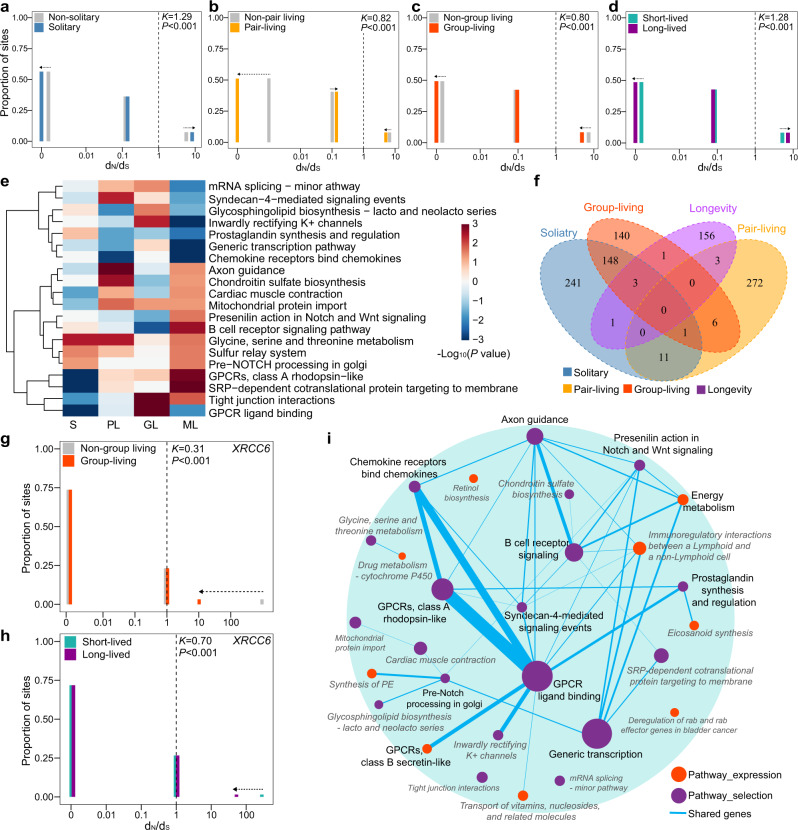
Cross-talk between expression and selection in social organization and longevity. The pattern of selection characterizing social organization identified with a RELAX analysis (species: *n* = 94): **a** solitary species, *P* = 1.95 × 10^−7^, **b** pair-living species, *P* = 5.89 × 10^−6^, **c** group-living species, *P* = 5.81 × 10^−7^, and **d** longevity, *P* = 6.76 × 10^−7^. *P* value was calculated using Likelihood-ratio test (LRT). *K* < 1 indicates relaxed selection and *K* > 1 indicates intensified selection. Genes were under purifying selection when *d*_N_/*d*_S_ < 1 and positive selection when *d*_N_/*d*_S_ > 1. Arrows represent the *d*irection of change in *d*_N_/*d*_S_. The median value of the proportion of sites is shown with a bar plot. Social organization and longevity are colored as follows: solitary: blue; pair-living: orange; group-living: red; short-lived state: cyan; and long-lived state: purple. **e** A heat map of significant pathways that ov**e**rlapped between social organization and longevity. S: solitary; PL: pair-living; GL: group-living; ML: maximum lifespan. **f** A Venn diagram showing the number of genes with changes in expression levels and selection among solitary, pair-living, group-living, and longevity. Examples of genes that were associated with expression and selection in both social organization and longevity: **g** gene *XRCC6* was under relaxed selection in group-living species (*P* < 1.00 × 10^−17^) and **h** long-lived species (*P* = 1.06 × 10^−5^). *P* value was calculated using Likelihood-ratio test (LRT). The interpretation of *K* and *d*_N_/*d*_S_ is the same as (**a**–**d**). The number of species used in RELAX analyses was *n* = 94. **i** Network of pathways showing correlations with expressio*n* (red round) and selection features (purple round) in both social organization and longevity. The circle size represents the number of genes in this pathway. The thickness of connective lines displays the number of shared genes between two pathways. Source data are provided as a Source data file.

Whether social organizations or longer lifespans are under selection remains controversial^63,64^. To characterize the selection features of social organization and longevity, we used RELAX^65^ to estimate the selection coefficients (*K*) for orthologous genes under different states of social organization (solitary, pair-living, and group-living) and longevity (long-lived vs. short-lived) (Fig. 3a–d, Supplementary Data 12, Supplementary Data 13). In solitary branches, genes mostly experienced intensified selection (5448 genes, *K* > 1, likelihood ratio test (LRT) *P* < 0.05) rather than relaxed selection (3200 genes, *K* < 1, LRT *P* < 0.05, Fig. 3a). We identified 3747 genes that showed evidence of intensified selection and 4589 genes that showed evidence of relaxed selection in the pair-living state (Fig. 3b). A larger number of genes associated with group-living experienced relaxed selection (5570 genes, *K* < 1, LRT *P* < 0.05) than intensified selection (3170 genes, *K* > 1, LRT *P* < 0.05, Fig. 3c). Longevity appeared to be under intensified selection (Fig. 3d), as more genes were subjected to intensified selection (5364 genes, *K* > 1, LRT *P* < 0.05) than relaxed selection (3564 genes, *K* < 1, LRT *P* < 0.05) in the long-lived state. Moreover, a larger number of genes that experienced intensified selection for longevity were found under intensified selection in solitary rather than group-living species (Pearson’s chi-squared test: *χ*^2^ = 527.96, *df* = 1, *P* < 0.001). By contrast, a greater number of genes under relaxed selection in the long-lived state also experienced more relaxed selection in group-living species than solitary species (Pearson’s chi-squared test: *χ*^2^ = 430.42, *df* = 1, *P* < 0.001). These results suggest that the long-lived state in group-living mammals involves relaxation selection.

We then employed the same pathway enrichment approach as described above (polysel method) using the selection coefficient (*K*) of each gene as the statistic. For the trait social organization, we identified 132 pathways under significant intensified or relaxed selection (solitary: 50 pathways; pair-living: 56 pathways; group-living: 53 pathways; *P* < 0.05, Supplementary Data 14; Supplementary Fig. 12a). We detected more intensified pathways than relaxed pathways in solitary species (34 vs. 16) and pair-living (36 vs. 20), but fewer intensified than relaxed pathways in group-living species (21 vs. 32). One pathway was shared among all three states of social organization (“Spliceosome, U2-snRNP” pathway), and a few pathways were also identified in two states (i.e., solitary-pair-living: 2; solitary-group-living: 12; pair-living-group-living: 4). For long-lived species, we also detected signatures of intensified selection in 40 pathways and relaxed selection in 23 pathways (Supplementary Fig. 12b, Supplementary Data 15). In total, 20 overlapping pathways were detected under selection for both social organization and longevity (Fig. 3e, Supplementary Data 16); however, most of these pathways did not show an identical trend in the selection force. For example, “B cell receptor signaling pathway” showed accumulated relaxed selection in group-living species, but intensified selection in long-lived species (polysel: group-living, score = −21.03, *P* = 3.04 × 10^−3^; longevity, score = 36.61, *P* = 2.42 × 10^−3^); “glycosphingolipid biosynthesis-lacto and neolacto series” experienced accumulated intensified selection in group-living species, but relaxed selection in long-lived species (polysel: group-living, score = 5.37, *P* = 0.03; longevity, score = −3.07, *P* = 0.04). These findings suggest that even though common pathways can be utilized by natural selection for longevity and social organization, the underlying molecular mechanisms and regulatory approaches are different.

### Cross-talk between gene expression and selection

We further discovered eight genes with changes in their expression levels and selection for both social organization (Supplementary Fig. 12c–e) and longevity (Supplementary Fig. 12f), i.e., *SHKBP1*, *MTM1*, *XRCC6*, *UBL7*, *VWA5A*, *PUS3*, *MCL1*, and *COX7A1*) (Fig. 3f, Supplementary Data 17). In particular, gene *XRCC6* was not only identified in the gene expression analyses (see above), but also experienced selection in solitary, group-living and long-lived species (RELAX: solitary, *K* = 2.58, *P* = 4.34 × 10^−13^; group-living, *K* = 0.31, *P* = 1.00 × 10^−17^, Fig. 3g; longevity, *K* = 0.70, *P* = 1.06 × 10^−5^, Fig. 3h). *MTM1* was upregulated in both solitary and long-lived species (MCMCglmm: solitary, post mean = 1.38, pMCMC = 0.03; longevity, post mean = 2.55 ± 0.21, pMCMC = 0.03 ± 0.01). This gene was also under intensified selection in solitary, but relaxed selection in long-lived species (RELAX: solitary, *K* = 2.52, *P* = 1.00 × 10^−17^; longevity, *K* = 0.52, *P* = 2.05 × 10^−7^). Loss-of-function of *MTM1* leads to a genetic neuromuscular disorder, X-linked centronuclear myopathy^66^, as evidenced by a decreased lifespan in knockout mice^67^. Another gene, *MCL1*, was upregulated (MCMCglmm: pair-living, post mean = 1.31, pMCMC = 0.04) and under relaxed selection in pair-living species (RELAX: *K* = 0.54, *P* = 2.97 × 10^−5^). In addition, *MCL1* was negatively associated with longevity (MCMCglmm: post mean = −2.33 ± 0.13, pMCMC = 0.04 ± 8.48 × 10^−3^) and experienced intensified selection in long-lived species (RELAX: *K* = 2.28, *P* = 4.55 × 10^−6^). As a notable member of the anti-apoptotic Bcl-2 family, *MCL1* can regulate cell cycle, cell proliferation, and DNA damage repair, which may contribute to longevity^68^. *MCL1* is critical for neuronal development^69^, where the loss of *MCL1* leads to apoptosis of neuronal progenitors^70^.

One pathway was correlated with the expression and selection features for both social organization and longevity: “sulfur relay system” (polysel: expression in solitary, score = −4.17, *P* = 0.02, in pair-living, score = 5.37, *P* = 0.03 and in longevity, score = 6.81 ± 0.20, *P* = 0.02 ± 3.41 × 10^−3^; selection in solitary, score = 3.40, *P* = 0.03 and in longevity, score = 4.26, *P* = 0.03). The sulfur relay systems are involved in the complex process of trafficking and delivery of sulfur, which is an essential element for living organisms and a component of major biomolecules^71,72^. For example, sulfur-containing nucleosides in tRNA molecules have diverse functions, including stabilization of tRNA structure, proper codon-anticodon base pairing, and insurance of accurate and efficient translation^73,74^. Sulfur-containing modification at tRNA position 34 has revealed a biological role of sulfur in growth, oxidative stress, and metabolic cycles in yeast^73^. The lack of this modification can cause myoclonic epilepsy with ragged-red fibers (MERRF), which clinically manifests as cerebellar ataxia in humans^75^. Besides, only one pathway whose expression was associated with both social organization and longevity was under significant selection in social organization, but not longevity: “GPCRs, class B secretin-like.” In addition, five pathways that were under selection for both social organization and longevity also showed significant changes of expression levels in social organization (i.e., “tight junction interactions,” “axon guidance”) or longevity (i.e., “SRP-dependent cotranslational protein targeting to membrane,” “mitochondrial protein import,” “GPCRs, class A rhodopsin-like”). Among them, “GPCRs, class A rhodopsin-like” (polysel: expression in longevity, score = 42.45 ± 2.02, *P* = 4.86 × 10^−3^ ± 1.25 × 10^−3^; selection in solitary, score = −37.57, *P* = 2.50 × 10^−6^ and in longevity, score = 29.16, *P* = 1.27 × 10^−3^) is the largest group of GPCRs, representing members such as light, hormones and neurotransmitter receptors^76,77^. These receptors are associated with regulation of neuroendocrine function, sleep-wake cycle, energy metabolism, feeding, anxiety, and stress responses^78,79^. Since mutations in class A GPCRs can lead to a large number of diseases, including depressive disorders, schizophrenia, and bipolar disorder, they also serve as drug targets in humans^80,81^. Another pathway, “axon guidance” (polysel: selection in pair-living, score = 33.48, *P* = 6.17 × 10^−4^ and in longevity, score = 22.66, *P* = 0.04), is key to brain development and neural circuit formation^82^. In addition, the “tight junction interactions” pathway (polysel: selection in solitary, score = −7.03, *P* = 0.02; in group-living, score = 9.27, *P* = 1.25 × 10^−4^ and in longevity, score = 8.20, *P* = 0.01) regulates cell-cell communication and cellular growth, development, differentiation, and pathogen infection^83^. Dysregulation of tight junctions not only increases the entry and spread of viruses or bacteria^84^, but also affects age-related neurodegenerative disorders^85^. Similarly, “SRP-dependent cotranslational protein targeting to membrane” (polysel: expression in longevity, score = 38.39, *P* = 0.02; selection in solitary, score = −26.09, *P* = 2.45 × 10^−4^ and in longevity, score = 37.12, *P* = 2.50 × 10^−6^) regulates viral infections^86^, but its function in longevity remains unclear. Very few pathways were shared by social organization and longevity in the gene expression and selection analyses. This finding points to a fine-tuned network (e.g., Fig. 3i), which involves beneficial mutations and changes of expression in different, but functionally connected genes or pathways, and is a favored approach to maintain the plasticity and stable evolution of social organization and longevity.

## Discussion

In this study, we provide evidence for the correlated evolution of social organization and longevity across the mammalian phylogeny and show that group-living species lived longer than solitary species. There was no significant difference in longevity between pair-living and group-living species, or between pair-living and solitary species, suggesting that pair-living alone is unable to mediate lifespan extension despite it can generate an association between a pair of individuals. Long lifespan favored by group-living species may be because group living reduces extrinsic mortality by limiting the risks of predation and starvation, and the strong and stable social bonds formed among group members have the power to enhance longevity^8,9,87^. These benefits are expected to override the costs inherent in group living, such as competition for mating partners and food, stress from higher-ranking individuals, and the spread of infectious diseases via social contacts^88^. Another explanation for the correlated evolution is that kin selection may be a driver of longevity^89^. Group living leads to the co-residence of males or females and sex-specific philopatry. Preferential association among kin can influence coalition formation^90^, cooperative breeding^91^, parallel dispersal^92^, and the establishment of social hierarchies^93^, which further enhance individual health or offspring survival^7,93,94^ and ultimately increase an individual’s or their relatives’ evolutionary fitness^95^. The length of a lifespan may be affected by inclusive fitness benefits. For example, to maximize the rate of offspring survival, the lifespan of parents or grandparents may be extended to allow for the provision of parental care or even grandparental care to offspring^16,96^.

The transcriptomic features associated with the correlated evolution of social organization and longevity indicated that hormonal regulation and immunity constitute the mechanistic foundation for the association between social organization and longevity. Peptide hormones (e.g., growth hormone, insulin-like growth factor-1, and insulin) have also been shown to perform crucial roles in aging and longevity. For example, defects in growth hormone production extend longevity in Snell dwarf mice^97^. Reduced insulin-like growth factor-1 signaling has been shown to increase lifespan in mice, fruit flies, yeast, and worms^98,99^. Other types of hormone, steroids (e.g., testosterone, estradiol, and progesterone), control a range of social behaviors, including copulatory behavior, aggression, grooming behavior, and paternal behavior^100,101^. Specifically, neuroactive steroids produced in the nervous system and their receptors also play a role in the regulation of learning capacity, memory, decision-making, and depression^102^. A number of studies have demonstrated that steroid hormones also regulate lifespan; for example, the inhibition of sulfatase increases lifespan in *C. elegans*^103^.

The immunity or inflammation pathways and genes identified in this study support the view that immunity is instrumental to the correlated evolution of sociality and longevity^104^. Social organizations can affect immune responses. For example, in captive group-living long-tailed macaques (*Macaca fascicularis*), a higher rate of affiliation enhances an individual’s immune response^105^. In contrast, social isolation or a limited number of social ties can activate neuroendocrine regulation, accumulated inflammation burden, and impair immune function^106,107^. Moreover, several lines of evidence have demonstrated the effects of immunity and inflammation on social behavior, fitness, health and lifespan of social mammals. For example, interleukin-17a (IL-17a), a well-described mediator in inflammatory diseases, can rescue sociability deficits in offspring mice exposed to maternal immune activation by directly affecting their neuronal activity^108^. Immunity is also linked to reproductive behavior and thus indirectly affects the fitness of an individual^109^. A recent study has shown that male mice avoid mating when a female mouse is unhealthy^110^. Research has also shown that age-related changes in immunity, such as inflammatory markers interleukin-6 (IL-6) and TNF-alpha, increase with age in humans^111^ whereas the proportion of naive CD4 T cells in the blood declines with age in wild Soay sheep (*Ovis aries*)^112^.

We assumed that solitary species are generally less social than pair-living species, and both are less social than group-living species. However, mammal societies vary enormously in individual composition, size, patterns of parental care, cooperation, social relationships, and spatiotemporal dynamics of group members. Although some studies have provided conceptual frameworks and indices to quantify sociality or social complexity^31,113^, a consensus and more accurate measurements that could be used in large-scale comparative studies are needed. The accumulation of long-term field data on variables such as relatedness, affiliative relationships, social network cohesion, cooperation, and agonistic relationships among individuals will solidify our understanding of the evolutionary interplay between sociality and longevity. In summary, our study provides insights into the correlated evolution of social organization and longevity and serves as a basis for experimental validation and follow-up studies on the mechanistic drivers of this correlated evolution.

## Methods

### Data collection and compilation

All animal care and research protocols of this study were approved by the Institute of Zoology, Chinese Academy of Sciences (No. IOZ-IACUC-2021-129). We compiled data on various mammalian traits, including life history, social system, behavior, and habitat. These data were obtained from the literature^114–118^, reviews^25,26^, and databases, such as PanTHERIA^119^, PHYLACINE^120^, and AnAge^4^. The last search date was August 5, 2022. The sources of each data are listed in Supplementary Data 1, Supplementary Data 3 and Supplementary References. A complete dataset comprising data on adult body mass, maximum lifespan, and social organization, activity, lifestyle, fossoriality for 974 mammal species was used for subsequent analyses (Supplementary Data 1). To identify the candidate genes associated with social organization or longevity, we further generated brain transcriptomes for 94 mammal species. To control for possible effects of confounding variables on gene expression in the regression analyses, data on six traits (i.e., adult body mass, maximum lifespan, social organization, activity, diet, and lifestyle) for these 94 species were included in the models (Supplementary Data 3). Adult body mass and maximum lifespan were log_10_ transformed prior to analysis.

Moreover, we measured the adult body mass of 7 bat species in the wild for which no data were available in the existing literature and databases. We used imputation methods^121^ to estimate the maximum lifespan of 35 species that were missing from the database. In this estimation, adult body mass and female time to maturity were used since they are strongly correlated with maximum lifespan. The dataset included 1250 species with information on adult body mass and 816 species with information on adult body mass, female time to maturity, and maximum lifespan. In brief, we first used the finalfit package in R^122^ to evaluate the missing data pattern of the maximum lifespan (6% of NAs) and female time to maturity (30% of NAs) in the dataset with 1250 species: (a) missing completely at random (MCAR), assuming that data values do not relate to any other variables; (b) missing at random depending on adult body mass (MAR-BM), assuming that data values relate to adult body mass; and (c) MAR depending on phylogeny (MAR-Phy), assuming that data values relate to phylogeny. Then, to find the best imputation approach for our dataset, we employed three popular imputation methods: mice^123^, missForest^124^, and Phylopars^125^, following the process of Penone et al.^121^. The complete dataset with three traits for 816 species was set as the test dataset. We introduced missing data (5%, 10%, 20%, 25%, 30%, 40%, and 50%) in the test dataset and repeated the analyses at least ten times. Five approaches were then used to impute each missing dataset: mice with and without phylogeny, missForest with and without phylogeny, and Phylopars. With mice and missForest, we also obtained ten imputed datasets per imputation. The Phylopars approach used the phylogenetic trees directly, whereas the mice and missForest approaches used the phylogenetic eigenvectors from principal coordinate analysis in the R package PVR^126^. The normalized root mean squared error and Bias (see Penone et al.^121^ for equations) were calculated to compare the imputation methods^121^. Finally, we selected the best-performed method, Phylopars, to impute missing values in the dataset with 1250 species.

### Classification of social organization, longevity, and other traits

Social organization, i.e., the size, composition, and kinship structure of a basic social unit, varies across mammal species^31^. We used the criteria developed by Kappeler and van Schaik^1^ to classify the social organization of mammals into three states: solitary, pair-living and group-living. These definitions focus on adult males and adult females and do not consider subadults, juveniles, and infants. Solitary species were defined as individuals who live alone and rarely synchronize their general activity (e.g., forage, rest) and move with others other than for mating and raising their young. A species whose members spend the majority of their life in a solitary state but occasionally forage, migrate, travel, or rest in temporary groups, was categorized as solitary, e.g., *Cervus nippon*, *Enhydra lutris*, and some of *Balaena* spp.^114–116^. Species were classified as pair-living when an adult male and an adult female associate for more than one year within a common home range; this definition considers behavioral evidence of living together but not genetic evidence. Species were categorized as group-living if at least three adults live together, synchronizing their daily activities and interacting with each other^25^. Cooperatively breeding species were categorized as pair living if a group contains one breeding pair and their non-adult offspring; if there are more than two adults per group, they were classified as group living. We also considered multiple states of social organization for a particular species. We classified activity as one of three states: nocturnal, diurnal, and other (nocturnal/crepuscular, cathemeral, crepuscular, or diurnal/crepuscular) using the definitions and classification of Jones et al.^119^. Following the approach used by Kissling et al.^127^, we divided diet into three categories: carnivore, herbivore, or omnivore. In addition, we categorized lifestyle into terrestrial, aerial, arboreal, semi-arboreal, freshwater, marine, or terrestrial-marine, according to Faurby et al.^120^ and Thorley et al.^15^. The fossoriality of species included non-fossorial and subterranean^15^.

Longevity was defined by the maximum lifespan of a given species. This definition is widely used in comparative longevity studies^29,128,129^. The maximum lifespan of a species was referred to as absolute longevity. Given a strong positive correlation between adult body mass and maximum lifespan, we also measured “relative longevity” to control for the confounding effects of body mass. The “relative longevity”, or the residuals of longevity, were calculated using the body mass adjusted residuals with the equation from the AnAge database: residuals of longevity = maximum lifespan/(4.88 × adult body mass^0.153^)^29^. For analyses requiring categorical variables, mammals were divided into long-lived species and short-lived species. We used the 3^rd^ quartile of longevity (26 years) as the cut-off value for the long-lived state. This value is consistent with a previous study that labeled species whose maximum lifespan was more than 26 years as medium or long-lived^130^. Thus, we divided 974 mammalian species into absolute long-lived species (longevity > 26 years, *n* = 246) and absolute short-lived species (longevity ≤ 26 years, *n* = 728). To explore the effects of the classification of long-lived species on the phylogenetic comparative analyses, we also used two different cut-off values for longevity: 17 years (median of longevity) and 35 years (same range from median to the third quartile) (see section “Taxonomic sampling”). In addition, considering the influence of adult body mass on longevity, we used the 3^rd^ quartile of the residuals of longevity as an index to divide mammals into relative long-lived species (*n* = 244) and relative short-lived species (*n* = 730). The residuals of longevity were calculated using the equation from the AnAge database as described above. Mammals were classified as relatively long-lived species if the residuals of longevity were greater than the value of the 3^rd^ quartile (1.38), whereas species not meeting this criterion were classified as relatively short-lived species. Similarly, we also used two different cut-off values for relative longevity: 0.93 (median of the residuals of longevity) and 1.83 (same range from median to the third quartile). Long-lived species that were frequently identified in previous studies (e.g., *Balaena mysticetus*, *Heterocephalus glaber*, *Myotis brandtii*, *Homo sapiens*) were included as long-lived species, demonstrating the accuracy of the two classification approaches.

### Mammal phylogeny and phylogenetic signal

We used the mammalian phylogeny tree from TimeTree^131^ in our analyses. The name.check function of the package Geiger in R was used to check the concordance of species names between a trait dataset and a phylogenetic tree^132^. Extra tree tips were removed using the drop.tip function of the R package ape^133^. For the analyses that required binary trees, we randomly converted multifurcating trees to binary trees using the multi2di function of the R package ape. In addition, considering the uncertainty of phylogenetic relationships among species, we also re-analyzed data using a different phylogenetic tree from Upham et al.^33^.

We calculated Pagel’s lambda using the fitDiscrete function in the R package Geiger for categorical variables (social organization) and the phylosig function in the phytools for continuous variables (longevity). The phylogenetic signals of both absolute and relative longevity were calculated. There is no phylogenetic signal when Pagel’s λ = 0, whereas Pagel’s λ = 1 indicates that a strong phylogenetic signal is detected and the trait has evolved consistently with a Brownian motion model (i.e., that close relatives are more similar than expected).

### Evolutionary modeling of social organization and longevity

#### Evolutionary model of social organization

To evaluate the evolutionary pathway among solitary, pair-living and group-living, we assessed four alternative models in 974 mammalian species using a Bayesian framework, which was implemented in BayesTraits V3^134,135^. The transition rates between any of the two states of social organization were allowed to vary differently in these four alternative models. First, the equal rates model (ER) is the simplest model in which all transition rates were the same. That is, the transition rates were equal among solitary, pair-living and group-living (Supplementary Fig. 1). Second, the increasing complexity model (IC) posits that transitions are only permitted between solitary and pair-living, and pair-living and group-living, but not between solitary and group-living; transition rates can vary. Third, the all-rates-different model (ARD) dictates that all transition rates are different; this is also known as a parameter-rich model^24^. Fourth, the reversible-jump Markov chain Monte Carlo-derived model (RJ-MCMC) is derived from the data by the reversible-jump procedure in BayesTraits; this model structure is generated by the highest posterior support from the reversible-jump analysis (see details below).

To generate the best-supported RJ-MCMC model, we used the reversible-jump MCMC procedure with the hyper-prior approach, seeding the mean of the exponential prior from a uniform distribution on the interval of 0 to 2^24^. To prevent the rates from being too small to estimate, we scaled the branch lengths of the tree to have a mean of 0.01^32^. We ran each Markov chain Monte Carlo simulation for 100 million iterations. The sample frequency of the MCMC chain was set as 100 iterations. We set the first 50 million iterations as the burn-in period. The model was identified as convergent when the posterior distribution was approximately normal and the trance of harmonic mean log-likelihoods remained stable across runs. We also plotted the transition rates across the MCMC chain in Tracer^136^ and verified that the effective sample size for the parameters of interest was above 200 (i.e., ESS > 200)^26^. We repeated the run of the MCMC chain ten times and ranked models that were visited by the MCMC chain according to their posterior probability for each run. Ten independent reversible-jump analyses were conducted to generate the highest posterior support RJ-MCMC models, which accounted for 31.79%, 34.74%, 32.01%, 32.96%, 35.54%, 33.54%, 33.36%, 32.99%, 32.28%, and 33.61% of 500,000 visits, respectively; these analyses consistently showed that transitions occurred between any two states of social organization, except from solitary to pair-living. Moreover, group-living was an intermediate step toward pair-living from solitary living.

We initially generated the RJ-MCMC model with the highest posterior support as described above. We subsequently compared four alternative evolutionary models of social organization: ER model, IC model, ARD model, and RJ-MCMC model. Each model was run using the hyper-prior approach (which seeds the mean of the exponential prior from a uniform distribution of 0-2) for 100 million iterations. Every 100 iterations were sampled and the first 50 million iterations were used as the burn-in. We set the stepping-stone sampler to 1000 stones and 10,000 iterations per stone to estimate the marginal likelihood. We conducted ten runs independently for each model. We calculated the variance of the log marginal likelihood values over the ten iterations to represent the stability of the models. The variances of all models were less than 0.03. We compared the four models using Log Bayes Factors (Log BF), calculated using the following equation: Log BF = 2 × (log marginal likelihood complex model − log marginal likelihood simple model). The simple model is favored when Log BF is less than 2, while there is positive evidence to support the complex model when Log BF is greater than 2 (strong evidence, 5–10; very strong evidence, >10)^32^.

#### Evolutionary model of longevity

To determine the evolutionary direction between long-lived and short-lived species, longevity was treated as a categorical variable. Both absolute longevity (i.e., absolute short/long-lived species) and relative longevity (i.e., relative short/long-lived species) were used in the following analyses. Three alternative models were constructed to estimate the evolutionary pathway of longevity: (a) ER model, in which the transition rate from a long-lived to a short-lived state and back is equal; (b) ARD model, in which the transition rate from a long-lived to a short-lived state and back is unequal; (c) RJ-MCMC model (as described above), which is derived from the data by the reversible-jump procedure. The priors, total iterations, burn-in periods, and sampled generations were the same as those used in the social organization models above (hyper-prior approach: the mean of the exponential prior from a uniform distribution of 0-2; iterations: 100 million; sample: 100 iterations; burn-in: the first 50 million iterations; stepping-stone sampler: 1000 stones and 10,000 iterations per stone). Similarly, the same model selection procedure as described for social organization was used to compare the three alternative evolutionary models for longevity. In addition, we repeated ten independent runs for absolute and relative longevity. All models were stable (variances <0.01).

#### Correlated evolution of social organization and longevity

To test whether social organization influences absolute longevity or relative longevity, we first used phylogenetic ANOVA (*phyloAVOVA* function) in the R package phytools^137^ for 974 mammalian species. P-values were adjusted using the Hommel method^138^, and two-sided *p*-values were reported. To control for factors related to external mortality, we also constructed a regression of the Markov chain Monte Carlo sampler for multivariate generalized linear mixed models (MCMCglmm), incorporating the phylogenetic relationship as the covariance structure^139^. In the MCMCglmm model, longevity was fitted as a Gaussian response variable and social organization, adult body mass, activity, lifestyle, and fossoriality as predictor variables. We then tested whether social organization is associated with longevity by analyzing the correlated evolution between the two characteristics using the Discrete package in BayesTraits^140^. Discrete package tests if two binary traits are correlated over a phylogeny by comparing the likelihoods of an independent and dependent model. All possible jumps between the states of each trait are allowed, but in an independent model, the two traits are assumed to evolve independently by placing some restrictions on the transition rate parameters; in a dependent model, which assumes the transitions in one trait depend on the state of the other, the rate parameters are not restricted^140^. To fit the “Discrete” test requirements, we divided social organization into three categories: non-solitary (0) and solitary (1); non-pair-living (0) and pair-living (1); non-group-living (0) and group-living (1); we also divided lifespan into two categories: absolute short-lived (0) and absolute long-lived (1); relative short-lived (0) and long-lived (1). We ran the “Discrete” analysis with a pairwise combination of the three categories of social organization and two categories of lifespan. That is, six “Discrete” analyses: non-solitary/solitary and absolute short-lived/long-lived; non-pair-living/pair-living and absolute short-lived/long-lived; non-group-living/group-living and absolute short-lived/long-lived; non-solitary/solitary and relative short-lived/long-lived; non-pair-living/pair-living and relative short-lived/long-lived; non-group-living/group-living and relative short-lived/long-lived). For each “Discrete” test, RJ-MCMC chains with an independent and dependent model were tested. We used the same procedure as described above to set the model parameters (hyper-prior approach: the mean of the exponential prior from a uniform distribution of 0-2; iterations: 100 million; sample: 100 iterations; burn-in: the first 50 million iterations; stepping-stone sampler: 1000 stones and 10,000 iterations per stone), check model convergence, and compare model performance. RJ-MCMC chains with an independent and dependent model were compared using Log BF. Ten independent runs indicated that all models were stable (variance <0.5).

#### Taxonomic sampling

To evaluate the impact of sample size on the phylogenetic analyses, we followed the taxonomic sampling methods of Kappeler and Pozzi^26^ to subsample the original dataset. We randomly selected species from the original dataset and generated subsets including 95%, 90%, 85%, 80%, 75%, 70%, 65%, 60%, 55%, and 50% of the 974 species; we repeated this step 10 times. A total of 100 subsets were created. Each subset was used to run an RJ-MCMC independent and dependent model of six combinations: non-solitary/solitary and absolute short-lived/long-lived, non-pair-living/pair-living and absolute short-lived/long-lived, non-group-living/group-living and absolute short-lived/long-lived, non-solitary/solitary and relative short-lived/long-lived, non-pair-living/pair-living and relative short-lived/long-lived, non-group-living/group-living and relative short-lived/long-lived.

In addition, to explore the influence of the classification of long-lived species on the phylogenetic analyses, we did not only use 26 years (absolute longevity) and 1.38 (relative longevity) as cut-off values, but also two different cut-off values for absolute longevity (17 years and 35 years) and for relative longevity (0.93 and 1.83). If the longevity of a species was higher than the cut-off value, it was considered to be a long-lived species. For each classification, we randomly sampled the taxa similar to the procedure described above and generated 100 subsets, including 95%, 90%, 85%, 80%, 75%, 70%, 65%, 60%, 55%, and 50% of the original species. Then, we used each subset and compared the RJ-MCMC independent model and the RJ-MCMC dependent model for six combinations: solitary (0/1) and absolute longevity (0/1), pair-living (0/1) and absolute longevity (0/1), and group-living (0/1) and absolute longevity (0/1); solitary (0/1) and relative longevity (0/1), pair-living (0/1) and relative longevity (0/1), and group-living (0/1) and relative longevity (0/1).

### Sample collection and RNA extraction

Comparative transcriptomics is a powerful approach to infer molecular changes underlying complex traits^141–143^. The brain is a central organ that mediates social behavior and social systems, including partner preference^144^, parental care^145^, social hierarchy^146^, eusociality^141,142^, and mating system^28^. Brain transcriptomics has also been used to identify significant genes and pathways that are associated with longevity trait^30,147^. Therefore, we performed comparative transcriptomics of the brain to test whether there are overlapping pathways and genes underlying the correlated evolution between longevity and social organization. We collected 267 fresh brain samples from 94 mammals that encompassed three types of social organization (solitary, pair-living, and group-living) and various longevities (3–122 years). The brain transcriptomes of 101 samples of 42 species were obtained from the published literature^30,148–159^. 57% of species and 62% of samples (166 samples of 54 species) were newly collected in this study. 96% of sampled individuals were adults and 73% of individuals were males (Supplementary Data 2). 71% of 94 species were prepared in more than two biological repeats or technical repeats; the number of repeats for each species are shown in Supplementary Data 2. Since we aimed to characterize the conserved genes and pathways that are related to social organization and longevity among species, the mammal species were chosen based on the availability of brain transcriptome and life-history data, and also the representation of mammal diversity and taxa distribution in the phylogenetic tree. These 94 mammal species are belonging to 14 orders (Artiodactyla, Carnivora, Chiroptera, Cingulata, Didelphimorphia, Diprotodontia, Eulipotyphla, Hyracoidea, Lagomorpha, Monotremata, Perissodactyla, Primates, Rodentia, and Scandentia). We calculated the ratio of species of each order to 974 mammals with longevity available (Ratio_m_), and the ratio of RNA-seq sampled species of each order to 94 species (Ratio_s_). There was no significant difference between Ratio_m_ and Ratio_s_ (Ratio_m_: Median = 2.09%, IQR = 0.38% ~ 7.57%; Ratio_s_: Median = 1.06%, IQR = 1.06% ~ 10.37%; Wilcoxon signed rank test: *n* = 14, *V* = 38, *P* = 0.38), indicating that sampled species in the transcriptomic analyses have a good taxa representation in general. The mean longevity of the 974 mammals and 94 species was 19.55 ± 15.96 and 20.87 ± 16.53, respectively. The medians were 17.15 (IQR = 8.30 ~ 26.18) and 17.40 (IQR = 11.40 ~ 23.55). A summary of the social organization and other life-history traits is shown in Supplementary Table 9.

For the newly collected samples in this study, species identification was performed based on their morphological characteristics and the sequences of the mitochondrial DNA cytochrome oxidase I gene (COI) or cytochrome b gene (cytb). We sampled the forebrain or frontal lobe of the brain of large mammals (e.g., Carnivora) or the entire brain other than the olfactory bulb, cerebellum, pituitary and brain stem for small species (e.g., Chiroptera). We dissected the brains, rapidly froze them in liquid nitrogen, and stored them at −80 °C. Following homogenization of brain tissue, we used the TRIzol protocol to extract total RNA from all samples, according to the user guide (Invitrogen). We assessed the RNA quality and concentration using the Agilent 2100 Bioanalyzer (Lexington, MA, USA) prior to library construction.

### RNA sequencing and gene expression

For each brain RNA sample, a library size of 150 bp was constructed using NEBNext Ultra RNA Library Prep Kit for Illumina (NEB, USA) and paired-end sequencing was performed on the Illumina NovaSeq 6000 platform (Novogene Co. Ltd), generating approximately 2.7 billion reads. We used IllQC_PRLL.pl in the package NGS QC Toolkit to remove low-quality reads of raw data^160^. Draft transcriptomes for 51 without-reference genomes were assembled using Trinity (parameters: k-mer = 25, minimum contig length = 200 bp, paired fragment length = 500 bp, maximum memory = 25 G)^161^. We then ran cd-hit-est (90% similarity) to reduce redundancy in the assembly by retaining the longest transcript in each cluster^162,163^. We applied AUGUSTUS^164^ to predict protein coding genes in non-redundant transcripts using the default parameters, except for –species = human and produced annotation GTF files. Reference genomes for 43 species were publicly available from the NCBI and Ensembl databases.

To generate the species-specific ortholog sets and calculate expression values, we first generated a human reference. We extracted the human CDS sequences using the gffread function of the Cufflinks package^165^. The longest transcript for each gene was extracted after filtering for incomplete and pseudogene transcripts. We used BLAST^166^ to remove highly repetitive or similar genes (cut-offs: e-value <1.0 × 10^−6^ and identity > 90%). We then extracted the longest transcript of each gene for other species and conducted mutual BLAST between each of those species and humans as a reference (cut-offs: e-value <1.0 × 10^−6^ and identity >30%)^167^. Genes with reciprocal best hits were identified as orthologous genes. Given that differences were generated when mapping the transcriptome to whole-genome and de novo genome, we set the CDS of orthologous genes as the reference genome for each species and produced annotation files (GTF format). We used STAR to create an index for each species based on the total sequence size of all orthologous genes and the read length^168^, then aligned the RNA-seq reads to the reference genome using the default parameters in STAR. We counted reads using the featureCounts function of the Subread package^169^ without including multi-mapping reads. Orthologous genes were discarded when the raw counts of genes were low (<10 counts in >3 samples; 2564 genes were removed) or high (read counts >5% of the total counts; 1 gene was removed). Batch effects (Bioproject and sequencing platform) were detected and removed with the comBat_seq function of the R package sva^170^. We kept orthologs that were detected in 70% of the total species (i.e., 66 of 94 species). The number of orthologous genes for different cut-off values was 14,730 (50% of species), 14,152 (60% of species), 13,402 (70% of species), 12,476 (80% of species), 10,138 (90% of species), and 0 (100% of species). 70% was chosen as it was a trade-off between the number of orthologous genes and the number of species that could be used in the sequencing analyses. The gene expression of missing orthologs in a species was treated as missing data. The average number of species used in a model differed for each gene, with a mean of 87.96 ± 6.27 in 13,402 genes. To normalize the raw counts, we scaled the library size (i.e., the number of total reads for each sample) by the TMM (trimmed mean of M-values), and used RPKM (reads per kilobase per million mapped reads) to normalize gene length in the R package edgeR^171^. One was added to the value generated from the RPKM-TMM method before log_2_ transforming to avoid an infinite value. We used these log-transformed gene expressions for all downstream analyses.

### Modeling gene expression and traits

To identify the candidate genes associated with a specific social organization or longevity trait across the 94 species, we conducted MCMCglmm model, incorporating the phylogenetic relationship as the covariance structure in the model^139^. For each of the 13,042 orthologous genes, we constructed four MCMCglmm models; in all four models, the expression value of one gene from the 94 species was fitted as a Gaussian response variable and adult body mass, longevity, activity, diet, lifestyle, and social organization as predictor variables. Since for some genes the species did not include all levels of lifestyle as defined above, we categorized lifestyle into aerial and non-aerial (i.e., terrestrial, arboreal, semi-arboreal, freshwater, marine, and terrestrial-marine) in the gene expression models. The differences between these four models were the different categories of the variable social organization: (a) to identify the solitary-associated genes, all species were classified as solitary and non-solitary in the first MCMCglmm model; (b) to identify the pair-living-associated genes, all species were classified as pair-living and non-pair-living in the second MCMCglmm model; (c) to identify the group-living-associated genes, all species were classified as group-living and non-group-living in the third MCMCglmm model; and (d) all species were classified as solitary, pair-living and group-living in the fourth MCMCglmm model. We used a prior of covariance V of 1.00 and a degree of belief parameter (nu) of 0.002. We ran two MCMC chains for 1 million iterations, with 100,000 burn-in and a thinning of 500 iterations for each mode using the MCMCglmm function in the R package MCMCglmm^172^. Model convergence was declared when Gelman-Rubin’s convergence diagnostic was less than 1.1 using the gelman.diag function in coda package^173^. All effective sample sizes were set at >1000. Genes were considered statistically significant if pMCMC was less than 0.05 and the absolute value of the posterior mean was greater than the cut score. The pMCMC values were used directly because MCMCglmm implements MCMC methods for Bayesian generalized linear mixed models^172,174^. Within a Bayesian framework, in which parameters are estimated based on priors, multiple comparison corrections are not required. Hence, from a Bayesian viewpoint, there is no need to adjust pMCMC^175–179^. The cut score of the posterior mean was calculated using the following steps. Given that the MCMCglmm estimates a value for the posterior mean (similar to the coefficient in linear regression) for each predictor variable in the model, we first plotted a histogram of the posterior mean and fit a high probability distribution (normal or logistic distribution) to the data using the fitdist function in R package fitdistrplus^180^. The possible distributions were compared to obtain the best fit by computing goodness-of-fit statistics using the gofstat function of fitdistrplus. Parameters were estimated when the best fit distribution was chosen. We computed the statistics (e.g., Z-score) of the best fit distribution as the cut score, where each side of the distribution was cut at 0.025 (i.e., a significance level of 0.05 for a two-tailed test). For example, if the data fit a standard normal distribution, the cut score (Z-score) was approximately 1.96. Thus, genes whose pMCMC <0.05 and |posterior mean| > 1.96 were identified as significant genes. A gene with a positive or negative posterior mean had an up- or downregulated expression, respectively. If a significant gene was detected in more than one model, the value of the posterior mean and pMCMC were displayed using the mean and standard deviation (SD) value of these models.

### Tests for relaxed and intensified selection

Multiple sequence alignment of each ortholog gene was performed using the Perl script translatorX.pl^181^, which calls MAFFT for alignment^182^ and GBlock^183^ for filtering unreliable regions (GBlock parameters: b1 = 0.75, b2 = 0.85, b3 = 3, b4 = 5). To test whether genes were under relaxed or intensified selection in solitary, pair-living, group-living, and long-lived species (four hypotheses tests), we ran a likelihood ratio test (LRT) on the 13,402 orthologous genes in RELAX (implemented in HYPHY^65^ using species tree as an input). In tests of the four hypotheses, the 94 species were divided into: (a) solitary, set as test branches, or non-solitary, set as reference branches; (b) pair-living (test) or non-pair-living (reference); (c) group-living (test) or non-group-living (reference); and (d) long-lived (>26 years, test) or short-lived (reference). We compared the model (*K* = 1) and the alternative model allowing *K* to be estimated for each hypothesis test. RELAX estimated three *d*_N_/*d*_S_ rate categories and inferred the selection intensity parameter *K*. *K* < 1 indicates relaxed selection in the test branches, whereas *K* > 1 indicates intensified selection.

### Pathway enrichment analysis

We used the pipeline of the detection of polygenic selection in genesets (polysel), which has the power to detect several genes with small effect mutations that can have a large influence on a biological pathway (see Daub et al., 2013 and 2017 for details)^50^. In addition to the gene sets from Daub et al.^51^, we also generated the gene set of the behavior (GO:0007610) and social behavior (GO:0035176) pathways from GO^184,185^. To detect the pathways associated with social organization or longevity, we used the posterior means (i.e., post mean) of each gene, which were generated from the MCMCglmm models as gene scores. The SUMSTAT score was calculated by summing the gene scores of genes in the set of interesting pathways. In the analysis of upregulated or positive gene sets, the gene score of downregulated or negative genes was set as 0 and vice versa. We used the cor.test function in R to run a correlation between gene score and gene length or species number; if they were significantly correlated (*P* < 0.05), we used RescaleBins to adjust the gene score. Overlapping genes between gene sets were removed in the pruning process. In addition, we used the same procedure of polysel to detect gene sets that were under selection. In pathway enrichment analyses of selection, the gene scores were set using the *K* value of each gene, which was generated from RELAX. The pathway was significant if the *P*-value was less than 0.05 or the absolute of the log_10_ of *P*-value was greater than 1.30.

Two-sided tests were used in all statistical analyses. We used general R packages for plotting, such as ggplot2^186^, dplyr^187^, RColorBrewer^188^, EnvStats^189^, and ggthemes^190^, but also some specific packages. For example, the R packages ggtreeExtra^191^, ggtree^192^, ggstar^193^, tibble^194^, and ggnewscale^195^ were used for the phylogenetic tree plots (Fig. 1a and Fig. 2a). The package vioplot^196^ was used in Fig. 1b and Fig. 1c. Venn diagrams (e.g., Fig. 2b) were plotted with the VennDiagram package^197^. The packages ggpmisc^198^, cowplot^199^, and ggpubr^200^ were used in Fig. 2d and Fig. 2e. The packages pheatmap^201^ and scales^202^ were used to plot the pathway heat maps (e.g., Fig. 2f and Fig. 3e).

### Reporting summary

Further information on research design is available in the Nature Portfolio Reporting Summary linked to this article.

## Supplementary information

Supplementary Information

Description of Additional Supplementary Files

Supplementary Data 1-17

Reporting Summary

## Data Availability

The RNA sequencing data generated in this study have been deposited in the Genome Sequence Archive^203^ in National Genomics Data Center^204^, China National Center for Bioinformation/Beijing Institute of Genomics, Chinese Academy of Sciences under accession code GSA: CRA008468. The species traits data are provided in the Supplementary Data file. Databases used in the data collection of mammalian traits include PanTHERIA [https://esapubs.org/archive/ecol/E090/184/metadata.htm], PHYLACINE [https://zenodo.org/record/1250504#.Y5VZfnZBxnI], Animal Diversity Web [https://animaldiversity.org], GBIF [https://www.gbif.org/], ASM’s Mammal Diversity Database [https://www.mammaldiversity.org/], the Encyclopedia of Life [https://eol.org/docs/what-is-eol], AnAge [https://genomics.senescence.info/species/index.html]. The phylogenetic tree is from TimeTree [https://timetree.org/]. In the comparative transcriptome analyses, we used databases NCBI [https://www.ncbi.nlm.nih.gov/], Ensembl [https://ensemblgenomes.org/], Gene Ontology (GO) [http://geneontology.org/], and Reactome [https://reactome.org/]. Silhouette images of animals used in the figures are from PhyloPic database [http://phylopic.org/]. The SRA accession number and hyperlink of RNA-seq data that were not generated from this study were shown in Supplementary Data 2. Source data are provided with this paper.

## Source data

Source Data

## References

[1] Kappeler PM, van Schaik CP (2002). Evolution of primate social systems. Int. J. Primatol..

[2] Grueter CC (2020). Multilevel organisation of animal sociality. Trends Ecol. Evol..

[3] Nowak MA, Tarnita CE, Wilson EO (2010). The evolution of eusociality. Nature.

[4] Tacutu R (2018). Human ageing genomic resources: new and updated databases. Nucleic Acids Res..

[5] Snyder-Mackler N (2020). Social determinants of health and survival in humans and other animals. Science.

[6] Yang YC (2016). Social relationships and physiological determinants of longevity across the human life span. Proc. Natl Acad. Sci. USA.

[7] Silk JB (2010). Strong and consistent social bonds enhance the longevity of female baboons. Curr. Biol..

[8] Archie EA, Tung J, Clark M, Altmann J, Alberts SC (2014). Social affiliation matters: both same-sex and opposite-sex relationships predict survival in wild female baboons. Proc. R. Soc. B.

[9] Ellis S, Snyder-Mackler N, Ruiz-Lambides A, Platt ML, Brent LJ (2019). Deconstructing sociality: the types of social connections that predict longevity in a group-living primate. Proc. R. Soc. B.

[10] Blumstein DT, Williams DM, Lim AN, Kroeger S, Martin JG (2018). Strong social relationships are associated with decreased longevity in a facultatively social mammal. Proc. R. Soc. B.

[11] Korb J, Heinze J (2021). Ageing and sociality: why, when and how does sociality change ageing patterns?. Philos. Trans. R. Soc. B.

[12] Williams SA, Shattuck MR (2015). Ecology, longevity and naked mole-rats: confounding effects of sociality?. Proc. R. Soc. B.

[13] Healy K (2015). Eusociality but not fossoriality drives longevity in small mammals. Proc. R. Soc. B.

[14] Lukas D, Clutton-Brock T (2012). Life histories and the evolution of cooperative breeding in mammals. Proc. R. Soc. B.

[15] Thorley J (2020). The case for extended lifespan in cooperatively breeding mammals: a re-appraisal. PeerJ.

[16] Lucas ER, Keller L (2020). The co‐evolution of longevity and social life. Funct. Ecol..

[17] Vila J (2021). Social support and longevity: meta-analysis-based evidence and psychobiological mechanisms. Front. Psychol..

[18] Altizer S (2003). Social organization and parasite risk in mammals: integrating theory and empirical studies. Annu. Rev. Ecol. Evol..

[19] Healy K, Ezard TH, Jones OR, Salguero-Gómez R, Buckley YM (2019). Animal life history is shaped by the pace of life and the distribution of age-specific mortality and reproduction. Nat. Ecol. Evol..

[20] Kalbitzer U (2017). Female sociality and sexual conflict shape offspring survival in a Neotropical primate. Proc. Natl Acad. Sci. USA.

[21] Ellis S (2017). Mortality risk and social network position in resident killer whales: sex differences and the importance of resource abundance. Proc. R. Soc. B.

[22] Cameron EZ, Setsaas TH, Linklater WL (2009). Social bonds between unrelated females increase reproductive success in feral horses. Proc. Natl Acad. Sci. USA.

[23] Silk MJ, Hodgson DJ (2021). Differentiated social relationships and the pace-of-life-history. Trends Ecol. Evol..

[24] Shultz S, Opie C, Atkinson QD (2011). Stepwise evolution of stable sociality in primates. Nature.

[25] Lukas D, Clutton-Brock TH (2013). The evolution of social monogamy in mammals. Science.

[26] Kappeler PM, Pozzi L (2019). Evolutionary transitions toward pair living in nonhuman primates as stepping stones toward more complex societies. Sci. Adv..

[27] Clutton-Brock T (2021). Social evolution in mammals. Science.

[28] Young RL (2019). Conserved transcriptomic profiles underpin monogamy across vertebrates. Proc. Natl Acad. Sci. USA.

[29] Ma S (2016). Cell culture-based profiling across mammals reveals DNA repair and metabolism as determinants of species longevity. Elife.

[30] Fushan AA (2015). Gene expression defines natural changes in mammalian lifespan. Aging Cell.

[31] Kappeler PM (2019). A framework for studying social complexity. Behav. Ecol. Sociobiol..

[32] Meade, A. & Pagel, M. BayesTraits V3. http://www.evolution.reading.ac.uk/BayesTraitsV4.0.0/BayesTraitsV4.0.0.html (2019).

[33] Upham NS, Esselstyn JA, Jetz W (2019). Inferring the mammal tree: species-level sets of phylogenies for questions in ecology, evolution, and conservation. PLoS Biol..

[34] Griss J (2020). ReactomeGSA-efficient multi-omics comparative pathway analysis. Mol. Cell. Proteom..

[35] Jassal B (2020). The reactome pathway knowledgebase. Nucleic Acids Res..

[36] Breuer K (2013). InnateDB: systems biology of innate immunity and beyond-recent updates and continuing curation. Nucleic Acids Res..

[37] Bally I (2013). Expression of recombinant human complement C1q allows identification of the C1r/C1s-binding sites. Proc. Natl Acad. Sci. USA.

[38] Sontheimer RD, Racila E, Racila DM (2005). C1q: its functions within the innate and adaptive immune responses and its role in lupus autoimmunity. J. Invest. Dermatol..

[39] Makita S, Takatori H, Nakajima H (2021). Post-transcriptional regulation of immune responses and inflammatory diseases by RNA-Binding ZFP36 family proteins. Front. Immunol..

[40] Moore MJ (2018). ZFP36 RNA-binding proteins restrain T cell activation and anti-viral immunity. Elife.

[41] Petkau G (2022). The timing of differentiation and potency of CD8 effector function is set by RNA binding proteins. Nat. Commun..

[42] Guo H (2022). ZFP36 protects against oxygen-glucose deprivation/reoxygenation-induced mitochondrial fragmentation and neuronal apoptosis through inhibiting NOX4-DRP1 pathway. Brain Res. Bull..

[43] Sjaarda CP (2020). Exome sequencing identifies de novo splicing variant in *XRCC6* in sporadic case of autism. J. Hum. Genet..

[44] Li H, Vogel H, Holcomb VB, Gu Y, Hasty P (2007). Deletion of Ku70, Ku80, or both causes early aging without substantially increased cancer. Cell. Mol. Biol..

[45] Ju YJ (2006). Decreased expression of DNA repair proteins Ku70 and Mre11 is associated with aging and may contribute to the cellular senescence. Exp. Mol. Med..

[46] Parnell E (2021). KALRN: a central regulator of synaptic function and synaptopathies. Gene.

[47] Cahill ME (2009). Kalirin regulates cortical spine morphogenesis and disease-related behavioral phenotypes. Proc. Natl Acad. Sci. USA.

[48] Xie Z (2011). Hippocampal phenotypes in Kalinin-deficient mice. Mol. Cell Neurosci..

[49] Nakajima K (2021). Brain-specific heterozygous loss-of-function of ATP2A2, endoplasmic reticulum Ca^2+^ pump responsible for Darier’s disease, causes behavioral abnormalities and a hyper-dopaminergic state. Hum. Mol. Genet..

[50] Daub JT (2013). Evidence for polygenic adaptation to pathogens in the human genome. Mol. Biol. Evol..

[51] Daub JT, Moretti S, Davydov II, Excoffier L, Robinson-Rechavi M (2017). Detection of pathways affected by positive selection in primate lineages ancestral to humans. Mol. Biol. Evol..

[52] Hollenstein K (2014). Insights into the structure of class B GPCRs. Trends Pharmacol. Sci..

[53] Harmar AJ (2001). Family-B G-protein-coupled receptors. Genome Biol..

[54] Lagunas-Rangel, F. A. G protein-coupled receptors that influence lifespan of human and animal models. *Biogerontology*10.1007/s10522-021-09945-8 (2021). 10.1007/s10522-021-09945-8 PMC8888397 34860303

[55] McDonnell AM, Dang CH (2013). Basic review of the cytochrome p450 system. J. Adv. Pract. Oncol..

[56] Imanikia S, Hylands P, Stürzenbaum SR (2015). The double mutation of cytochrome P450’s and fatty acid desaturases affect lipid regulation and longevity in *C. elegans*. Biochem Biophys. Rep..

[57] Larigot L, Mansuy D, Borowski I, Coumoul X, Dairou J (2022). Cytochromes P450 of *Caenorhabditis elegans*: implication in biological functions and metabolism of xenobiotics. Biomolecules.

[58] Stavropoulou E, Pircalabioru GG, Bezirtzoglou E (2018). The role of cytochromes P450 in infection. Front. Immunol..

[59] Dennis EA, Norris PC (2015). Eicosanoid storm in infection and inflammation. Nat. Rev. Immunol..

[60] Calder PC (2020). Eicosanoids. Essays Biochem..

[61] Evangelista EA, Cho CW, Aliwarga T, Totah RA (2020). Expression and function of eicosanoid-producing cytochrome P450 enzymes in solid tumors. Front. Pharmacol..

[62] Panigrahy D, Kaipainen A, Greene ER, Huang S (2010). Cytochrome P450-derived eicosanoids: the neglected pathway in cancer. Cancer Metastasis Rev..

[63] Omotoso O, Gladyshev VN, Zhou X (2021). Lifespan extension in long-lived vertebrates rooted in ecological adaptation. Front. Cell Dev. Biol..

[64] Robinson GE, Grozinger CM, Whitfield CW (2005). Sociogenomics: social life in molecular terms. Nat. Rev. Genet..

[65] Wertheim JO, Murrell B, Smith MD, Kosakovsky Pond SL, Scheffler K (2015). RELAX: detecting relaxed selection in a phylogenetic framework. Mol. Biol. Evol..

[66] Lawlor MW, Dowling JJ (2021). X-linked myotubular myopathy. Neuromuscul. Disord..

[67] Pierson CR (2012). Modeling the human MTM1 p. R69C mutation in murine Mtm1 results in exon 4 skipping and a less severe myotubular myopathy phenotype. Hum. Mol. Genet..

[68] Widden H, Placzek WJ (2021). The multiple mechanisms of MCL1 in the regulation of cell fate. Commun. Biol..

[69] Robinson EJ (2018). Survival of midbrain dopamine neurons depends on the Bcl2 factor Mcl1. Cell Death Discov..

[70] Arbour N (2008). Mcl-1 is a key regulator of apoptosis during CNS development and after DNA damage. J. Neurosci..

[71] Shigi N (2014). Biosynthesis and functions of sulfur modifications in tRNA. Front. Genet..

[72] Noma A, Sakaguchi Y, Suzuki T (2009). Mechanistic characterization of the sulfur-relay system for eukaryotic 2-thiouridine biogenesis at tRNA wobble positions. Nucleic Acids Res..

[73] Čavužić M, Liu Y (2017). Biosynthesis of sulfur-containing tRNA modifications: a comparison of bacterial, archaeal, and eukaryotic pathways. Biomolecules.

[74] Laxman S (2013). Sulfur amino acids regulate translational capacity and metabolic homeostasis through modulation of tRNA thiolation. Cell.

[75] Yasukawa T (2000). Defect in modification at the anticodon wobble nucleotide of mitochondrial tRNALys with the MERRF encephalomyopathy pathogenic mutation. FEBS Lett..

[76] Rosenbaum DM, Rasmussen SG, Kobilka BK (2009). The structure and function of G-protein-coupled receptors. Nature.

[77] Hu GM, Mai TL, Chen CM (2017). Visualizing the GPCR network: classification and evolution. Sci. Rep..

[78] Heifetz A (2013). Toward an understanding of agonist binding to human Orexin-1 and Orexin-2 receptors with G-protein-coupled receptor modeling and site-directed mutagenesis. Biochemistry.

[79] Krishnan A, Schiöth HB (2015). The role of G protein-coupled receptors in the early evolution of neurotransmission and the nervous system. J. Exp. Biol..

[80] Zhou Q (2019). Common activation mechanism of class A GPCRs. Elife.

[81] Basith S (2018). Exploring G protein-coupled receptors (GPCRs) ligand space via cheminformatics approaches: impact on rational drug design. Front. Pharmacol..

[82] Russell SA, Bashaw GJ (2018). Axon guidance pathways and the control of gene expression. Dev. Dyn..

[83] Bhat AA (2019). Tight junction proteins and signaling pathways in cancer and inflammation: a functional crosstalk. Front. Physiol..

[84] Takano K, Kojima T, Sawada N, Himi T (2014). Role of tight junctions in signal transduction: an update. EXCLI J..

[85] Costea L (2019). The blood-brain barrier and its intercellular junctions in age-related brain disorders. Int. J. Mol. Sci..

[86] Faoro C, Ataide SF (2021). Noncanonical functions and cellular dynamics of the mammalian signal recognition particle components. Front. Mol. Biosci..

[87] Silk JB (2009). The benefits of social capital: close social bonds among female baboons enhance offspring survival. Proc. R. Soc. B.

[88] Nunn CL, Craft ME, Gillespie TR, Schaller M, Kappeler PM (2015). The sociality-health-fitness nexus: synthesis, conclusions and future directions. Philos. Trans. R. Soc. B.

[89] Bourke AF (2007). Kin selection and the evolutionary theory of aging. Annu. Rev. Ecol. Evol. Syst..

[90] Silk JB, Alberts SC, Altmann J (2004). Patterns of coalition formation by adult female baboons in Amboseli, Kenya. Anim. Behav..

[91] Lukas D, Clutton-Brock T (2012). Cooperative breeding and monogamy in mammalian societies. Proc. R. Soc. B.

[92] Schoof VA, Jack KM, Isbell LA (2009). What traits promote male parallel dispersal in primates?. Behaviour.

[93] Sapolsky RM (2005). The influence of social hierarchy on primate health. Science.

[94] Silk JB, Alberts SC, Altmann J (2003). Social bonds of female baboons enhance infant survival. Science.

[95] Ostner J, Schulke O (2018). Linking sociality to fitness in primates: a call for mechanisms. Adv. Study Behav..

[96] Nattrass S (2019). Postreproductive killer whale grandmothers improve the survival of their grandoffspring. Proc. Natl Acad. Sci. USA.

[97] Flurkey K, Papaconstantinou J, Miller RA, Harrison DE (2001). Lifespan extension and delayed immune and collagen aging in mutant mice with defects in growth hormone production. Proc. Natl Acad. Sci. USA.

[98] Junnila RK, List EO, Berryman DE, Murrey JW, Kopchick JJ (2013). The GH/IGF-1 axis in ageing and longevity. Nat. Rev. Endocrinol..

[99] Fontana L, Partridge L, Longo VD (2010). Extending healthy life span-from yeast to humans. Science.

[100] Nunes S, Fite JE, Patera KJ, French JA (2001). Interactions among paternal behavior, steroid hormones, and parental experience in male marmosets (*Callithrix kuhlii*). Horm. Behav..

[101] Remage-Healey L, Maidment NT, Schlinger BA (2008). Forebrain steroid levels fluctuate rapidly during social interactions. Nat. Neurosci..

[102] Ubuka T, Trudeau VL, Parhar I (2020). Steroids and the brain. Front. Endocrinol..

[103] Pérez-Jiménez MM (2021). Steroid hormones sulfatase inactivation extends lifespan and ameliorates age-related diseases. Nat. Commun..

[104] Cremer S, Armitage SA, Schmid-Hempel P (2007). Social immunity. Curr. Biol..

[105] Cohen S, Kaplan JR, Cunnick JE, Manuck SB, Rabin BS (1992). Chronic social stress, affiliation, and cellular immune response in nonhuman primates. Psychol. Sci..

[106] Hermes GL, Rosenthal L, Montag A, McClintock MK (2006). Social isolation and the inflammatory response: sex differences in the enduring effects of a prior stressor. Am. J. Physiol. Regul. Integr. Comp. Physiol..

[107] Yang YC, McClintock MK, Kozloski M, Li T (2013). Social isolation and adult mortality: the role of chronic inflammation and sex differences. J. Health Soc. Behav..

[108] Reed MD (2020). IL-17a promotes sociability in mouse models of neurodevelopmental disorders. Nature.

[109] Lawniczak MK (2007). Mating and immunity in invertebrates. Trends Ecol. Evol..

[110] Kwon JT (2021). An amygdala circuit that suppresses social engagement. Nature.

[111] Singh T, Newman AB (2011). Inflammatory markers in population studies of aging. Ageing Res. Rev..

[112] Nussey DH, Watt K, Pilkington JG, Zamoyska R, McNeilly TN (2012). Age-related variation in immunity in a wild mammal population. Aging Cell.

[113] Kappeler PM, Clutton-Brock T, Shultz S, Lukas D (2019). Social complexity: patterns, processes, and evolution. Behav. Ecol. Sociobiol..

[114] Wilson, D. E. & Mittermeier, R. A. *Handbook of the Mammals of the World. Vol. 2. Hoofed Mammals* (Lynx Ediciones, 2011).

[115] Wilson, D. E. & Mittermeier, R. A. *Handbook of the Mammals of the World. Vol. 1. Carnivores* (Lynx Edictions, 2009).

[116] Wilson, D. E. & Mittermeier, R. A. *Handbook of the Mammals of the World*. *Vol. 4. Sea Mammals* (Lynx Edictions, Barcelona, 2014).

[117] Mittermeier, R. A., Rylands, A. B. & Wilson, D. E. *Handbook of the Mammals of the World. Vol. 3. Primates* (Lynx Edictions, 2013).

[118] Wilson, D. E. & Mittermeier, R. A. *Handbook of the Mammals of the World. Vol. 9. Bats* (Lynx Edicions, 2019).

[119] Jones KE (2009). PanTHERIA: a species-level database of life history, ecology, and geography of extant and recently extinct mammals: Ecological Archives E090‐184. Ecology.

[120] Faurby S (2018). PHYLACINE 1.2: the phylogenetic atlas of mammal macroecology. Ecology.

[121] Penone C (2014). Imputation of missing data in life‐history trait datasets: which approach performs the best?. Methods Ecol. Evol..

[122] Harrison, E., Drake, T. & Ots, R. R package ‘finalfit’: quickly create elegant regression results tables and plots when modelling. https://finalfit.org/index.Html (2021).

[123] Van Buuren S, Groothuis-Oudshoorn K (2011). mice: multivariate imputation by chained equations in R. J. Stat. Softw..

[124] Stekhoven DJ, Bühlmann P (2012). MissForest: non-parametric missing value imputation for mixed-type data. Bioinformatics.

[125] Bruggeman J, Heringa J, Brandt BW (2009). PhyloPars: estimation of missing parameter values using phylogeny. Nucleic Acids Res..

[126] Santos, T. R package ‘PVR’: phylogenetic eigenvectors regression and phylogentic signal-representation curve. https://cran.r-project.org/web/packages/PVR/index.html (2018).

[127] Kissling WD (2014). Establishing macroecological trait datasets: digitalization, extrapolation, and validation of diet preferences in terrestrial mammals worldwide. Ecol. Evol..

[128] Zhou XM (2020). Beaver and naked mole rat genomes reveal common paths to longevity. Cell Rep..

[129] Sen P, Shah PP, Nativio R, Berger SL (2016). Epigenetic mechanisms of longevity and aging. Cell.

[130] Jobson RW, Nabholz B, Galtier N (2010). An evolutionary genome scan for longevity-related natural selection in mammals. Mol. Biol. Evol..

[131] Kumar S, Stecher G, Suleski M, Hedges SB (2017). TimeTree: a resource for timelines, timetrees, and divergence times. Mol. Biol. Evol..

[132] Harmon LJ, Weir JT, Brock CD, Glor RE, Challenger W (2008). GEIGER: investigating evolutionary radiations. Bioinformatics.

[133] Paradis E, Claude J, Strimmer K (2004). APE: analyses of phylogenetics and evolution in R language. Bioinformatics.

[134] Pagel M (1999). Inferring the historical patterns of biological evolution. Nature.

[135] Pagel M, Meade A, Barker D (2004). Bayesian estimation of ancestral character states on phylogenies. Syst. Biol..

[136] Rambaut A, Drummond AJ, Xie D, Baele G, Suchard MA (2018). Posterior summarization in Bayesian phylogenetics using Tracer 1.7. Syst. Biol..

[137] Revell, L. J. R package ‘phytools’: phylogenetic tools for comparative biology (and other things). https://github.com/liamrevell/phytools (2018).

[138] Hommel G (1988). A stagewise rejective multiple test procedure based on a modified Bonferroni test. Biometrika.

[139] Hadfield J, Nakagawa S (2010). General quantitative genetic methods for comparative biology: phylogenies, taxonomies and multi-trait models for continuous and categorical characters. J. Evol. Biol..

[140] Pagel M, Meade A (2006). Bayesian analysis of correlated evolution of discrete characters by reversible-jump Markov chain Monte Carlo. Am. Nat..

[141] Kapheim KM (2016). Genomic sources of phenotypic novelty in the evolution of eusociality in insects. Curr. Opin. Insect Sci..

[142] Chandra V (2018). Social regulation of insulin signaling and the evolution of eusociality in ants. Science.

[143] Huang Z (2019). Longitudinal comparative transcriptomics reveals unique mechanisms underlying extended healthspan in bats. Nat. Ecol. Evol..

[144] Lim MM (2004). Enhanced partner preference in a promiscuous species by manipulating the expression of a single gene. Nature.

[145] Bendesky A (2017). The genetic basis of parental care evolution in monogamous mice. Nature.

[146] Ma M (2020). A novel pathway regulates social hierarchy via lncRNA AtLAS and postsynaptic synapsin IIb. Cell Res..

[147] Lai RW (2019). Multi-level remodeling of transcriptional landscapes in aging and longevity. BMB Rep..

[148] Seim I (2014). The transcriptome of the bowhead whale *Balaena mysticetus* reveals adaptations of the longest-lived mammal. Aging.

[149] Brawand D (2011). The evolution of gene expression levels in mammalian organs. Nature.

[150] Fang X (2014). Adaptations to a subterranean environment and longevity revealed by the analysis of mole rat genomes. Cell Rep..

[151] Martínez-Pacheco M (2020). Expression evolution of ancestral XY gametologs across all major groups of placental mammals. Genome Biol. Evol..

[152] Chen J (2019). A quantitative framework for characterizing the evolutionary history of mammalian gene expression. Genome Res..

[153] Tang QZ (2017). Comparative transcriptomics of 5 high-altitude vertebrates and their low-altitude relatives. Gigascience.

[154] Yan G (2011). Genome sequencing and comparison of two nonhuman primate animal models, the cynomolgus and Chinese rhesus macaques. Nat. Biotechnol..

[155] Carelli FN, Liechti A, Halbert J, Warnefors M, Kaessmann H (2018). Repurposing of promoters and enhancers during mammalian evolution. Nat. Commun..

[156] Fan Y (2013). Genome of the Chinese tree shrew. Nat. Commun..

[157] Qiu Q (2012). The yak genome and adaptation to life at high altitude. Nat. Genet..

[158] Westbury MV, Petersen B, Lorenzen ED (2019). Genomic analyses reveal an absence of contemporary introgressive admixture between fin whales and blue whales, despite known hybrids. PLoS ONE.

[159] Peng X (2015). Tissue-specific transcriptome sequencing analysis expands the non-human primate reference transcriptome resource (NHPRTR). Nucleic Acids Res..

[160] Patel RK, Jain M (2012). NGS QC Toolkit: a toolkit for quality control of next generation sequencing data. PLoS ONE.

[161] Grabherr MG (2011). Full-length transcriptome assembly from RNA-Seq data without a reference genome. Nat. Biotechnol..

[162] Li W, Godzik A (2006). Cd-hit: a fast program for clustering and comparing large sets of protein or nucleotide sequences. Bioinformatics.

[163] Fu L, Niu B, Zhu Z, Wu S, Li W (2012). CD-HIT: accelerated for clustering the next-generation sequencing data. Bioinformatics.

[164] Stanke M (2006). AUGUSTUS: ab initio prediction of alternative transcripts. Nucleic Acids Res..

[165] Trapnell C (2010). Transcript assembly and quantification by RNA-Seq reveals unannotated transcripts and isoform switching during cell differentiation. Nat. Biotechnol..

[166] Boratyn GM (2013). BLAST: a more efficient report with usability improvements. Nucleic Acids Res..

[167] Altschul SF (1997). Gapped BLAST and PSI-BLAST: a new generation of protein database search programs. Nucleic Acids Res..

[168] Dobin A (2013). STAR: ultrafast universal RNA-seq aligner. Bioinformatics.

[169] Liao Y, Smyth GK, Shi W (2014). featureCounts: an efficient general purpose program for assigning sequence reads to genomic features. Bioinformatics.

[170] Leek JT, Johnson WE, Parker HS, Jaffe AE, Storey JD (2012). The sva package for removing batch effects and other unwanted variation in high-throughput experiments. Bioinformatics.

[171] Robinson MD, McCarthy DJ, Smyth GK (2010). edgeR: a Bioconductor package for differential expression analysis of digital gene expression data. Bioinformatics.

[172] Hadfield JD (2010). MCMC methods for multi-response generalized linear mixed models: the MCMCglmm R package. J. Stat. Softw..

[173] Gelman A, Rubin DB (1992). Inference from iterative simulation using multiple sequences. Stat. Sci..

[174] Zhao Y, Staudenmayer J, Coull BA, Wand MP (2006). General design Bayesian generalized linear mixed models. Stat. Sci..

[175] Berry DA, Hochberg Y (1999). Bayesian perspectives on multiple comparisons. J. Stat. Plan. Inference.

[176] Neath AA, Flores JE, Cavanaugh JE (2018). Bayesian multiple comparisons and model selection. Wiley Interdiscip. Rev. Comput. Stat..

[177] Sjölander A, Vansteelandt S (2019). Frequentist versus Bayesian approaches to multiple testing. Eur. J. Epidemiol..

[178] Gelman A, Hill J, Yajima M (2012). Why we (usually) don’t have to worry about multiple comparisons. J. Res. Educ. Eff..

[179] Gelman A, Tuerlinckx F (2000). Type S error rates for classical and Bayesian single and multiple comparison procedures. Comput. Stat..

[180] Delignette-Muller ML, Dutang C (2015). fitdistrplus: an R package for fitting distributions. J. Stat. Softw..

[181] Abascal F, Zardoya R, Telford MJ (2010). TranslatorX: multiple alignment of nucleotide sequences guided by amino acid translations. Nucleic Acids Res..

[182] Katoh K, Misawa K, Kuma KI, Miyata T (2002). MAFFT: a novel method for rapid multiple sequence alignment based on fast Fourier transform. Nucleic Acids Res..

[183] Talavera G, Castresana J (2007). Improvement of phylogenies after removing divergent and ambiguously aligned blocks from protein sequence alignments. Syst. Biol..

[184] Ashburner M (2000). Gene ontology: tool for the unification of biology. Nat. Genet..

[185] The Gene Ontology resource: enriching a GOld mine. *Nucleic Acids Res*. **49**, D325−D334 (2021). 10.1093/nar/gkaa1113 PMC7779012 33290552

[186] Wickham, H. et al. Package ‘ggplot2’: Create elegant data visualisations using the grammar of graphics. R package, version 3.0.0. https://CRAN.R-project.org/package=ggplot2 (2018).

[187] Wickham, H., François, R., Henry, L., Müller, K. & RStudio. Package ‘dplyr’: a grammar of data manipulation. R package, version 1.0.10. https://dplyr.tidyverse.org (2022).

[188] Neuwirth, E. Package ‘RColorBrewer’: ColorBrewer palettes. R package, version 1.1−3. http://colorbrewer2.org (2022).

[189] Millard, S. P. & Kowarik, A. Package ‘EnvStats’: package for environmental statistics, including US EPA guidance. R package, version 2.7.0. https://github.com/alexkowa/EnvStats (2022).

[190] Arnold, J. B. et al. Package ‘ggthemes’: extra themes, scales and geoms for ‘ggplot2’. R package, version 4.2.4. https://github.com/jrnold/ggthemes (2022).

[191] Xu S (2021). ggtreeExtra: compact visualization of richly annotated phylogenetic data. Mol. Biol. Evol..

[192] Yu G, Smith DK, Zhu H, Guan Y, Lam TTY (2017). ggtree: an R package for visualization and annotation of phylogenetic trees with their covariates and other associated data. Methods Ecol. Evol..

[193] Xu, S. Package ‘ggstar’: multiple geometric shape point layer for ‘ggplot2’. R package, version 1.0.3. https://github.com/xiangpin/ggstar/ (2022).

[194] Müller, K., Wickham, H., Francois, R., Bryan, J. & RStudio. Package ‘tibble’: simple data frames. R package, version 3.1.8. *URL* https://tibble.tidyverse.org/ (2022).

[195] Campitelli, E. Package ‘ggnewscale’: multiple fill and colour scales in ‘ggplot2’. R package, version 0.4.8. https://eliocamp.github.io/ggnewscale/ (2022).

[196] Adler, D., Kelly, S. T. & Elliott, T. M. Package ‘vioplot’: violin plot. R package, version 0.3.7. https://github.com/TomKellyGenetics/vioplot (2022).

[197] Chen, H. Package ‘VennDiagram’: generate high-resolution Venn and Euler Plots. R package, version 1.7.3. https://cran.r-project.org/web/packages/VennDiagram/index.html (2022).

[198] Aphalo, P. J., Slowikowski, K. & Mouksassi, S. Package ‘ggpmisc’: miscellaneous extensions to ‘ggplot2’. R package, version 0.5.0. https://github.com/aphalo/ggpmisc (2022).

[199] Wilke, C. O. Package ‘cowplot’: streamlined plot theme and plot annotations for ‘ggplot2’. R package, version 1.1.1. https://wilkelab.org/cowplot/ (2022).

[200] Kassambara, A. Package ‘ggpubr’: ‘ggplot2’ based publication ready plots. R package, version 0.4.0. https://rpkgs.datanovia.com/ggpubr/ (2022).

[201] Kolde, R. Package ‘pheatmap’: pretty heatmaps. R package, version 1.0.12. https://cran.r-project.org/web/packages/pheatmap/index.html (2022).

[202] Wickham, H., Seidel, D. & RStudio. Package ‘scales’: scale functions for visualization. R package, version 1.2.1. https://scales.r-lib.org (2022).

[203] Chen T (2021). The genome sequence archive family: toward explosive data growth and diverse data types. Genom. Proteom. Bioinform..

[204] CNCB-NGDC Members and Partners. (2021). Database resources of the national genomics data center, china national center for bioinformation in 2021. Nucleic Acids Res..

