# Supplementary material for: Correlated evolution of social organization and lifespan in mammals
Source: Nat Commun. 2023 Jan 31;14:372. doi: 10.1038/s41467-023-35869-7 (PMC9889386; doi:10.1038/s41467-023-35869-7)
Supplement: Supplementary file 1 — Supplementary Information [file 41467_2023_35869_MOESM1_ESM.pdf]

Supplementary Information for

Correlated evolution of social organization and lifespan in mammals

Pingfen Zhu[1]†, Weiqiang Liu[1,2]†, Xiaoxiao Zhang[1,2], Meng Li[1], Gaoming Liu[1], Yang Yu[1,3], Zihao Li[1,2], Xuanjing Li[1,2], Juan Du[1,2], Xiao Wang[1], Cyril C. Grueter[4,5,6], Ming Li[1,7]*, Xuming Zhou[1]*

*Corresponding author. Email: lim@ioz.ac.cn (Ming Li); zhouxuming@ioz.ac.cn (X. Zhou). †These authors contributed equally to this work.

Contents

Supplementary Figures 3

Supplementary Figure 1 Alternative evolutionary models of traits. 3
Supplementary Figure 2 The evolutionary pathway of social organization and longevity. 4
Supplementary Figure 3 The effect of taxonomic sampling on the correlated models of social organization and absolute longevity. 5
Supplementary Figure 4 Correlated evolution analysis for social organization and relative longevity. 6
Supplementary Figure 5 The effect of taxonomic sampling on the correlated models of social organization and relative longevity. 7
Supplementary Figure 6 Correlated evolution analyses for social organization and longevity using uni-state sub-datasets.. 8
Supplementary Figure 7 Significant genes whose expression was associated with social organization. 9
Supplementary Figure 8 Significant genes whose expression was associated with longevity. 10
Supplementary Figure 9 Significant pathways of gene expression related to social organization. 11
Supplementary Figure 10 Longevity-related pathways of gene expression were identified in four models. 13
Supplementary Figure 11 Longevity-related pathways of gene expression were detected in each of four models.. 14
Supplementary Figure 12 Genes and pathways under selection in social organization and longevity.. 16

Supplementary Tables 18

Supplementary Table 1 Summary of category traits of 974 mammalian species. 18
Supplementary Table 2 Top ten evolutionary models of social organizations in mammals. 18
Supplementary Table 3 Top five evolutionary models of longevity in mammals. ...20
Supplementary Table 4 Comparative phylogenetic analyses of longevity among different types of social organization.. 22
Supplementary Table 5 Top ten models of correlated evolution of solitary and longevity in mammals. 23
Supplementary Table 6 Top ten models of correlated evolution of group-living and longevity in mammals. 26
Supplementary Table 7 Likelihoods of dependent and independent models estimated for the correlated evolution of social organization and longevity using a different phylogenetic tree. 29
Supplementary Table 8 Likelihoods of dependent and independent models estimated for the correlated evolution of social organization and longevity using uni-state sub-datasets. 30
Supplementary Table 9 Summary of category traits of 94 mammalian species used in the transcriptomic analyses. 31

Supplementary References 32

Supplementary Figures

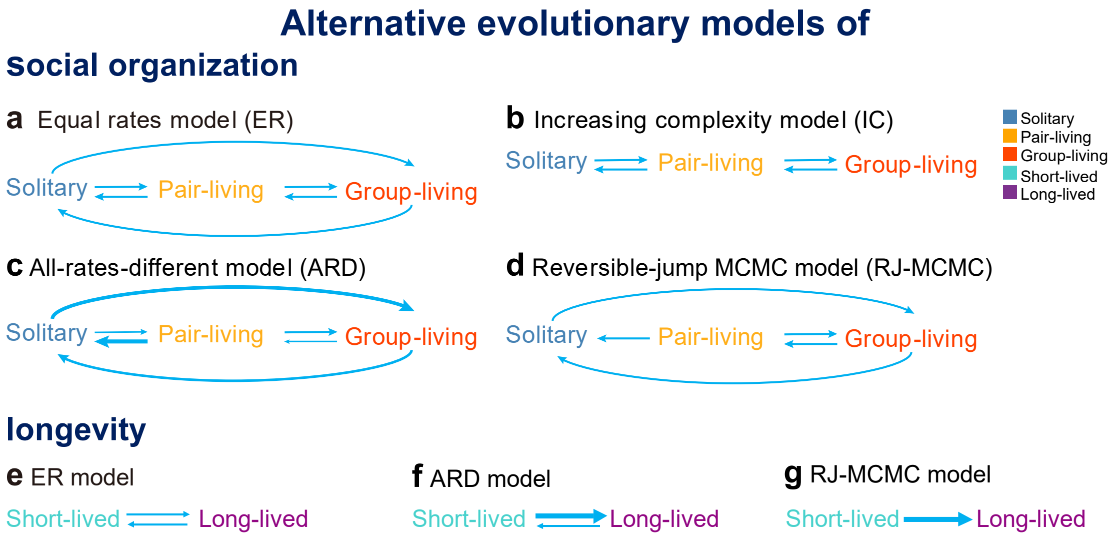

Supplementary Figure 1 Alternative evolutionary models of traits. a-d, social organization and **e-g**, longevity. The ER model (equal rates model): all transition rates are the same. The IC model (increasing complexity model): the model which allows transitions between solitary and pair-living, pair-living and group-living, but not between solitary and group-living. The ARD model (all-rates-different model): all transition rates are different. The RJ-MCMC model (Reversible-jump MCMC model): the model with the highest posterior support that is derived from the data by the reversible-jump procedure in Bayes Traits. Social organization and longevity are colored as follows: solitary = blue; pair-living = orange; group-living = red; short-lived state = cyan; long-lived state = purple. Source data are provided as a Source Data file.

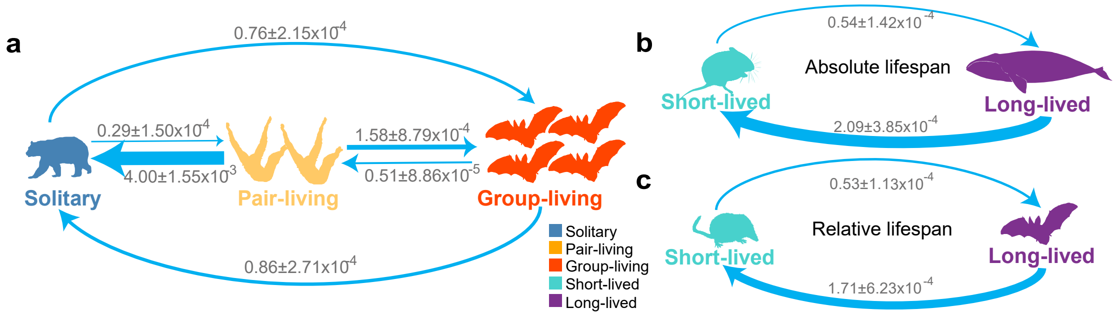

Supplementary Figure 2 The evolutionary pathway of social organization and longevity. **a** The highest posterior support model for the evolution of social organization. Arrows depict the likelihood of a transition between states and their thickness corresponds to the magnitude of the various rates. Numbers indicate the transition rate (mean ± SD) across ten independent runs. **b** Best supported evolutionary model for absolute longevity. Species with a longevity > 26 years were classified as long-lived species. **c** Best supported evolutionary model for relative longevity. Relative long-lived species were species whose residual of longevity was larger than 1.38 (third quartile value). The residual of longevity for each species was calculated using the body mass adjusted residuals with the equation form the AnAge. The number of species used in these analyses was $n = 974$. Social organization and longevity are colored as follows: solitary = blue; pair-living = orange; group-living = red; short-lived state = cyan; long-lived state = purple. Silhouette images of animals are from PhyloPic database (http://phylopic.org/). Source data are provided as a Source Data file.

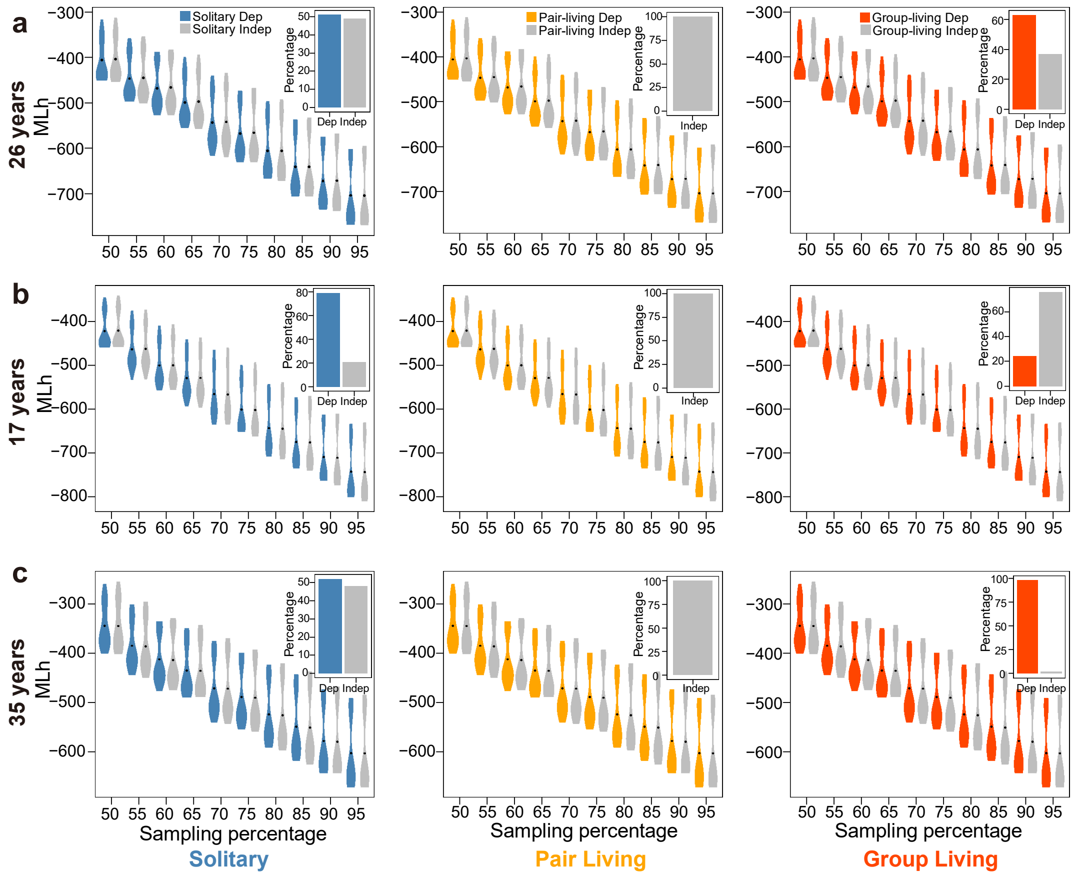

Supplementary Figure 3 The effect of taxonomic sampling on the correlated models of social organization and absolute longevity. Species sampling include 95%, 90%, 85%, 80%, 75%, 70%, 65%, 60%, 55% and 50% of 974 species. A species was classified as long-lived if its maximum lifespan is > 26 years (**a**), > 17 years (**b**) or > 35 years (**c**). In the upper right corner of each plot, bar plots display the supporting proportions of the dependent and independent models. Colors code for social organization: blue = solitary, orange = pair-living and red = group-living. Dep: dependent model; Indep: independent model. Source data are provided as a Source Data file.

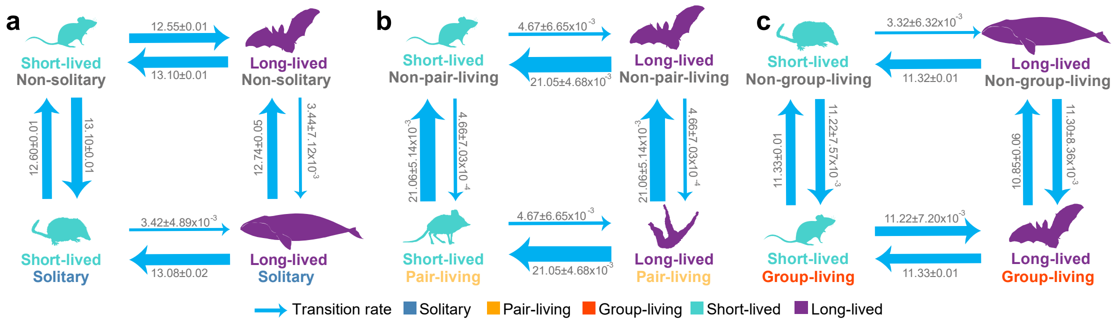

Supplementary Figure 4 Correlated evolution analysis for social organization and relative longevity. Evolutionary models for relative longevity and solitary (**a**); pair-living (**b**); and group-living (**c**). The transition rates from a short-lived to a long-lived state were unequal in **a-c**, showing dependent evolution. Arrows depict the likelihood of transition between states and their thickness corresponds to the magnitude of the various rates. Numbers indicate the transition rate (mean ± SD) across ten independent runs. The number of species used in these analyses was n = 974. Social organization and longevity are colored as follows: solitary: blue; pair-living: orange; group-living: red; short-lived state: cyan; long-lived state: purple. Silhouette images of animals are from PhyloPic database (http://phylopic.org/). Source data are provided as a Source Data file.

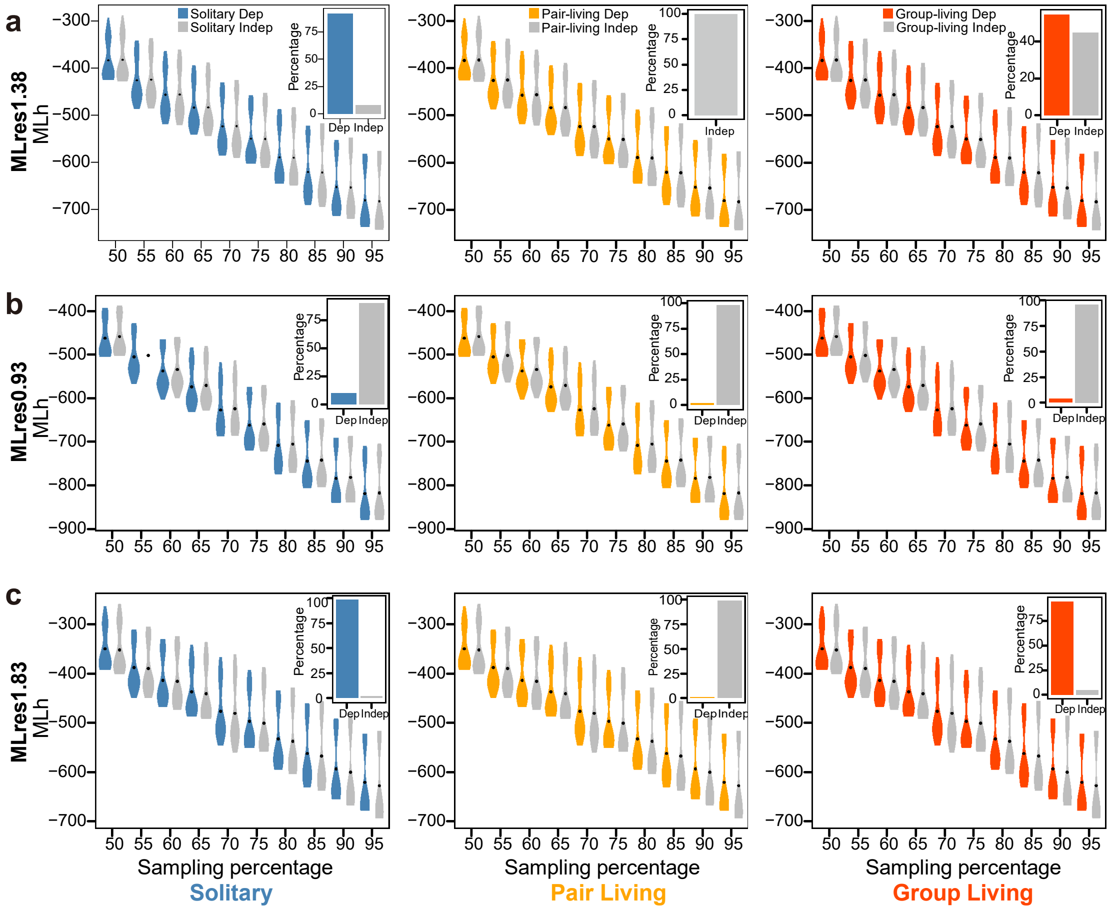

Supplementary Figure 5 The effect of taxonomic sampling on the correlated models of social organization and relative longevity. Species sampling include 95%, 90%, 85%, 80%, 75%, 70%, 65%, 60%, 55% and 50% of 974 species. A species was classified as long-lived if the residual of its maximum lifespan is > 1.38 (**a**), > 0.93 (**b**) or > 1.83 (**c**). In the upper right corner of each plot, bar plots display the supporting proportions of the dependent and independent models. Colors code for social organization and longevity: blue = solitary, orange = pair-living and red = group-living. Dep: dependent model; Indep: independent model. Source data are provided as a Source Data file.

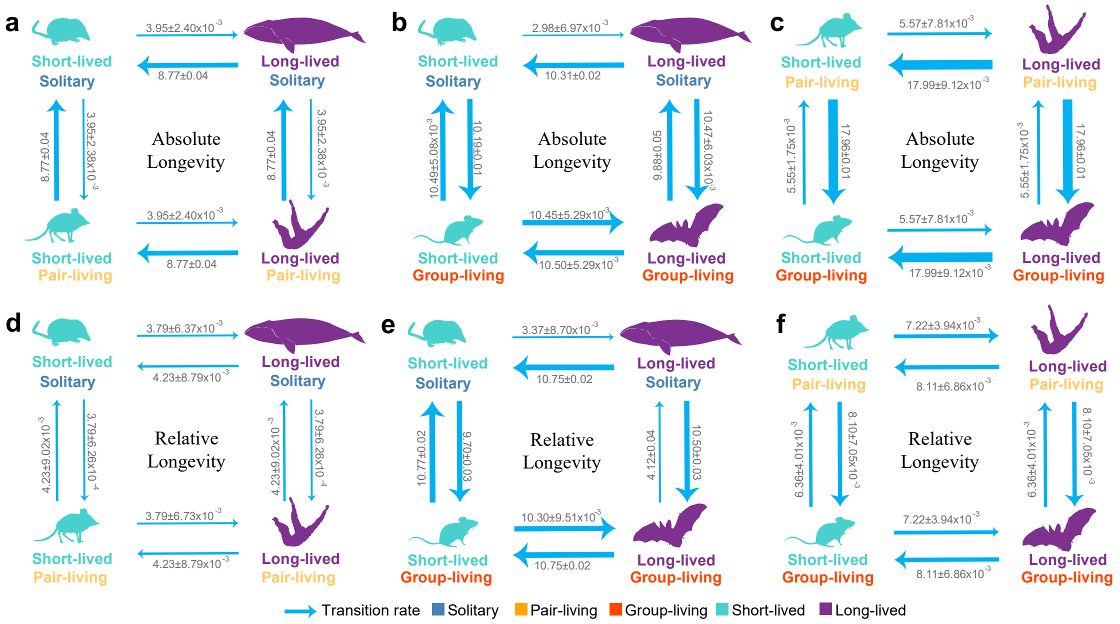

Supplementary Figure 6 Correlated evolution analyses for social organization and longevity using uni-state sub-datasets. Different sub-datasets were used in three different types of models: solitary-pair-living and absolute/relative longevity (species: $n_{\text{uni-state}} = 556$); solitary-group-living and absolute/relative longevity (species: $n_{\text{uni-state}} = 859$); and pair-living-group-living and absolute/relative longevity (species: $n_{\text{uni-state}} = 433$). Evolutionary models for absolute longevity (> 26 years, **a**, **b**, and **c**) and relative longevity (residuals > 1.38, **d**, **e**, and **f**). The transition rates from a short-lived to a long-lived state were equal in **a**, **c**, **d**, **f** showing independent evolution, but were unequal in **b**, **e** showing correlated evolution. Arrows depict the likelihood of transition between states, and their thickness corresponds to the magnitude of the various rates. Numbers indicate the transition rate (mean ± SD) across ten independent runs. Colors code for social organization: blue = solitary, orange = pair-living, red = group-living, cyan = short-lived state, and purple = long-lived state. Silhouette images of animals are from PhyloPic database (http://phylopic.org/). Source data are provided as a Source Data file.

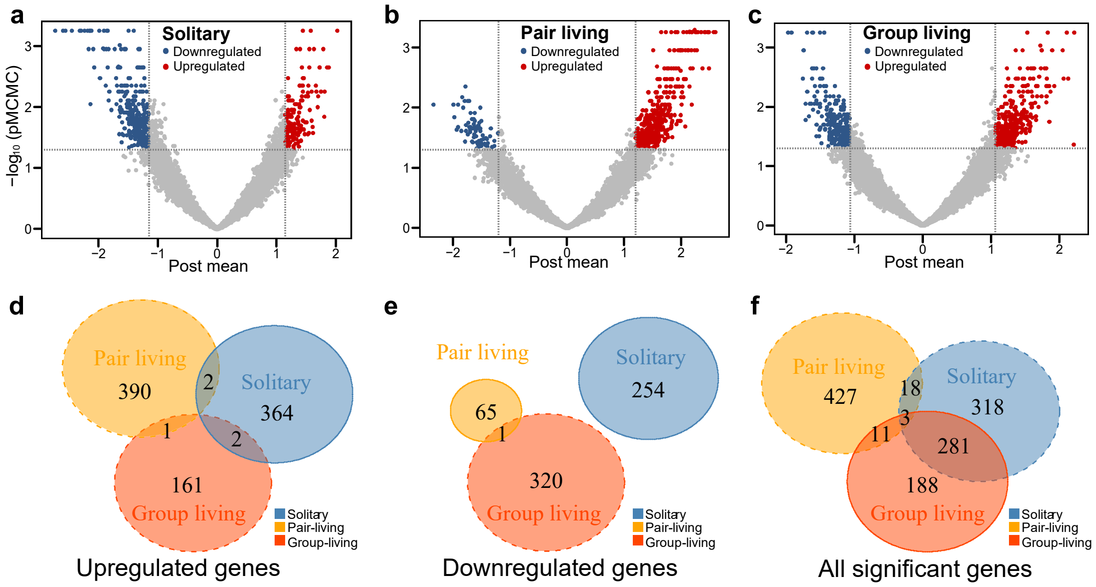

Supplementary Figure 7 Significant genes whose expression was associated with social organization. a Solitary, **b** Pair-living and **c** Group-living. Genes are colored: blue = downregulate genes; red = upregulated genes; gray = non-significant genes. They were generated from MCMCglmm analysis. Genes whose pMCMC < 0.05 and |posterior mean| > cut score (see Methods) were identified as significant genes. A gene with a positive or negative posterior mean had an up- or down-regulated expression, respectively. The number of overlapping genes between solitary, pair-living and group-living are displayed for upregulated genes (**d**) downregulated genes (**e**) and upregulated and downregulated genes (**f**). Blue = solitary, orange = pair-living, and red = group-living. Source data are provided as a Source Data file.

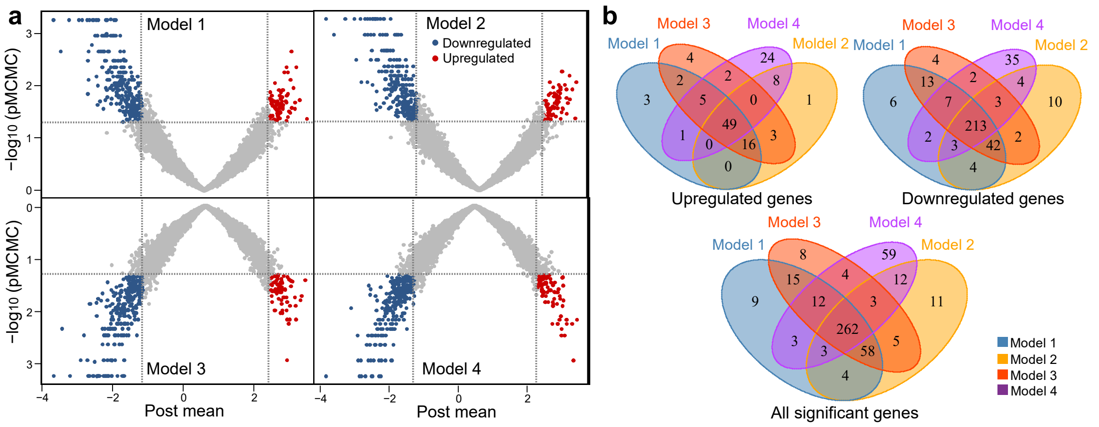

Supplementary Figure 8 Significant genes whose expression was associated with longevity. a Model 1-4. Downregulated genes = blue, upregulated genes = red and non-significant genes = gray. They were generated from MCMCglmm analysis. Genes whose pMCMC < 0.05 and |posterior mean| > cut score (see Methods) were identified as significant genes. A gene with a positive or negative posterior mean had an up- or down-regulated expression, respectively. **b** The number of overlapping upregulated-, downregulated- or all significant- genes in four models. Model 1 = blue, model 2 = yellow, model 3 = red and model 4 = purple. Source data are provided as a Source Data file.

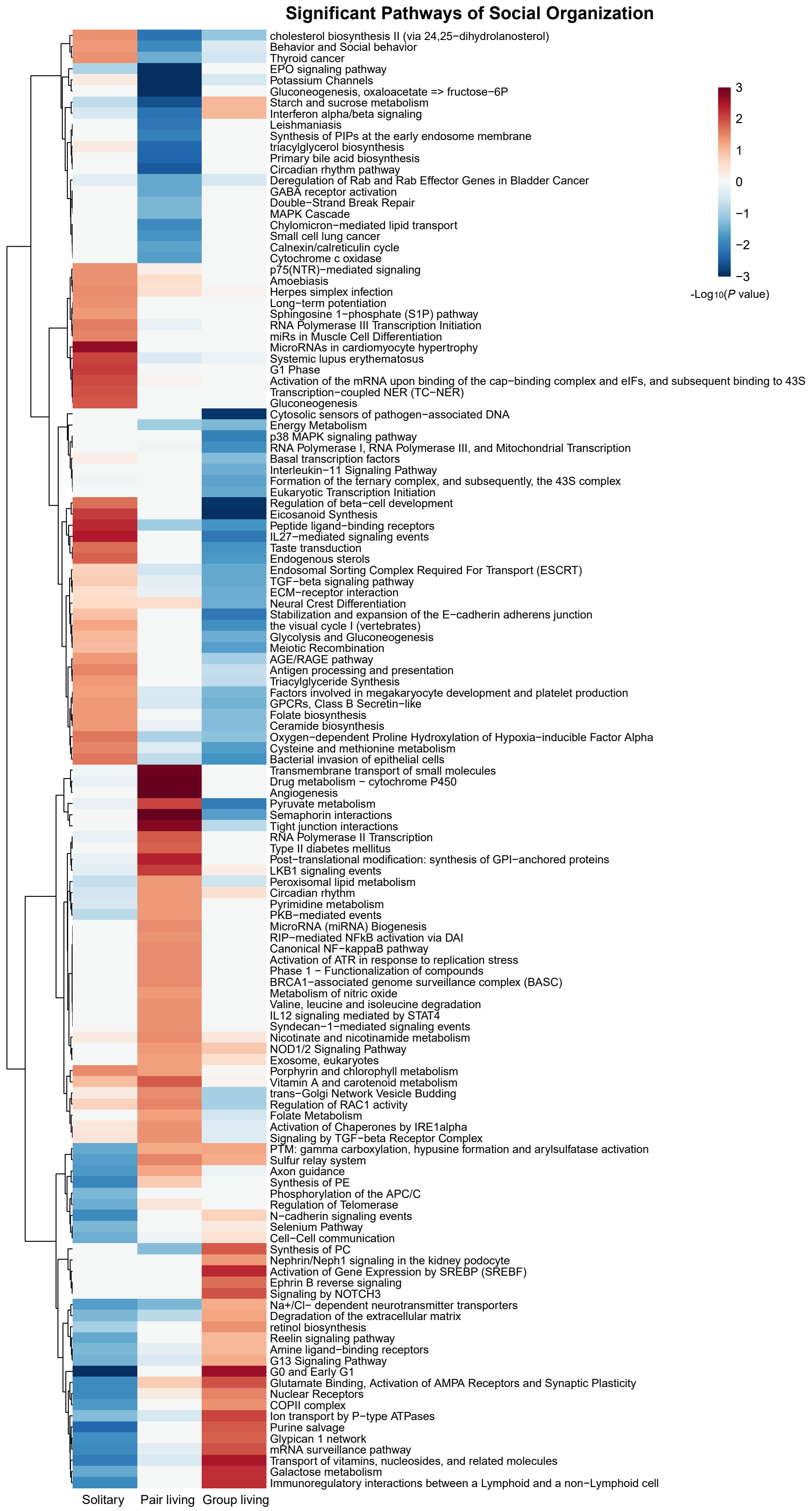

Supplementary Figure 9 Significant pathways of gene expression related to social organization. Polysel method was conducted to detect significant pathways. Post

mean of each of 13,402 orthologous genes from MCMCglmm analysis was used to calculate SUMSTAT score. The pathway is shown if its *P* value is less than 0.05 or the absolute of the log10 of *P* value is greater than 1.30. Source data are provided as a Source Data file.

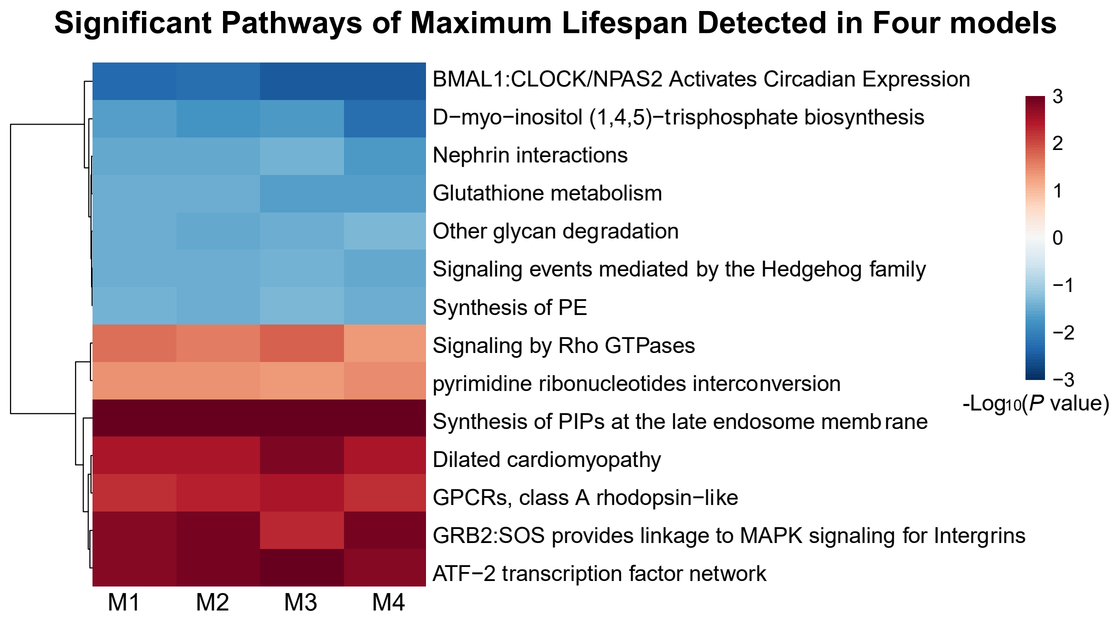

Supplementary Figure 10 Longevity-related pathways of gene expression were identified in four models. M1-M4: model 1 to model 4. Polysel method was conducted to detect significant pathways. Post mean of each of 13,402 orthologous genes from MCMCglmm analysis was used to calculate SUMSTAT score. The pathway is shown if its *P* value is less than 0.05 or the absolute of the log10 of *P* value is greater than 1.30. Source data are provided as a Source Data file.

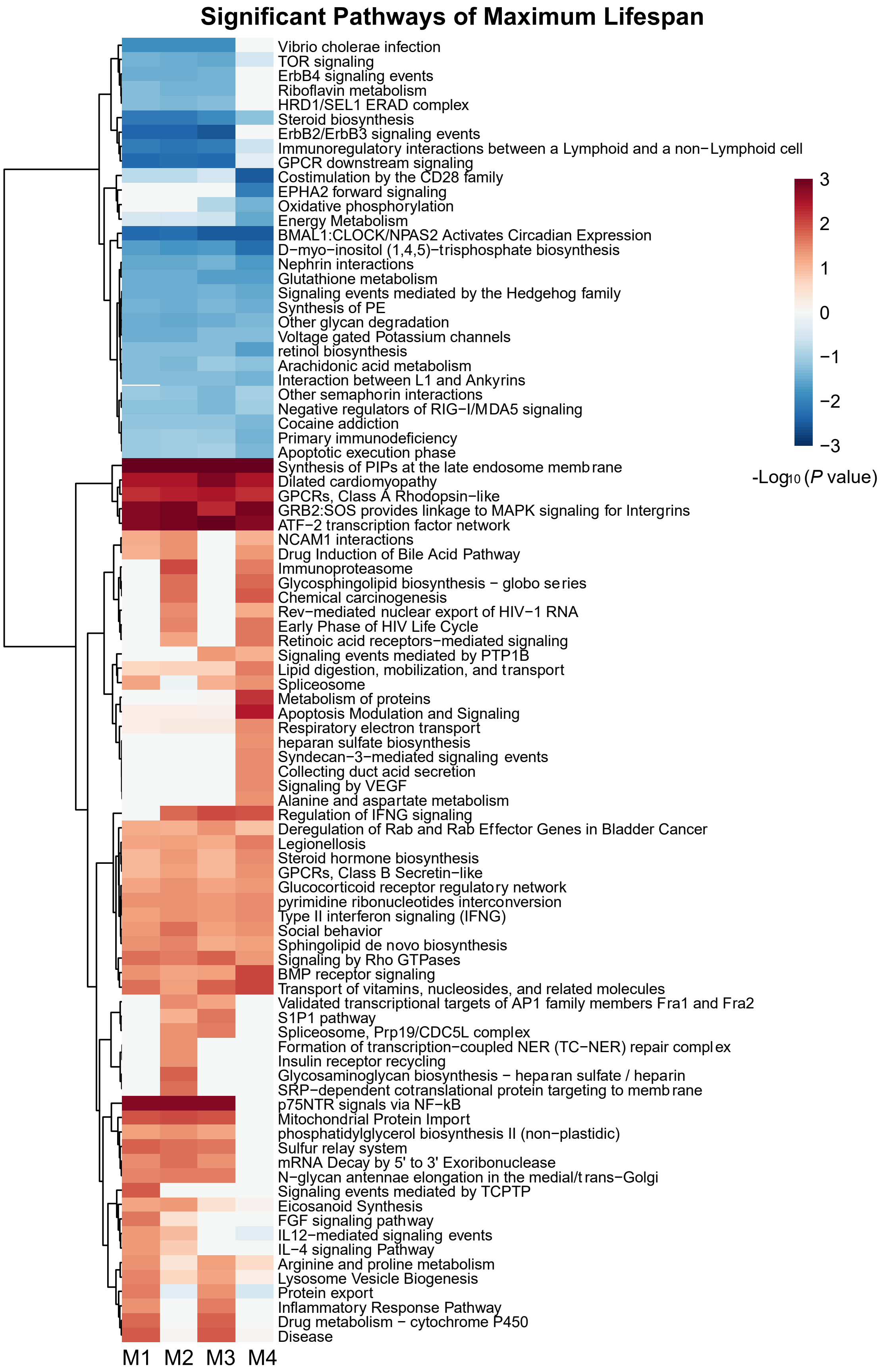

Supplementary Figure 11 Longevity-related pathways of gene expression were detected in each of four models. M1-M4: model 1 to model 4. Polysel method was

conducted to detect significant pathways. Post mean of each of 13,402 orthologous genes from MCMCglmm analysis was used to calculate SUMSTAT score. The pathway is shown if its *P* value is less than 0.05 or the absolute of the log10 of *P* value is greater than 1.30. Source data are provided as a Source Data file.

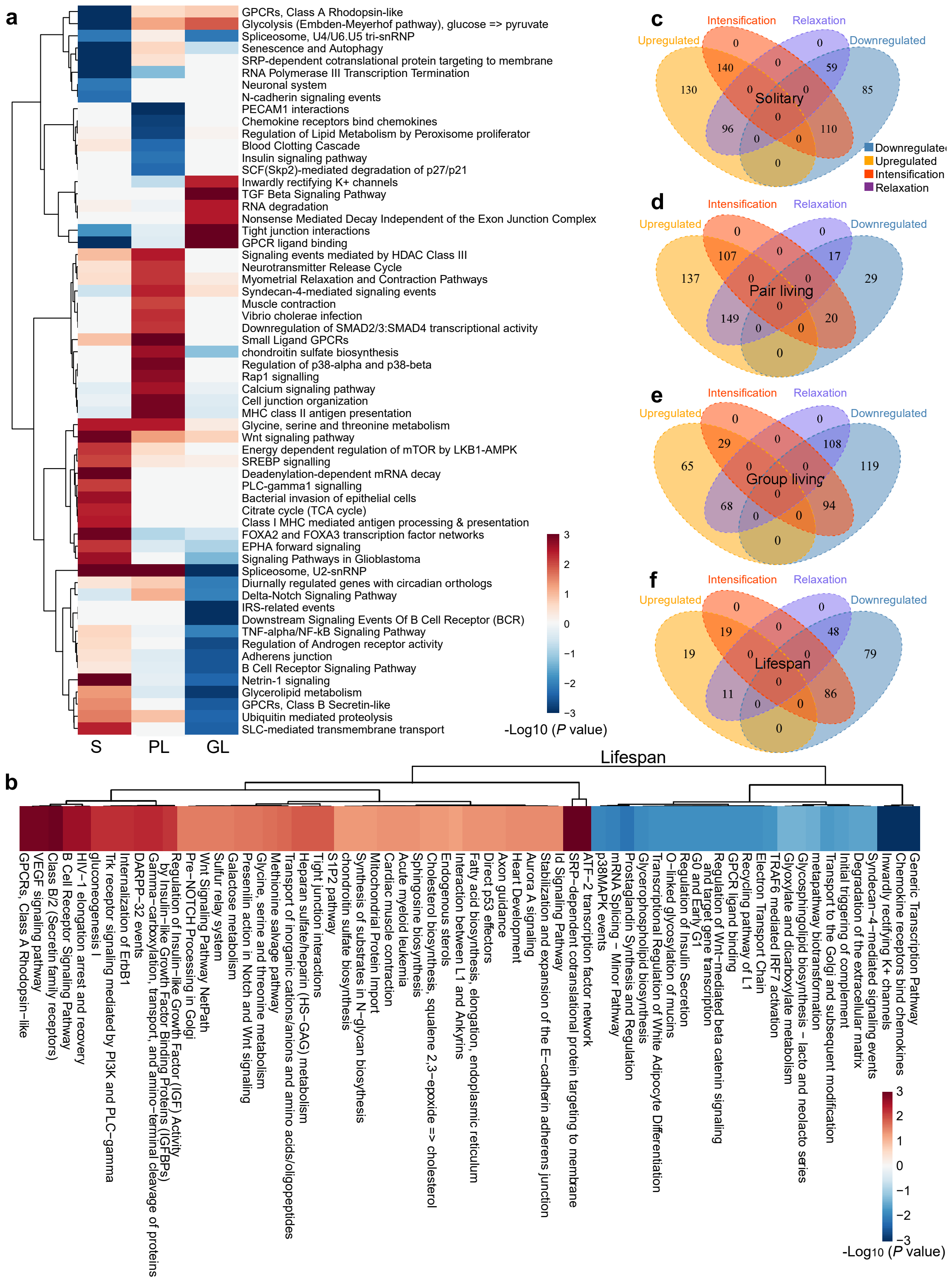

Supplementary Figure 12 Genes and pathways under selection in social organization and longevity. a Pathways under significant intensified or relaxed selection in social organization. S: solitary, PL: pair-living, GL: group-living. Polysel method was conducted to detect significant pathways. The *K* value of each of 13402 orthologous genes from RELAX was used to calculate SUMSTAT score. The pathway is shown if its *P* value is less than 0.01 or the absolute of the log10 of *P* value is greater than two. **b** The pathways under selection in the long-lived state. Polysel with *K* value was used and the pathway is displayed if its *P* value is less than

0.05 or the absolute of the log10 of *P* value is greater than 1.3. The genes who changed expression significantly and also experienced selection in solitary (**c**), pair-living (**d**) and group-living (**e**) and longevity (**f**). Downregulated genes = blue, upregulated genes = yellow, intensification genes = red and relaxation genes = purple. Source data are provided as a Source Data file.

Supplementary Tables

Supplementary Table 1 Summary of category traits of 974 mammalian species.

Trait	Number of species (proportion)			
Social organization	Solitary<br>497 (51.03%)	Pair-living<br>115 (11.81%)	Group-living<br>412 (42.30%)	
Activity	Nocturnal<br>319 (33.69%)	Diurnal<br>320 (33.79%)	Others<br>308 (32.52%)	
Lifestyle	Terrestrial<br>583 (59.85%)	Arboreal and Semiarboreal<br>127 (26.08%)	Aerial<br>63 (6.47%)	Others<br>74 (7.60%)
Fossoriality	Subterranean<br>21 (2.16%)	Nonfossorial<br>953 (97.84%)		

Supplementary Table 2 Top ten evolutionary models of social organizations in mammals. The data of 974 species with social polymorphism was used to conduct ten independent interactions. The top ten models account for 93.31%, 92.92%, 93.28%, 92.87%, 93.67%, 93.22%, 93.28%, 93.25%, 92.51% and 93.50% of the posterior sample for ten runs, respectively. The results of the first three interactions were shown here. Rate coefficient q_{ij} describes the transition rates between state i and j, where the subscripts i and j correspond to two states of three social organizations (0: solitary; 1: pair-living; 2: group-living). Z refers to the rate value of zero while 0s and 1s denote different non-zero transition rates. Frequency = visits to model in posterior sample of 500,000 observations. PDF = probability density; CDF = cumulative density.

Run 1

Model	Rate coefficients						Frequency	PDF	CDF
	q01	q02	q10	q12	q20	q21			
1	Z	1	0	1	1	1	158956	0.32	0.32
2	0	1	1	0	1	0	65003	0.13	0.45
3	1	0	0	1	0	1	49490	0.10	0.55
4	0	1	1	1	1	0	46001	0.09	0.64
5	1	0	0	0	0	1	41375	0.08	0.72
6	Z	0	1	0	0	0	38283	0.08	0.80
7	1	1	0	1	1	1	27705	0.05	0.85
8	0	0	1	1	0	0	17787	0.04	0.89
9	0	1	1	Z	1	0	11703	0.02	0.91
10	1	1	0	0	1	1	10265	0.02	0.93

Run 2

Model	Rate coefficients						Frequency	PDF	CDF
	q01	q02	q10	q12	q20	q21			
1	Z	1	0	1	1	1	173691	0.35	0.35
2	0	1	1	0	1	0	55788	0.11	0.46
3	1	0	0	1	0	1	48895	0.10	0.56
4	0	1	1	1	1	0	38729	0.07	0.63
5	1	0	0	0	0	1	37936	0.08	0.71
6	Z	0	1	0	0	0	37234	0.07	0.78
7	1	1	0	1	1	1	31060	0.06	0.84
8	0	0	1	1	0	0	20857	0.05	0.89
9	1	1	0	0	1	1	10388	0.02	0.91
10	0	1	1	Z	1	0	10015	0.02	0.93

Run 3

Model	Rate coefficients						Frequency	PDF	CDF
	q01	q02	q10	q12	q20	q21			
1	Z	1	0	1	1	1	160048	0.32	0.32
2	0	1	1	0	1	0	57631	0.12	0.44
3	1	0	0	1	0	1	54117	0.10	0.54
4	1	0	0	0	0	1	44305	0.09	0.63
5	0	1	1	1	1	0	41199	0.08	0.71
6	Z	0	1	0	0	0	39550	0.08	0.79
7	1	1	0	1	1	1	27985	0.06	0.85
8	0	0	1	1	0	0	20036	0.04	0.89
9	1	1	0	0	1	1	11183	0.02	0.91
10	0	1	1	Z	1	0	10352	0.02	0.93

Supplementary Table 3 Top five evolutionary models of longevity in mammals. The number of species used in these analyses was *n* = 974. Three of ten independent interactions were shown. Rate coefficient q01 describes the transition rates for the short-lived state to the long-lived state, while q10 represents the transition rates for the long-lived state to the short-lived state (0: short-lived; 1: long-lived). Z refers to the rate value of zero while 0s and 1s denote different non-zero transition rates. Frequency = visits to model in posterior sample of 500,000 observations. PDF = probability density; CDF = cumulative density.

Run 1

Absolute lifespan

Model	Rate coefficients		Frequency	PDF	CDF
	q01	q02			
1	0	1	421873	0.84	0.84
2	1	0	78116	>0.15	>0.99
3	0	0	11	<0.01	1.00

Relative lifespan

Model	Rate coefficients		Frequency	PDF	CDF
	q01	q02			
1	1	0	256682	0.51	0.51
2	0	1	240716	0.48	0.99
3	0	0	2602	0.01	1.00

Run 2

Absolute lifespan

Model	Rate coefficients		Frequency	PDF	CDF
	q01	q02			
1	0	1	371588	0.73176	0.74
2	1	0	128405	>0.25	>0.99
3	0	0	7	<0.01	1.00

Relative lifespan

Model	Rate coefficients		Frequency	PDF	CDF
	q01	q02			
1	1	0	250760	0.50	0.50
2	0	1	246521	0.49	0.99
3	0	0	2719	0.01	1.00

Run 3

Absolute lifespan

Model	Rate coefficients		Frequency	PDF	CDF
	q01	q02			
1	0	1	306174	0.61	0.61
2	1	0	193819	0.38	>0.99
3	0	0	7	<0.01	1.00

Relative lifespan

Model	Rate coefficients		Frequency	PDF	CDF
	q01	q02			
1	1	0	254041	0.51	0.51
2	0	1	243330	0.48	>0.99
3	0	0	2629	<0.01	1.00

Supplementary Table 4 Comparative phylogenetic analyses of longevity among different types of social organization. pMCMC values were calculated using MCMCglmm models. They were fitted with longevity as the response variable and social organization, adult body mass, activity, lifestyle, and fossoriality as predictor variables. Both multi-states (species: *n* = 947) and uni-state (species: *n* = 897) of social organization were considered in the MCMCglmm models. The significant factors are highlighted in bold. The reference categories were solitary for social organization, diurnal for activity, terrestrial for lifestyle, and non-fossorial for fossoriality.

Factors using multi-states	post.mean	95%CI	eff.samp	pMCMC	
(Intercept)	0.64	[0.28, 0.98]	1800	**<0.0006**	***
PairLiving	0.10	[0.05, 0.14]	1800	**0.0011**	**
GroupLiving	0.06	[0.03, 0.10]	1957	**<0.0006**	***
Solitary & PairLiving	0.03	[-0.09, 0.14]	1800	0.6578	
PairLiving & GroupLiving	0.06	[0.01, 0.10]	1800	**0.0322**	*
Activity_Nocturnal	0.04	[-0.01, 0.07]	1800	0.0822	
Activity_Others	0.05	[0.02, 0.08]	1800	**0.0011**	**
Lifestyle_Aerial	0.16	[-0.17, 0.48]	1548	0.3244	
Lifestyle_Arboreal	0.02	[-0.04, 0.08]	1957	0.5133	
Lifestyle_Semiarboreal	0.03	[-0.02, 0.08]	1800	0.2878	
Lifestyle_Freshwater	-0.10	[-0.30, 0.11]	1800	0.3367	
Lifestyle_Marine	-0.06	[-0.22, 0.10]	1551	0.5033	
Lifestyle_Terrestrial & Marine	-0.01	[-0.10, 0.09]	1800	0.8467	
Fossoriality_Subterranean	0.10	[-0.01, 0.21]	1800	0.0667	
$\log_{10}$(AdultBodyMass)	0.15	[0.12, 0.17]	1800	**<0.0006**	***
Species Number	947				
**Factors using uni-state**	**post.mean**	**95%CI**	**eff.samp**	**pMCMC**	
(Intercept)	0.64	[0.29, 1.03]	1663	**0.0011**	**
PairLiving	0.10	[0.06, 0.15]	1979	**<0.0006**	***
GroupLiving	0.06	[0.03, 0.09]	1800	**0.0011**	**
Activity_Nocturnal	0.03	[-0.01, 0.07]	1842	0.1167	
Activity_Others	0.05	[0.02, 0.08]	2357	**0.0056**	**
Lifestyle_Aerial	0.16	[-0.17, 0.47]	1800	0.3267	
Lifestyle_Arboreal	0.02	[-0.04, 0.08]	1800	0.5722	
Lifestyle_Semiarboreal	0.03	[-0.02, 0.08]	1800	0.2467	
Lifestyle_Freshwater	-0.10	[-0.31, 0.11]	1800	0.3511	
Lifestyle_Marine	-0.06	[-0.22, 0.09]	1800	0.4544	
Lifestyle_Terrestrial & Marine	-0.01	[-0.10, 0.10]	1800	0.8511	
Fossoriality_Subterranean	0.09	[-0.04, 0.21]	1800	0.1389	
$\log_{10}$(AdultBodyMass)	0.15	[0.12, 0.17]	1800	**<0.0006**	***
Species Number	897				

Supplementary Table 5 Top ten models of correlated evolution of solitary and longevity in mammals. The data of 974 species with social polymorphism was used to conduct ten independent interactions. The top ten models account for 99.59%, 99.61%, 99.58%, 99.62%, 99.53%, 99.54%, 99.59%, 99.61%, 99.65% and 99.57% of the posterior sample for ten runs, respectively. The results of three interactions were shown here. Rate coefficient q_{ij} describes the transition rates between state: 1 = short-lived/non-solitary; 2 = long-lived/non-solitary, 3 = short-lived/solitary, 4 = long-lived/solitary. That is, for example, q_{12} describes the transition rate from the short-lived state to the long-lived state in non-solitary. Z refers to the rate value of zero while 0s and 1s denote different non-zero transition rates. Frequency = visits to model in posterior sample of 500,000 observations. PDF = probability density; CDF = cumulative density.

Run 1 for absolute longevity (> 26 years)

Model	Rate coefficients								Frequency	PDF	CDF
	q12	q13	q21	q24	q31	q34	q42	q43			
1	1	1	1	1	1	0	1	1	430730	0.86	0.86
2	0	0	0	0	0	1	0	0	39873	0.08	0.94
3	1	1	1	1	1	0	1	0	12875	0.03	0.97
4	1	1	1	0	1	0	1	1	4647	0.01	0.98
5	0	0	0	1	0	1	0	0	4336	0.01	0.99
6	0	0	0	0	0	1	0	1	3487	<0.007	
7	0	0	0	1	0	1	0	1	1089	<0.003	
8	1	1	1	0	1	0	1	0	457	<0.001	
9	2	1	1	2	2	0	1	2	283	<0.001	
10	1	2	2	1	1	0	2	1	282	<0.001	

Run 1 for relative longevity (residuals > 1.38)

Model	Rate coefficients								Frequency	PDF	CDF
	q12	q13	q21	q24	q31	q34	q42	q43			
1	1	1	1	0	1	0	1	1	246299	0.49	0.49
2	0	0	0	1	0	1	0	0	222148	0.45	0.94
3	1	0	0	1	1	1	0	0	5989	0.01	0.95
4	1	1	1	0	1	0	0	1	4149	<0.01	>0.95
5	0	0	0	1	0	1	1	0	3558	<0.01	0.96
6	0	1	1	0	0	0	1	1	3264	<0.01	0.97
7	1	0	0	1	1	1	1	0	1897	<0.01	>0.97
8	1	0	0	1	0	1	0	0	1233	<0.01	<0.98
9	1	1	1	1	1	0	1	1	1160	<0.01	<0.98
10	0	1	1	0	0	0	0	1	1072	<0.01	>0.98

Run 2 for absolute longevity (> 26 years)

Model	Rate coefficients								Frequency	PDF	CDF
	q12	q13	q21	q24	q31	q34	q42	q43			
1	1	1	1	1	1	0	1	1	429406	0.86	0.86
2	0	0	0	0	0	1	0	0	41023	0.08	0.94
3	1	1	1	1	1	0	1	0	16821	0.03	0.97
4	0	0	0	0	0	1	0	1	5979	0.01	0.98
5	0	0	0	1	0	1	0	0	2364	0.01	0.99
6	1	1	1	0	1	0	1	1	1752	<0.004	
7	1	2	2	1	1	0	2	1	268	<0.001	
8	2	1	1	2	2	0	1	2	265	<0.001	
9	1	1	1	1	1	0	1	Z	260	<0.001	
10	2	1	1	2	2	0	1	1	134	<0.001	

Run 2 for relative longevity (residuals > 1.38)

Model	Rate coefficients								Frequency	PDF	CDF
	q12	q13	q21	q24	q31	q34	q42	q43			
1	1	1	1	0	1	0	1	1	235471	0.47	0.47
2	0	0	0	1	0	1	0	0	229460	0.46	0.93
3	1	0	0	1	1	1	0	0	6901	0.01	0.94
4	1	1	1	0	1	0	0	1	4796	0.01	0.95
5	0	0	0	1	0	1	1	0	4268	0.01	0.96
6	0	1	1	0	0	0	1	1	3776	0.01	0.97
7	1	0	0	1	0	1	0	0	1834	<0.01	<0.98
8	1	0	0	1	1	1	1	0	1829	<0.01	<0.98
9	0	1	1	0	0	0	0	1	1668	<0.01	>0.98
10	0	0	0	0	0	1	0	0	1321	<0.01	<0.99

Run 3 for absolute longevity (> 26 years)

Model	Rate coefficients								Frequency	PDF	CDF
	q12	q13	q21	q24	q31	q34	q42	q43			
1	1	1	1	1	1	0	1	1	429152	0.86	0.86
2	0	0	0	0	0	1	0	0	40364	0.08	0.94
3	1	1	1	1	1	0	1	0	15949	0.03	0.97
4	0	0	0	0	0	1	0	1	5551	0.01	0.98
5	1	1	1	0	1	0	1	1	3289	0.01	0.99
6	0	0	0	1	0	1	0	0	2069	<0.005	
7	0	0	0	1	0	1	0	1	439	<0.001	
8	1	1	1	0	1	0	1	0	313	<0.001	
9	1	2	2	1	1	0	2	1	295	<0.001	
10	2	1	1	2	2	0	1	2	258	<0.001	

Run 3 for relative longevity (residuals > 1.38)

Model	Rate coefficients								Frequency	PDF	CDF
	q12	q13	q21	q24	q31	q34	q42	q43			
1	1	1	1	0	1	0	1	1	243882	0.49	0.49
2	0	0	0	1	0	1	0	0	222481	0.44	0.93
3	1	0	0	1	1	1	0	0	5184	0.01	0.94
4	1	1	1	0	1	0	0	1	4949	0.01	0.95
5	0	0	0	1	0	1	1	0	4646	0.01	0.96
6	0	1	1	0	0	0	1	1	3946	0.01	0.97
7	1	0	0	1	1	1	1	0	2533	<0.01	<0.98
8	0	1	1	0	0	0	0	1	1719	<0.01	<0.98
9	0	0	0	0	0	1	0	0	1611	<0.01	>0.98
10	1	0	0	1	0	1	0	0	1423	<0.01	>0.98

Supplementary Table 6 Top ten models of correlated evolution of group-living and longevity in mammals. The data of 974 species with social polymorphism was used to conduct ten independent interactions. The top ten models account for 99.98%, 99.98 %, 99.99%, 99.98%, 99.99%, 99.99%, 99.98%, 99.98%, 99.99% and 99.99% of the posterior sample for ten runs, respectively. The results of the first three interactions were shown here. Rate coefficient q_{ij} describes the transition rates between state: 1 = short-lived/non-group-living; 2 = long-lived/non-group-living, 3 = short-lived/group-living, 4 = long-lived/group-living. That is, for example, q_{12} describes the transition rate from the short-lived state to the long-lived state in non-group-living. Z refers to the rate value of zero while 0s and 1s denote different non-zero transition rates. Frequency = visits to model in posterior sample of 500,000 observations. PDF = probability density; CDF = cumulative density.

Run 1 for absolute longevity (> 26 years)

Model	Rate coefficients								Frequency	PDF	CDF
	q12	q13	q21	q24	q31	q34	q42	q43			
1	0	1	1	1	1	1	1	1	488016	0.97	0.97
2	1	0	0	0	0	0	0	0	11731	0.02	0.99
3	0	1	1	1	1	1	0	1	121	<0.001	
4	0	2	1	1	1	2	2	1	11	<0.001	
5	0	2	2	1	1	2	2	1	9	<0.001	
6	0	1	2	1	2	1	1	2	8	<0.001	
7	0	1	2	2	2	2	2	2	8	<0.001	
8	0	1	2	2	2	1	2	2	7	<0.001	
9	0	2	1	2	1	2	2	1	6	<0.001	
10	0	1	2	2	2	1	1	2	5	<0.001	

Run 1 for relative longevity (residuals > 1.38)

Model	Rate coefficients								Frequency	PDF	CDF
	q12	q13	q21	q24	q31	q34	q42	q43			
1	0	1	1	1	1	1	1	1	406562	0.81	0.81
2	1	0	0	0	0	0	0	0	67726	0.14	0.95
3	1	0	0	0	0	0	1	0	12822	0.02	0.97
4	0	1	1	1	1	1	0	1	7110	0.01	0.98
5	0	0	1	1	1	0	0	1	2665	0.01	0.99
6	1	1	0	0	0	1	1	0	1162	<0.01	>0.99
7	0	0	1	0	1	0	0	1	624	<0.01	
8	1	1	0	1	0	1	1	0	240	<0.001	
9	0	1	1	0	1	1	0	1	152	<0.001	
10	0	0	1	1	1	1	0	1	142	<0.001	

Run 2 for absolute longevity (> 26 years)

Model	Rate coefficients								Frequency	PDF	CDF
	q12	q13	q21	q24	q31	q34	q42	q43			
1	0	1	1	1	1	1	1	1	491265	0.98	0.98
2	1	0	0	0	0	0	0	0	8367	0.01	0.99
3	1	0	0	0	0	0	1	0	146	<0.001	
4	0	1	1	1	1	1	0	1	65	<0.001	
5	1	0	1	0	0	0	0	0	17	<0.001	
6	0	2	1	2	1	2	2	1	14	<0.001	
7	0	1	0	1	1	1	1	1	12	<0.001	
8	0	1	2	1	2	1	1	2	12	<0.001	
9	0	2	1	1	1	2	2	1	8	<0.001	
10	0	1	2	1	2	1	2	2	6	<0.001	

Run 2 for relative longevity (residuals > 1.38)

Model	Rate coefficients								Frequency	PDF	CDF
	q12	q13	q21	q24	q31	q34	q42	q43			
1	0	1	1	1	1	1	1	1	385618	0.77	0.77
2	1	0	0	0	0	0	0	0	86249	0.17	0.94
3	1	0	0	0	0	0	1	0	11689	0.02	0.96
4	0	1	1	1	1	1	0	1	11075	0.02	0.98
5	0	0	1	1	1	0	0	1	2511	<0.01	>0.99
6	1	1	0	0	0	1	1	0	943	<0.01	
7	0	0	1	0	1	0	0	1	550	<0.01	
8	0	1	0	1	1	1	0	1	328	<0.001	
9	1	1	0	1	0	1	1	0	205	<0.001	
10	1	0	0	1	0	0	1	0	94	<0.001	

Run 3 for absolute longevity (> 26 years)

Model	Rate coefficients								Frequency	PDF	CDF
	q12	q13	q21	q24	q31	q34	q42	q43			
1	0	1	1	1	1	1	1	1	499851	0.99	0.99
2	0	1	2	1	2	1	1	2	18	<0.001	
3	0	2	1	2	1	2	2	1	17	<0.001	
4	0	1	2	2	2	1	1	2	8	<0.001	
5	0	2	1	1	1	2	2	1	7	<0.001	
6	0	2	1	1	1	2	1	1	6	<0.001	
7	0	2	1	2	1	1	2	1	6	<0.001	
8	0	1	1	2	2	1	1	2	5	<0.001	
9	0	1	1	2	2	1	2	2	5	<0.001	
10	0	2	1	2	1	2	1	1	5	<0.001	

Run 3 for relative longevity (residuals > 1.38)

Model	Rate coefficients								Frequency	PDF	CDF
	q12	q13	q21	q24	q31	q34	q42	q43			
1	0	1	1	1	1	1	1	1	401162	0.80	0.80
2	1	0	0	0	0	0	0	0	69870	0.14	0.94
3	1	0	0	0	0	0	1	0	12830	0.03	0.97
4	0	1	1	1	1	1	0	1	10595	0.02	0.99
5	0	0	1	1	1	0	0	1	2273	<0.01	>0.99
6	1	1	0	0	0	1	1	0	953	<0.01	
7	0	1	1	0	1	1	0	1	714	<0.01	
8	0	0	1	0	1	0	0	1	521	<0.01	
9	0	1	0	1	1	1	0	1	228	<0.001	
10	1	1	0	1	0	1	1	0	112	<0.001	

Supplementary Table 7 Likelihoods of dependent and independent models estimated for the correlated evolution of social organization and longevity using a different phylogenetic tree. The number of species used in these analyses was n = 974. The phylogenetic tree was from Upham et al., 2019. Absolute long-lived species were categorized using three cut-offs: longevity > 26 years or longevity > 17 years or longevity > 35 years. Relative long-lived species were classified using the body mass adjusted residuals: longevity > 1.38, longevity > 0.93 or longevity > 1.83.

Social states (no/yes)	Longevity states (no/yes)	Mean likelihood of model		Log BF	Correlated evolution
		Dependent	Independent		
Solitary	Absolute long-lived (> 26 years)	-800.83	-801.99	2.32	Yes
Pair living	Absolute long-lived (> 26 years)	-661.14	-654.56	-13.16	No
Group living	Absolute long-lived (> 26 years)	-783.89	-784.15	0.52	No
Solitary	Absolute long-lived (> 17 years)	-822.98	-832.67	19.38	Yes
Pair living	Absolute long-lived (> 17 years)	-697.75	-692.53	-10.44	No
Group living	Absolute long-lived (> 17 years)	-814.53	-816.04	3.02	Yes
Solitary	Absolute long-lived (> 35 years)	-702.69	-702.61	-0.16	No
Pair living	Absolute long-lived (> 35 years)	-540.00	-544.49	8.98	Yes
Group living	Absolute long-lived (> 35 years)	-677.66	-686.79	18.26	Yes
Solitary	Relative long-lived (Residuals > 1.38)	-770.82	-782.61	23.58	Yes
Pair living	Relative long-lived (Residuals > 1.38)	-644.20	-638.00	-12.40	No
Group living	Relative long-lived (Residuals > 1.38)	-759.20	-766.00	13.60	Yes
Solitary	Relative long-lived (Residuals > 0.93)	-924.18	-925.33	2.30	Yes
Pair living	Relative long-lived (Residuals > 0.93)	-798.96	-793.29	-11.34	No
Group living	Relative long-lived (Residuals > 0.93)	-911.33	-913.20	3.74	Yes
Solitary	Relative long-lived (Residuals > 1.83)	-718.98	-735.46	32.96	Yes
Pair living	Relative long-lived (Residuals > 1.83)	-581.79	-575.57	-12.44	No
Group living	Relative long-lived (Residuals > 1.83)	-704.43	-718.62	28.38	Yes

Supplementary Table 8 Likelihoods of dependent and independent models estimated for the correlated evolution of social organization and longevity using uni-state sub-datasets. Different sub-datasets were used in three different types of models: solitary-pair-living models and absolute/relative longevity (species: $n_{\text{uni-state}}$ = 556); solitary-group-living and absolute/relative longevity (species: $n_{\text{uni-state}}$ = 859); and pair-living-group-living and absolute/relative longevity (species: $n_{\text{uni-state}}$ = 433). Absolute long-lived species were categorized using three cut-offs: longevity > 26 years or longevity > 17 years, or longevity > 35 years. Relative long-lived species were classified using the body mass adjusted residuals: longevity > 1.38, longevity > 0.93, or longevity > 1.83.

Social states	Longevity states (no/yes)	Mean likelihood of model Dependent	Independent	Log BF	Correlated evolution
Solitary-Pair living	Absolute long-lived (> 26 years)	-321.69	-319.14	-5.10	No
Solitary-Pair living	Absolute long-lived (> 17 years)	-390.85	-388.36	-4.98	No
Solitary-Pair living	Absolute long-lived (> 35 years)	-257.15	-253.87	-6.56	No
Solitary-Pair living	Relative long-lived (Residuals > 1.38)	-310.37	-307.53	-5.68	No
Solitary-Pair living	Relative long-lived (Residuals > 0.93)	-470.67	-465.67	-10.00	No
Solitary-Pair living	Relative long-lived (Residuals > 1.83)	-247.90	-244.75	-6.30	No
Solitary-Group living	Absolute long-lived (> 26 years)	-659.99	-662.76	5.54	Yes
Solitary-Group living	Absolute long-lived (> 17 years)	-680.46	-684.07	7.22	Yes
Solitary-Group living	Absolute long-lived (> 35 years)	-582.55	-590.02	14.94	Yes
Solitary-Group living	Relative long-lived (Residuals > 1.38)	-630.77	-637.55	13.56	Yes
Solitary-Group living	Relative long-lived (Residuals > 0.93)	-760.14	-758.94	-2.40	No
Solitary-Group living	Relative long-lived (Residuals > 1.83)	-565.70	-577.48	23.56	Yes
Pair living-Group living	Absolute long-lived (> 26 years)	-336.12	-332.15	-7.94	No
Pair living-Group living	Absolute long-lived (> 17 years)	-296.14	-292.67	-6.94	No
Pair living-Group living	Absolute long-lived (> 35 years)	-288.54	-286.74	-3.60	No
Pair living-Group living	Relative long-lived (Residuals > 1.38)	-305.64	-302.86	-5.56	No
Pair living-Group living	Relative long-lived (Residuals > 0.93)	-334.66	-331.36	-6.60	No
Pair living-Group living	Relative long-lived (Residuals > 1.83)	-300.82	-296.67	-8.30	No

Supplementary Table 9 Summary of category traits of 94 mammalian species used in the transcriptomic analyses.

Trait	Number of species (proportion)		
Social organization	Solitary	Pair-living	Group-living
	26 (27.66%)	11 (11.70%)	65 (69.15%)
Activity	Nocturnal	Diurnal	Others
	54 (57.45%)	21 (22.34%)	19 (20.21%)
Diet	Carnivore	Herbivore	Omnivore
	50 (53.19%)	23 (24.47%)	21 (22.34%)
Lifestyle	Non-aerial	Aerial	
	59 (62.77%)	35 (37.23%)	

Supplementary References

References of **Supplementary Data 1** and **Supplementary Data 3 (separate files)**

1. Lukas, D. & Clutton-Brock, T. H. The evolution of social monogamy in mammals. *Science* **341**, 526−530 (2013).
2. Faurby, S. et al. PHYLACINE 1.2: the phylogenetic atlas of mammal macroecology. *Ecology* **99**, 2626 (2018).
3. Tacutu, R. et al. Human ageing genomic resources: new and updated databases. *Nucleic Acids Res.* **46**, D1083−D1090 (2018).
4. Jones, K. E. et al. PanTHERIA: a species-level database of life history, ecology, and geography of extant and recently extinct mammals: ecological Archives E090-184. *Ecology* **90**, 2648−2648 (2009).
5. Thorley, J. The case for extended lifespan in cooperatively breeding mammals: a re-appraisal. *PeerJ* **8**, e9214 (2020).
6. Wilson, D. E. & Mittermeier, R. A. *Handbook of the Mammals of the World. Vol. 2. Hoofed Mammals*. (Lynx Ediciones, Barcelona, 2011).
7. Wilson, D. E. & Mittermeier, R. A. *Handbook of the Mammals of the World. Vol. 4. Sea Mammals.* (Lynx Edictions, Barcelona, 2014).
8. Ben, M. J. & Nouira, S. Social organization of Barbary sheep (*Ammotragus lervia*) population in the Chambi National Park, Tunisia. *Int. J. Biodivers. Conserv.* **5**, 15−19 (2012).
9. Isler, K. & van Schaik, C. P. Allomaternal care, life history and brain size evolution in mammals. *J. Hum. Evol.* **63**, 52−63 (2012).
10. Myers, P. et al. The Animal Diversity Web (online). Accessed at https://animaldiversity.org. (2022).
11. Pérez-Barbería, F. J., Shultz, S. & Dunbar, R. I. Evidence for coevolution of sociality and relative brain size in three orders of mammals. *Evolution* **61**, 2811−2821 (2007).
12. Mittermeier, R. A., Rylands, A. B. & Wilson, D. E. *Handbook of the Mammals of the World. Vol. 3. Primates*. (Lynx Edictions, Barcelona, 2013).
13. West, H. E. & Capellini, I. Male care and life history traits in mammals. *Nat. Commun.* **7**, 11854 (2016).
14. Kamilar, J. M., Bribiescas, R. G. & Bradley, B. J. Is group size related to longevity in mammals? *Biol. Lett.* **6**, 736−739 (2010).
15. Burton, J. A., Hedges, S. & Mustari, A. H. The taxonomic status, distribution and conservation of the lowland anoa *Bubalus depressicornis* and mountain anoa *Bubalus quarlesi*. *Mamm. Rev.* **35**, 25−50 (2005).
16. Arcese, P., Jongejan, G. & Sinclair, A. R. E. Behavioural flexibility in a small African antelope: group size and composition in the oribi (*Ourebia ourebi*, Bovidae). *Ethology* **99**, 1−23 (1995).
17. Weckerly, F. W. Sexual-size dimorphism: influence of mass and mating systems in the most dimorphic mammals. *J. Mammal.* **79**, 33−52 (1998).
18. DeCasien, A. R., Thompson, N. A., Williams, S. A. & Shattuck, M. R. Encephalization and longevity evolved in a correlated fashion in Euarchontoglires but not in other mammals. *Evolution* **72**, 2617−2631 (2018).
19. du Toit, J. T. The feeding ecology of a very small ruminant, the steenbok (*Raphicerus campestris*). *Afr. J. Ecol.* **31**, 35−48 (1993).

20. Stähli, A. *Ungulate Grazers in a Tallgrass Savanna: Implications of Low Resource Quality for Feeding and Mating Strategies*. (University of Zurich, Switzerland, 2012).
21. Rottenberg, H. Coevolution of exceptional longevity, exceptionally high metabolic rates, and mitochondrial DNA-coded proteins in mammals.*Exp. Gerontol.* **42**, 364−373 (2007).
22. Deng, S. et al. Camera trap reveals the co-occurrence patterns of two sympatric muntjac species in southern Anhui Province, China: no spatial segregation. *Ecol. Evol.* **11**, 17801−17809 (2021).
23. Gygax, L. Evolution of group size in the superfamily Delphinoidea (Delphinidae, Phocoenidae and Monodontidae): a quantitative comparative analysis. *Mamm. Rev.* **32**, 295−314 (2002).
24. Nichols, H., Zecherle, L. & Arbuckle, K. Patterns of philopatry and longevity contribute to the evolution of post-reproductive lifespan in mammals. *Biol. Lett.* **12**, 20150992 (2016).
25. Secchi, E. R., Cremer, M. J., Danilewicz, D. & Lailson-Brito, J. A synthesis of the ecology, human-related threats and conservation perspectives for the endangered franciscana dolphin. *Front. Mar. Sci.* **8**, 617956 (2021).
26. Parr, C. S., et al. The encyclopedia of life v2: providing global access to knowledge about life on earth. *Biodivers. Data J.* **29**, e1079 (2014).
27. Lukas, D. & Huchard, E. The evolution of infanticide by males in mammalian societies. *Science* **346**, 841−844 (2014).
28. Wilson, D. E. & Mittermeier, R. A. *Handbook of the Mammals of the World. Vol. 1. Carnivores*. (Lynx Edictions, Barcelona, 2009).
29. Elmhagen, B., Hersteinsson, P., Norén, K., Unnsteinsdottir, E. R. & Angerbjörn, A. From breeding pairs to fox towns: the social organisation of arctic fox populations with stable and fluctuating availability of food. *Polar Biol.* **37**, 111−122 (2014).
30. Schneider, T. C. & Kappeler, P. M. Social systems and life-history characteristics of mongooses. *Biol. Rev.* **89**, 173−198 (2014).
31. Cohas, A. & Allainé, D. Social structure influences extra-pair paternity in socially monogamous mammals. *Biol. Lett.* **5**, 313−316 (2009).
32. Veilleux, C. C. & Kirk, E. C. Visual acuity in mammals: effects of eye size and ecology. *Brain Behav. Evol.* **83**, 43−53 (2014).
33. Le Roux, A. *Communication in the Yellow Mongoose, Cynictis penicillata*. (University of Stellenbosch, Stellenbosch, 2007).
34. Zhou, Y., et al. Hog badger (*Arctonyx collaris*) latrine use in relation to food abundance: evidence of the scarce factor paradox. *Ecosphere* **6**, 1−12 (2015).
35. DeLong, C. M., Wright, C., Fobe, I., Wilcox, K. T. & Morrison, E. North american river otters (*Lontra canadensis*) discriminate between 2D objects varying in shape and color. *Learn. Behav.* **47**, 91−104 (2019).
36. Pontzer, H. & Kamilar, J. M. Great ranging associated with greater reproductive investment in mammals. *Proc. Natl. Acad. Sci. U.S.A.* **106**, 192−196 (2009).
37. Wilson, D. E. & Mittermeier, R. A. *Handbook of the Mammals of the World. Vol. 9. Bats*. (Lynx Edicions, Barcelona, 2019).
38. Wilson, D. E. & Mittermeier, R. A. *Handbook of the Mammals of the World. Vol. 8. Insectivores, Sloths and Colugos*. (Lynx Edicions, Barcelona, 2018).
39. Mittermeier, R. A. & Wilson, D. E. *Handbook of the Mammals of the World. Vol. 5. Monotremes and Marsupials*. (Lynx Edicions, Barcelona, 2015).
40. Tourmente, M., Gomendio, M. & Roldan, E. R. Mass-specific metabolic rate and

sperm competition determine sperm size in marsupial mammals. *PLoS One* **6**, e21244 (2011).
41. Lacher, T. E., Mittermeier, R. A. & Wilson, D. E. *Handbook of the Mammals of the World. Vol. 6. Lagomorphs and Rodents I.* (Lynx Edicions, Barcelona, 2016).
42. Paklina, N. V. & van Orden, C. Numbers, distribution and social structure of Kiang (*Equus kiang* Moorcroft 1841) population in the southwestern part of Tibet, China. *Erforsch. Biol. Ress. Mongolei*, **10**, 213−218 (2007).
43. Kappeler, P. M. & Pozzi, L. Evolutionary transitions toward pair living in nonhuman primates as stepping stones toward more complex societies. *Sci. Adv.* **5**, eaay1276 (2019).
44. Shultz, S., Opie, C. & Atkinson, Q. D. Stepwise evolution of stable sociality in primates. *Nature* **479**, 219−222 (2011).
45. Komers, P. E. & Brotherton, P. N. Female space use is the best predictor of monogamy in mammals. *Proc. Royal Soc. B.* **264**, 1261−1270 (1997).
46. Carbone, C., Cowlishaw, G., Isaac, N. J. & Rowcliffe, J. M. How far do animals go? Determinants of day range in mammals. *Am. Nat.* **165**, 290−297 (2005).
47. Lehmann, J., Korstjens, A. H. & Dunbar, R. I. Group size, grooming and social cohesion in primates. *Anim. Behav.* **74**, 1617−1629 (2007).
48. Eberle, M. & Kappeler, P. M. Family insurance: kin selection and cooperative breeding in a solitary primate (*Microcebus murinus*). *Behav. Ecol. Sociobiol.* **60**, 582−588 (2006).
49. Dröscher, I. & Kappeler, P. M. Defining the low end of primate social complexity: The social organization of the nocturnal white-footed sportive lemur (*Lepilemur leucopus*). *Int. J. Primatol.* **34**, 1225−1243 (2013).
50. Dröscher, I. & Kappeler, P. M. Maintenance of familiarity and social bonding via communal latrine use in a solitary primate (*Lepilemur leucopus*). *Behav. Ecol. Sociobiol.* **68**, 2043−2058 (2014).
51. Elliot, M. & Crespi, B. Placental invasiveness and brain-body allometry in eutherian mammals. *J. Evol. Biol.* **21**, 1763−1778 (2008).
52. Grant, R. A., Delaunay, M. G. & Haidarliu, S. Mystacial whisker layout and musculature in the guinea pig (*Cavia porcellu*s): a social, diurnal mammal. *Anat. Rec.* **300**, 527−536 (2017).
53. Wilson, D. E., Lacher Jr, T. E & Mittermeier, R. A. *Handbook of the Mammals of the World. Vol. 7. Rodents II.* (Lynx Edicions, Barcelona, 2017).
54. Lukas, D. & Huchard, E. The evolution of infanticide by females in mammals. *Philos. Trans. R. Soc. Lond. B Biol. Sci,* **374**, 20180075 (2019).
55. Solomon, N. G., Vandenbergh, J. G. & Sullivan, J., William T. Social influences on intergroup transfer by pine voles (*Microtus pinetorum*). *Can. J. Zool.* **76**, 2131−2136 (1998).
56. Pinheiro, G., Prata, D. F., Araújo, I. M. & Tiscornia, G. The african spiny mouse (*Acomys* spp.) as an emerging model for development and regeneration. *Lab. Anim.* **52**, 565−576 (2018).
57. Viviano, A., Scarfò, M. & Mori, E. Temporal partitioning between forest-dwelling small rodents in a Mediterranean deciduous woodland. *Animals* **12**, 279 (2022).
58. Happold, M. & Happold, D. D. *Mammals of Africa*. (Bloomsbury, London, 2013).
59. Azizi, K., Moemenbellah-Fard, M., Fakoorziba, M. & Fekri, S. *Gerbillus nanus* (Rodentia: Muridae): a new reservoir host of *Leishmania major*. *Ann. Trop. Med. Parasitol.* **105**, 431−437 (2011).

60. Yang, S. W., et al. The effect of grazing on winter survival of midday gerbil (*Meriones meridianus*) of different genders. *Ecol. Evol.* **10**, 12395−12406 (2020).
61. Meeussen, S. E. & Hof, A. R. Predicted future benefits for an endemic rodent in the Irano-Turanian region. *Climate* **9**, 16 (2021).
62. Felt, S. A., Hussein, H. I. & Mohamed Helmy, I. H. Biology, breeding, husbandry and diseases of the captive Egyptian fat-tailed jird (*Pachyuromys duprasi natronensis*). *Lab. Anim.* **37**, 256−261 (2008).
63. Young, L. I., Dickman, C. R., Addison, J. & Pavey, C. R. Spatial ecology and shelter resources of a threatened desert rodent (*Pseudomys australis*) in refuge habitat. *J. Mammal.* **98**, 1604−1614 (2017).
64. Burnett, A. *Behavior and Spatial Ecology of the Harris' Antelope Ground Squirrel (Ammospermophilus harrisii)* (The University of Arizona, Tucson, 2019).
65. Smith, J. R., Van Vuren, D. H., Kelt, D. A. & Johnson, M. L. Spatial organization of northern flying squirrels, *Glaucomys sabrinus*: territoriality in females? *West. N. Am. Nat.* **71**, 44−48 (2011).
66. Radloff, F., et al. A conservation assessment of *Damaliscus pygargus pygargus* in *The Red List of Mammals of South Africa, Swaziland and Lesotho.* (South African National Biodiversity Institute and Endangered Wildlife Trust, South Africa 2016).
67. Tamura, T., Fujise, Y. & Shimazaki, K. Diet of minke whales *Balaenoptera acutorostrata* In: the northwestern part of the North Pacific in summer, 1994 and 1995. *Fish Sci.* **64**, 71−76 (1998).
68. Han, J. Wild yak (*Bos mutus* Przewalski, 1883). In: *Ecology, Evolution and Behaviour of Wild Cattle: Implications for Conservation*, 194−215 (2014).
69. Kissling, W. D. et al. Establishing macroecological trait datasets: digitalization, extrapolation, and validation of diet preferences in terrestrial mammals worldwide. *Ecol. Evol.* **4**, 2913−2930 (2014).
70. Speakman, J. R. Chiropteran nocturnality. In: *Symposia of the zoological society of London*. (The Society, London, 1995).
71. Liu, S. & Wu, Y. *Handbook of the Mammals of China*. (The Straits, Fuzhou, 2019).
72. Douangboubpha, B., et al. A taxonomic review of the *Hipposideros bicolor* species complex and *H. pomona* (Chiroptera: Hipposideridae) in Thailand. *Acta Chiropt.* **12**, 415−438 (2010).
73. Zhang, C., et al. Geographical variation in the echolocation calls of bent-winged bats, *Miniopterus fuliginosus*. *Zoology* **131**, 36−44 (2018).
74. Kimprasit, T., et al. Dispersal history of *Miniopterus fuliginosus* bats and their associated viruses in east Asia. *PLoS One* **16**, e0244006 (2021).
75. Sun, K. P., et al. A new cryptic species of *Rhinolophus macrotis* (Chiroptera: Rhinolophidae) from Jiangxi Province, China. *Acta Chiropt.* **10**, 1−10 (2008).
76. Stankowich, T. & Campbell, L. A. Living in the danger zone: exposure to predators and the evolution of spines and body armor in mammals. *Evolution* **70**, 1501−1511 (2016).
77. Soisook, P., et al. The first record of *Ia io* Thomas, 1902 (Mammalia: Chiroptera: Vespertilionidae) from the Sundaic Subregion, with a description of a new subspecies from peninsular Thailand. *Zootaxa* **4344**, 573−588 (2017).
78. Gao, W., Chen, W. & Fu, B. The fauna and distribution of Chiropteran in Beijing Area. *J. Hebei Norm. Univ., Nat. Sci. Ed.* **16**, 49−52 (1996).
79. Shi, H., Liu, H., Wu, Y. & Liu, Z. Species diversity and threatened status of cave-

dwelling bats in Mianyang, Sichuan. *Sichuan J. Zool.* **25**, 128−131 (2006).
80. Ruedi, M., Csorba, G., Lin, L. K. & Chou, C. H. Molecular phylogeny and morphological revision of Myotis bats (Chiroptera: Vespertilionidae) from Taiwan and adjacent China. *Zootaxa* **3920**, 301−342 (2015).
81. Smith, A. T., et al. *A Guide to the Mammals of China* (Princeton University Press, New Jersey, 2010).
82. Hao, Z. et al. Habitat selection and the response to human disturbances by *Anourosorex squamipes*. *Acta Theriol. Sin.* **37**, 266 (2017).
83. Cowl, V. B. & Shultz, S. Large brains and groups associated with high rates of agonism in primates. *Behav. Ecol.* **28**, 803−810 (2017).
84. Svendsen, G. E. Population parameters and colony composition of beaver (*Castor canadensis*) in southeast Ohio. *Am. Midl. Nat.* **104**, 47−56 (1980).
85. Liu, W., Wang, G., Wang, Y., Zhong, W. & Wan, X. Population ecology of wild mongolian gerbils *Meriones unguiculatus*. *J. Mammal.* **90**, 832−840 (2009).
86. Stockley, P. Female multiple mating behaviour, early reproductive failure and litter size variation in mammals. *Proc. R. Soc. Lond. B Biol. Sci.* **270**, 271−278 (2003).
87. Tamura, N. & Miyashita, K. Diurnal activity of the Formosan squirrel, *Callosciurus erythraeus thaiwanensis*, and its seasonal change with feeding. *J. Mammal. Soc. Jpn.* **10**, 37−40 (1984).
88. Sato, T. & Kawamura, H. Effects of bilateral suprachiasmatic nucleus lesions on the circadian rhythms in a diurnal rodent, the Siberian chipmunk (*Eutamias sibiricus*). *J. Comp. Physiol.* **155**, 745−752 (1984).
