# Supplementary material for: Correlated evolution of social organization and lifespan in mammals
Source: Nat Commun. 2023 Jan 31;14:372. doi: 10.1038/s41467-023-35869-7 (PMC9889386; doi:10.1038/s41467-023-35869-7)
Supplement: Supplementary file 2 — Description of Additional Supplementary Files [file 41467_2023_35869_MOESM2_ESM.pdf]

Legends of Supplementary Data 1-17

(1) Supplementary Data 1: Social organization and longevity of 974 mammalian species. The species name and classification followed the criterion from the Mammal Diversity Database, which has updated the new describing species and taxonomic changes since the 3rd edition of Mammal Species of the World (2005). The data were collected from published literature, reviews, and online databases. References are provided in the Supplementary Information.

(2) Supplementary Data 2: Species and samples used in RNA-seq sequencing and analyses.

(3) Supplementary Data 3: Six traits of 94 mammalian species used in RNA-seq sequencing and analyses. The traits were collected from published literature, reviews, and online databases. References are provided in the Supplementary Information.

(4) Supplementary Data 4: List of genes whose expression was significantly related to social organization. They were generated from MCMCglmm analysis. Genes whose pMCMC < 0.05 and |posterior mean| > cut score (see Methods) were identified as significant genes. A gene with a positive or negative posterior mean had an up- or down-regulated expression, respectively.

(5) Supplementary Data 5: List of genes whose expression was significantly correlated to longevity in four models. They were generated from MCMCglmm analysis. Significant genes were generated from MCMCglmm analysis. Genes whose pMCMC < 0.05 and |posterior mean| > cut score (see Methods) were identified as significant genes. A gene with a positive or negative posterior mean had an up- or down-regulated expression, respectively. If a significant gene was detected in more than one model, the value of the posterior mean and pMCMC were displayed using the mean and standard deviation (SD) value of these models.

(6) Supplementary Data 6: List of genes whose expression was significantly correlated to longevity in each of the four models. They were generated from MCMCglmm analysis. Genes whose pMCMC < 0.05 and |posterior mean| > cut score (see Methods) were identified as significant genes. A gene with a positive or negative posterior mean had an up- or down-regulated expression, respectively.

(7) Supplementary Data 7: List of overlapping genes whose expression was significantly related to both social organization and longevity. Significant genes were generated from MCMCglmm analysis. Genes whose pMCMC < 0.05 and |posterior mean| > cut score (see Methods) were identified as significant genes. A gene with a positive or negative posterior mean had an up- or down-regulated expression, respectively. If a significant gene was detected in more than one model, the value of

the posterior mean and pMCMC were displayed using the mean and standard deviation (SD) value of these models.

(8) Supplementary Data 8: List of pathways with gene expression were significantly related to social organization from the polysel analysis. Polysel method was conducted to detect significant pathways. Post mean of each of 13,402 orthologous genes from MCMCglmm analysis was used to calculate SUMSTAT score. The pathway is included in the list if its P value < 0.05.

(9) Supplementary Data 9: Pattern of the pathways associated with longevity in four models. Polysel method was conducted to detect significant pathways. Post mean of each of 13,402 orthologous genes from MCMCglmm analysis was used to calculate SUMSTAT score. The pathway is significant if its P value < 0.05.

(10) Supplementary Data 10: Pattern of the pathways associated with longevity in each of four models. Polysel method was conducted to detect significant pathways. Post mean of each of 13,402 orthologous genes from MCMCglmm analysis was used to calculate SUMSTAT score. The pathway is significant if its P value < 0.05.

(11) Supplementary Data 11: List of pathways associated with both social organization and longevity in gene expression analysis. Polysel method was conducted to detect significant pathways. Post mean of each of 13,402 orthologous genes from MCMCglmm analysis was used to calculate SUMSTAT score. The pathway is significant if its P value < 0.05.

(12) Supplementary Data 12: List of genes under selection in solitary, pair-living, or group-living groups. They were generated from RELAX analysis. The P value was calculated using the Likelihood-ratio test (LRT). In tests of the three hypotheses, the 94 species were divided into a) solitary, set as test branches, or non-solitary, set as reference branches; b) pair-living (test) or non-pair-living (reference); and c) group-living (test) or non-group-living (reference). The gene whose P < 0.05 is included in the list. K < 1 indicates relaxed selection in the test branches, whereas K > 1 indicates intensified selection.

(13) Supplementary Data 13: List of genes under selection in the branch leading to long-lived mammals. They were generated from RELAX analysis. The P value was calculated using the Likelihood-ratio test (LRT). In the hypothesis test, long-lived species were set as test branches, and short-lived species were set as reference branches. The gene whose P < 0.05 is included in the list. K < 1 indicates relaxed selection in the test branches, whereas K > 1 indicates intensified selection.

(14) Supplementary Data 14: List of pathways under intensified or relaxed selection for social organization. Polysel method was conducted to detect significant

pathways. The *K* value of each of 13402 orthologous genes from RELAX was used to calculate SUMSTAT score. The pathway whose $P < 0.05$ is included in the list.

(15) Supplementary Data 15: List of pathways under selection for longevity. Polysel method was conducted to detect significant pathways. The *K* value of each of 13,402 orthologous genes from RELAX was used to calculate SUMSTAT score. The pathway whose $P < 0.05$ is included in the list.

(16) Supplementary Data 16: List of pathways under selection for both social organization and longevity. Polysel method was conducted to detect significant pathways. The *K* value of each of 13402 orthologous genes from RELAX was used to calculate SUMSTAT score. The pathway whose $P < 0.05$ is included in the list.

(17) Supplementary Data 17: List of genes subjected to significant expression and selection for both social organization and longevity. Significant expression genes were generated from MCMCglmm analysis. Genes whose pMCMC < 0.05 and |posterior mean| > cut score (see Methods) were identified as significant genes. A gene with a positive or negative posterior mean had an up- or down-regulated expression, respectively. Significant selection genes were generated from RELAX analysis. The *P* value was calculated using the Likelihood-ratio test (LRT). In tests of the four hypotheses, the 94 species were divided into: a) solitary, set as test branches, or non-solitary, set as reference branches; b) pair-living (test) or non-pair-living (reference); and c) group-living (test) or non-group-living (reference); and d) long-lived (test) or short-lived (reference). The gene whose $P < 0.05$ is included in the list. $K < 1$ indicates relaxed selection in the test branches, whereas $K > 1$ indicates intensified selection.
