# Supplementary material for: Correlated evolution of social organization and lifespan in mammals
Source: Nat Commun. 2023 Jan 31;14:372. doi: 10.1038/s41467-023-35869-7 (PMC9889386; doi:10.1038/s41467-023-35869-7)
Supplement: Supplementary file 4 — Reporting Summary [file 41467_2023_35869_MOESM4_ESM.pdf]

nature portfolio

Corresponding author(s): Ming Li & Xuming Zhou

Last updated by author(s): Dec 12, 2022

Reporting Summary

Nature Portfolio wishes to improve the reproducibility of the work that we publish. This form provides structure for consistency and transparency in reporting. For further information on Nature Portfolio policies, see our Editorial Policies and the Editorial Policy Checklist.

Statistics

For all statistical analyses, confirm that the following items are present in the figure legend, table legend, main text, or Methods section.

n/a	Confirmed	
☐	☒	The exact sample size (*n*) for each experimental group/condition, given as a discrete number and unit of measurement
☐	☒	A statement on whether measurements were taken from distinct samples or whether the same sample was measured repeatedly
☐	☒	The statistical test(s) used AND whether they are one- or two-sided<br>*Only common tests should be described solely by name; describe more complex techniques in the Methods section.*
☐	☒	A description of all covariates tested
☐	☒	A description of any assumptions or corrections, such as tests of normality and adjustment for multiple comparisons
☐	☒	A full description of the statistical parameters including central tendency (e.g. means) or other basic estimates (e.g. regression coefficient) AND variation (e.g. standard deviation) or associated estimates of uncertainty (e.g. confidence intervals)
☐	☒	For null hypothesis testing, the test statistic (e.g. *F*, *t*, *r*) with confidence intervals, effect sizes, degrees of freedom and *P* value noted<br>*Give P values as exact values whenever suitable.*
☐	☒	For Bayesian analysis, information on the choice of priors and Markov chain Monte Carlo settings
☒	☐	For hierarchical and complex designs, identification of the appropriate level for tests and full reporting of outcomes
☐	☒	Estimates of effect sizes (e.g. Cohen's *d*, Pearson's *r*), indicating how they were calculated

Our web collection on statistics for biologists contains articles on many of the points above.

Software and code

Policy information about availability of computer code

Data collection	No software was used.
Data analysis	Software and packages used in data analysis throughout the manuscript:<br>1) Estimate the maximum lifespan and classify the long-lived species: R (v4.0.3), “finalfit” (v1.0.2), Phylopars (v0.3.0), “PVR” (v0.3), mice (v3.13.0), missForest (v1.4).<br>2) Calculate phylogenetic signal: “geiger” (v2.0.7),“ape” (v5.4.1),“phytools” (v0.7.70).<br>3) Compare evolutionary models of social organization and longevity: BayesTraits (v3) and Tracer (v1.7.1).<br>4) Analyze gene expression: using NGS QC Toolkit (v2.3.3) to remove low-quality reads of raw data; using Trinity (v2.8.6) and cd-hit (v4.8.1) to assemble transcripts; using AUGUSTUS (v3.3.3) to predict protein coding genes; using 'gffread' function in the 'cufflinks' package (v2.2.1) to extract CDS; using BLAST (v2.9.0+) to construct the orthologous gene set; using STAR (v2.7.1a) to map the RNA-seq reads to the reference genome; using featureCounts (v2.0.0) of package Subread (v2.0.0) to generate read counts from these alignments.<br>5) Remove batch effects and normalize raw counts: using comBat_seq function of the R package “sva” (v3.36.0); using 'edgeR' R package (v3.32.1) to perform normalization and generate the gene expression.<br>6) Identify significant genes associated with traits: using MCMCglmm function in the R package “MCMCglmm” (v2.29) to construct the MCMCglmm models; using gelman.diag function in “coda” (v0.19.4) to diagnose convergence; using the fitdist function and gofstat function of R package “fitdistrplus” (v1.1.1) to fit a high probability distribution.<br>7) Analyze gene selection features: MAFFT (v7.429), GBlock (v0.91b), HYPHY (v2.5.1)<br>8) Gene set enrichment: the pipeline 'polysel' (v1).<br>9) Statistical analyses and plots: R (v4.0.3), Rstudio (v1.2.1335), ggplot2 (v3.3.2), ggtree (v2.4.1), ggstar (v0.0.9), RColorBrewer (v1.1.2), tibble (v3.0.4), ggnewscale (v0.4.5), ggtreeExtra (v1.0.1), vioplot (v0.3.6), EnvStats (v2.4.0), ggpubr (v0.4.0), ggthemes (v4.2.0), scales (v1.1.1),

cowplot (v1.1.1), pheatmap (v1.0.12), dplyr (1.0.2), VennDiagram (1.6.20), ggpmisc (0.4.3).

For manuscripts utilizing custom algorithms or software that are central to the research but not yet described in published literature, software must be made available to editors and reviewers. We strongly encourage code deposition in a community repository (e.g. GitHub). See the Nature Portfolio guidelines for submitting code & software for further information.

Data

Policy information about availability of data

All manuscripts must include a data availability statement. This statement should provide the following information, where applicable:

- Accession codes, unique identifiers, or web links for publicly available datasets
- A description of any restrictions on data availability
- For clinical datasets or third party data, please ensure that the statement adheres to our policy

The RNA sequencing data generated in this study have been deposited in the Genome Sequence Archive in National Genomics Data Center, China National Center for Bioinformation / Beijing Institute of Genomics, Chinese Academy of Sciences under accession code GSA: CRA008468 [https://ngdc.cncb.ac.cn/gsa/browse/CRA008468].

The species traits data are provided in the Supplementary Data file. Databases used in the data collection of mammalian traits include PanTHERIA [https://esapubs.org/archive/ecol/E090/184/metadata.htm], PHYLACINE [https://zenodo.org/record/1250504#.Y5VZfnZBxnI], Animal Diversity Web [https://animaldiversity.org], GBIF [https://www.gbif.org/], ASM's Mammal Diversity Database [https://www.mammaldiversity.org/], the Encyclopedia of Life [https://eol.org/docs/what-is-eol], AnAge [https://genomics.senescence.info/species/index.html].

The phylogenetic tree is from TimeTree [https://timetree.org/].

In the comparative transcriptome analyses, we used databases NCBI [https://www.ncbi.nlm.nih.gov/], Ensembl [https://ensemblgenomes.org/], Gene Ontology (GO) [http://geneontology.org/], and Reactome [https://reactome.org/]. The SRA accession number and hyperlink of RNA-seq data that were not generated from this study were shown in Supplementary Data 2. Silhouette images of animals used in the figures are from PhyloPic database [http://phylopic.org/]. Source data are provided with this paper.

Human research participants

Policy information about studies involving human research participants and Sex and Gender in Research.

Reporting on sex and gender	This study did not involve human participants.
Population characteristics	This study did not involve human participants.
Recruitment	This study did not involve human participants.
Ethics oversight	This study did not involve human participants.

Note that full information on the approval of the study protocol must also be provided in the manuscript.

Field-specific reporting

Please select the one below that is the best fit for your research. If you are not sure, read the appropriate sections before making your selection.

- [x] Life sciences
- [ ] Behavioural & social sciences
- [ ] Ecological, evolutionary & environmental sciences

For a reference copy of the document with all sections, see nature.com/documents/nr-reporting-summary-flat.pdf

Life sciences study design

All studies must disclose on these points even when the disclosure is negative.

Sample size	Sample sizes are determined based on previous experience or similar published studies. At least two biological or technical replicates were performed for each trial to determine the reproducible results. Related reference: Aging Cell, 2015, 14, 352-365.<br>We collected 267 brain samples from 94 mammals in total, and 166 samples of 54 species were newly collected in this study (full details in Supplementary Data 2). About 96% of sampled individuals were adults, and 73% of individuals were males. The availability of samples determined the sex of sampled individuals. Given that we compared the gene expression difference at the species level rather than the individual level, we did not conduct sex-based analyses. Keeping a balance between the number of adult males and adult females within species was a priority if many individuals were available during sample collecting.<br>Since we aimed to characterize the conserved genes and pathways that are related to social organization and longevity among species, the mammal species were chosen based on the availability of brain transcriptome and life-history data, and also the representation of mammal diversity and taxa distribution in the phylogenetic tree. The average difference between the ratio of species of each order to all world mammals in nature and the ratio of sampled species of each order to 94 specie was 4.20% ± 6.33%. In addition, the mean longevity of the 974 mammals and 94 species was 19.55 ± 15.96 and 20.87 ± 16.53, respectively. The medians of longevity were 17.15 (IQR = 8.30 ~ 26.18) and 17.40 (IQR = 11.40 ~ 23.55). These indicate transcriptome species had a good taxa representation.
Data exclusions	No data were excluded from this study.

Replication	Three biological replicates were obtained if it was possible. About 71% of 94 species were prepared in more than two biological repeats or technical repeats. All replications were successful, and the number of repeats for each species is shown in Supplementary Data 2.
Randomization	We messed up the samples from different species before constructing the RNA-seq library. It is impossible to randomize all samples as some arrived at different times.
Blinding	Blinding was used when species were identified based on their morphological characteristics in the wild and were identified according to their sequences of the mitochondrial DNA cytochrome oxidase I gene (COI) or cytochrome b gene (cytb) in the lab.

Reporting for specific materials, systems and methods

We require information from authors about some types of materials, experimental systems and methods used in many studies. Here, indicate whether each material, system or method listed is relevant to your study. If you are not sure if a list item applies to your research, read the appropriate section before selecting a response.

Materials & experimental systems

n/a	Involved in the study
☒	☐ Antibodies
☒	☐ Eukaryotic cell lines
☒	☐ Palaeontology and archaeology
☐	☒ Animals and other organisms
☒	☐ Clinical data
☒	☐ Dual use research of concern

Methods

n/a	Involved in the study
☒	☐ ChIP-seq
☒	☐ Flow cytometry
☒	☐ MRI-based neuroimaging

Animals and other research organisms

Policy information about studies involving animals; ARRIVE guidelines recommended for reporting animal research, and Sex and Gender in Research

Laboratory animals	The study did not involve laboratory animals.
Wild animals	Given that the exact age of wild animals is unknown, we only identified whether an individual is an adult/subadult or not. About 96% of sampled individuals were adults. The individuals were caught accidentally during the wild animal investigations and population monitoring. The live-trapping method was used to catch and hold animals without harming them. Traps were checked frequently to avoid any injury. Chiroptera species were caught in custom harp traps as they left the roost and were initially placed in individual cloth bags. Except for the sampled adults, the rest of the individuals were released immediately in their original habitats. Species identification was performed based on their morphological characteristics and the sequences of the mitochondrial DNA cytochrome oxidase I gene (COI) or cytochrome b gene (cytb). Euthanasia methods, e.g., carbon dioxide ($CO2$) and/or barbiturates with local anesthetic, were used in animal experimentation. The brains were dissected, rapidly frozen in liquid nitrogen, and stored at −80 °C. Research specimens were collected and adhered to Chinese legal requirements and under the policy of the Animal Care and Use Ethics of the Institution.<br>The wild-collected samples list: Anourosorex squamipes, Aselliscus stoliczkanus, Cynopterus sphinx, Eonycteris spelaea, Erinaceus amurensis, Hipposideros armiger, Hipposideros larvatus, Hipposideros Pomona, Hipposideros pratti, Ia io, Kerivoula hardwickii, Miniopterus fuliginosus, Murina cyclotis, Murina hilgendorfi, Mustela sibirica, Myotis chinensis, Myotis fimbriatus, Myotis laniger, Myotis muricola, Myotis pilosus, Myotis siligorensis, Pipistrellus javanicus, Plecotus auritus, Rhinolophus affinis, Rhinolophus ferrumequinum, Rhinolophus luctus, Rhinolophus macrotis, Rhinolophus malayanus, Rhinolophus pearsonii, Rhinolophus pusillus, Rhinolophus rex, Rhinolophus sinicus, Rhinolophus stheno, Rhinolophus thomasi, Taphozous melanopogon, Tylonycteris robustula (see Supplementary Data 2).
Reporting on sex	Samples were collected from both males and females. The sex of sampled individuals was determined by the availability of samples. Since we compared the gene expression difference at the species level rather than the individual level, we did not conduct sex-based analyses. Keeping a balance between the number of adult males and adult females within species was a priority if many individuals were available during sample collecting. The sex of each sample was listed in Supplementary Data 2.
Field-collected samples	The field-collected samples were frozen in liquid nitrogen and stored at −80 °C prior to RNA extraction.
Ethics oversight	All animal care and research protocols were approved by the Institute of Zoology, Chinese Academy of Sciences (No. IOZ-IACUC-2021-129).

Note that full information on the approval of the study protocol must also be provided in the manuscript.
